# Glucose-induced STUB1-GOT2 axis promotes aspartate synthesis and mitochondrial dysfunction in bladder cancer

**DOI:** 10.1038/s41419-025-07840-5

**Published:** 2025-07-12

**Authors:** Yunqiang Xiong, Qianxi Dong, Hongji Hu, Zhongqi Li, Xiangpeng Zhan, Fuchun Zheng, Hao Wan, Jiahao Liu, Shuyu Wu, Wang Pan, Ruize Yuan, Jing Xiong, Ju Guo, Songhui Xu, Bin Fu

**Affiliations:** 1 Department of Urology, the First Affiliated Hospital, Jiangxi Medical College, Nanchang University, Nanchang, Jiangxi China; 2 Jiangxi Provincial Key Laboratory of Urinary System Diseases, Nanchang, Jiangxi China; 3 College of Chemical and Environmental Engineering, Shanghai Institute of Technology, Shanghai, China

**Keywords:** Cancer, Diseases

## Abstract

Aberrant glucose metabolism, a characteristic of malignant tumors, contributes to the development and progression of bladder cancer (BCa). However, the underlying mechanism by which aberrant glucose metabolism promotes BCa progression is still incompletely understood. Here, we demonstrate that low levels of STUB1 are associated with worse progression and poor prognosis of BCa patients. STUB1 overexpression attenuates BCa cell proliferation, migration and amino acid metabolism, especial aspartate metabolism. Mechanistically, we identify that STUB1 induces K6- and K48-linked polyubiquitination of GOT2 at K73 lysine residue to decrease its stability, which attenuates mitochondrial aspartate (Asp) synthesis and regulates mitochondrial dysfunction. GOT2 was significantly up-regulated in BCa tissues and negatively associated with STUB1 expression. Furthermore, we reveal that high glucose stress promotes Asp synthesis and tumor growth through STUB1-GOT2 axis. Collectively, our findings identify that STUB1-GOT2 axis is an important regulator for maintaining Asp synthesis and mitochondrial function in BCa cell growth, which highlights that targeting STUB1-GOT2 axis could be a valuable strategy to ameliorate BCa progression by inhibiting amino acid metabolic function.

## Introduction

Bladder cancer, a fifth most common cancer, a leading cause of cancer death worldwide and is associated with substantial impacts on patient quality of life, morbidity, mortality, and cost to the healthcare system [1, 2]. Aberrant glucose metabolism is one of the characteristics of malignant tumors including BCa [3]. Warburg et al. noted that cancer tissues maintained a higher glucose utilization rate than normal tissues [4]. Lattermann et al. analyzed glucose metabolism in patients with bladder cancer and observed that the plasma concentration of glucose was elevated in patients with BCa due to the lower rate of glucose clearance [5]. High glucose-mediated promotion of metastasis leads to death in patients with BCa [6]. However, the underlying mechanism by which high blood glucose promotes the tumorigenesis of BCa is still incompletely understood.

The glucose uptake is dramatically increased in cancer cells, and Glutamic-oxaloacetic transaminase (GOT) is inextricably intertwined with glucose metabolism which is closely related to the Warburg effect of tumor metabolism [7–9]. GOT exists as a cytoplasmic form, GOT1, and a mitochondrial form, GOT2 [10]. GOT2 catalyzes the conversion of glutamate and oxaloacetate to α-ketoglutarate (αKG) and aspartate (Asp) [11]. Several studies reported that GOT2 promotes progression of breast cancer [12], pancreatic cancer [13] and colorectal cancer [14] via Asp biosynthesis. Thus, the role of GOT2 as a promising and important therapeutic target has been increasingly recognized. However, research regarding specific GOT2 inhibitors and their potential therapeutic use in cancers remains very limited. Therefore, there is a great need to identify the key upstream factors control GOT2 expression. Here, we identified that STUB1 controls GOT2 stabilization and the mechanism of STUB1-GOT2 axis in regulating Asp biosynthesis and mitochondrial dysfunction, which can be exploited for potential therapeutic interventions.

Proteomic equilibrium including protein degradation controls mammalian cell biological function and maintains physiological environment stabilization, which is regulated through a comprehensive network, including molecular chaperone proteins, the ubiquitin-proteasome system [15]. STUB1, a functional E3 ubiquitin ligase, regulates the activity and stability of many oncogenes including stress inducible members that control cancer cell survival and progression [16–19]. STUB1 consists of an N-terminal TPR domain that interacts with chaperones, a central region, and a C-terminal U-box domain that recruits the E2 ubiquitin-conjugating enzyme [20]. Here, we identified U-box domain STUB1 interacts with GOT2 and STUB1 promotes GOT2 degradation through ubiquitin GOT2 at Lys 82 by promoting its K6/K48-linked ubiquitination. GOT2 degradation by STUB1 was antagonized by high glucose stimulation

In the present study, STUB1 was significantly downregulated in BCa tumor tissues, and low expression of STUB1 was associated with advanced progression and poor prognosis. Enforced STUB1 expression inhibits BCa cell proliferation and amino acid metabolomics, especial Asp metabolism. Mechanistically, we revealed that STUB1 promotes GOT2 degradation via K6/K48-linked ubiquitination. Moreover, we demonstrated that STUB1-GOT2 axis promotes mitochondrial Asp synthesis and regulates mitochondrial dysfunction, which was regulated by higher glucose stress. Our data highlight that targeting STUB1-GOT2 axis_induced Asp biosynthesis and mitochondrial dysfunction show a valuable strategy to inhibit BCa progression and may improve drug therapy in BCa patients.

## Materials and methods

### Cell lines and cell culture

HEK293T, T24, UM-UC-3 (UC3), SV-HUC-1, J28 and 5367 cells were maintained in Dulbecco’s modified Eagle’s medium (DMEM) or RPMI1640 with supplemented with 10% FBS, 100 units per ml penicillin, and 0.1 mg per ml streptomycin. All cells were obtained from the Bank of Type Culture Collection of Chinese Academy of Sciences, recently authenticated by fingerprinting of short tandem repeats, and tested negative for mycoplasma.

### Histological analysis

Histological analysis was performed as previously [21]. In brief, bladder cancer tissues fixed in 10% neutral-buffered formalin overnight, were processed using standard procedures and embedded in paraffin. The paraffin-embedded tissues were sectioned (5 µm), deparaffinized, rehydrated and stained with hematoxylin and Ki-67 by the Clinical Medical Research Center of the First Affiliated Hospital, Nanchang University. Histological analyses of Ki67-stained prostate tissues were conducted by a board certified pathologist. Cell proliferation index was calculated as the percentage of Ki67-positive nuclei to the total number of nuclei.

### Immunohistochemistry (IHC)

Tissue sample collection was approved by the Internal Review and Ethics Boards of the first affiliated hospital of Nanchang University. Prostate tissue microarrays were purchased Spector Biotech Inc. (Shandong, China). IHC staining assay was described previously [20].

### Metabolomics

The amino acid metabolites sequencing was outsourced to Shanghai Oebiotech. Two group samples (each group including 6 samples). The sample preparation steps are as follows: cells after 48 h of Flag-STUB1 plasmid transfection, 1) digestion with trypsin and transfer to a 15 mL centrifuge tube; 2) centrifugation at 1000 × *g*, 4 °C for 5 min, and removal of the supernatant; 3) washing with an appropriate amount of PBS 2-3 times, centrifugation at 4000 × *g*, 4 °C for 5 min, and removal of the supernatant; 4) additional wash with pre-cooled PBS, and cell counting in the PBS-containing cell suspension; 5) taking 1 × 107 cells/sample of cell suspension into a 1.5 mL sterile centrifuge tube; 6) low-speed centrifugation at 1000 × *g*, 4 °C for 10 min, and removal of the supernatant; 7) preserving the bottom of the centrifuge tube containing cell samples, rapid freezing in liquid nitrogen for 5 min, storage at −80 °C, and shipment with sufficient dry ice. The samples were used to GC-MS analysis (TAQ9000) and the data was analyzed by Shanghai Oebiotech (Shanghai, China).

### BALB/c nude mice model

All BALB/c nude male mice (4–6 weeks of age) were obtained from Charles River Laboratories in China (Beijing). All animals used in this study received humane care in compliance with applicable regulations, policies, and guidelines relating to animals. All experimental procedures using animals were approved by the Institutional Animal Care and Use Committee of the first affiliated hospital of Nanchang University. Control T24 cells (control) and STUB1 overexpression T24 cells (Flag-STUB1) were mixed with matrigel (1:1) and injected subcutaneously into the flanks of BALB/c nude male mice. Control T24 cells (control), STUB1 overexpression T24 cells (Flag-STUB1), GOT2 knockdown T24 cells (shGOT2) and STUB1 overexpression combined GOT2 knockdown T24 cells (Flag-STUB1 + shGOT2) were mixed with matrigel (1:1) and injected subcutaneously into the flanks of BALB/c nude male mice. After 21 days, tumors were weight, photographed and measured using calipers and tumor volumes were calculated using length × width × width × 0.5. Tumor tissues were used for western blotting analysis and Ki67 staining. Control T24 cells (control) and STUB1 overexpression T24 cells (Flag-STUB1) were mixed with matrigel (1:1) and injected subcutaneously into the flanks of BALB/c nude male mice. When tumors reached 40–75 mm^3^, mice were randomized into three groups (twelve mice per group): (1) control (feed with 100% H_2_O), (3) Flag-STUB1 (feed with 100% H_2_O), and (3) Flag-STUB1 + HG (feed with 10 g/L glucose). Tumors were measured and tumor volumes were calculated as above described.

### Statistical analysis

Statistical analyses were performed with Prism 8.0 (GraphPad Software). For cell proliferation, cell colony formation and cell soft agar colony formation, data were analyzed by unpaired Student’s *t* test. Comparisons between Kaplan–Meier curves were performed using the long-rank test. Correlations were determined by Pearson correlation. The differences with ^*^p < 0.05 or ^**^p < 0.01 were considered statistically significant.

Additional materials and methods are presented in the Supplemental Materials.

## Results

### STUB1 is frequently downregulated in BCa and correlates with poor prognosis in human BCa

**Fig. 1:**
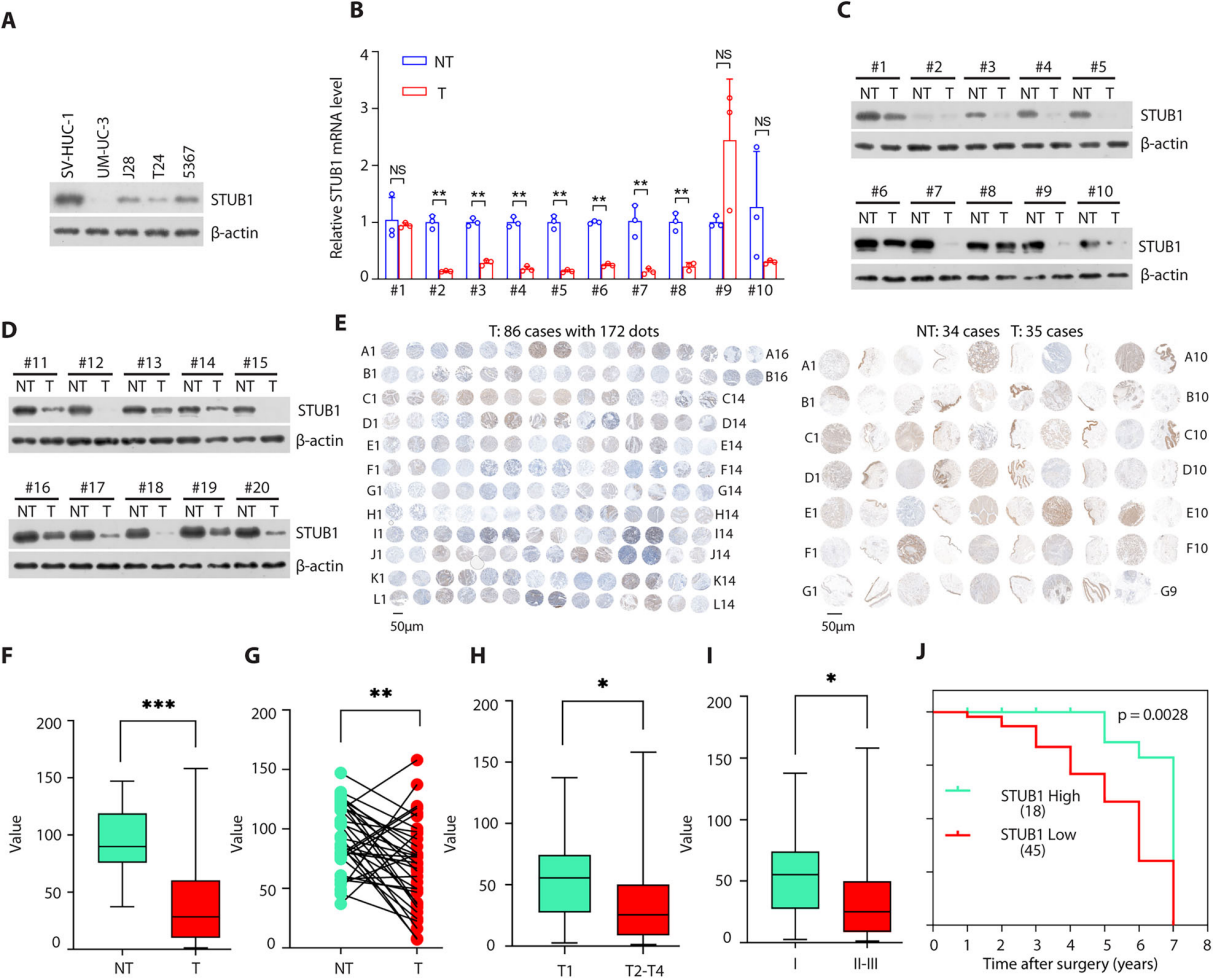
STUB1 is frequently downregulated in BCa. **A** Expression of STUB1 was detected by western blotting in BCa cell lines. **B** STUB1 mRNA expression in ten paired adjacent non-tumor tissues and matched BCa tissues was detected by Real-time PCR. The *p* values were obtained by two-tailed unpaired *t* test. ^*^p < 0.05, ^**^p < 0.01, N.S means not significant. **C**, **D** STUB1 protein expression in twenty paired adjacent non-tumor tissues and matched BCa tissues was analyzed by western blotting. **E** IHCs of two tissue chips including human BCa tissues (*n* = 121) and adjacent non-tumor tissues (*n* = 34) were performed using STUB1 antibody. **F** STUB1 protein expression was downregulated in adjacent non-tumor tissues and BCa tissues. **G** STUB1 protein expression was downregulated in paired adjacent non-tumor tissues and matched PCa tissues. A low STUB1 expression was related with a higher preoperative clinical T2-T4 stage (**H**), Tissue II-III grade (**I**) and a lower overall survival of human BCa patients (**J**).

We and others previously revealed that STUB1 play a vital role in urinary system diseases [15, 20, 22]. To investigate the role of STUB1 in BCa, we analyzed STUB1 expression in various BCa cell lines and one normal bladder epithelial cell line (SV-HUC-1) via western blotting. We found that STUB1 were downregulated in BCa compared with normal cells (Fig. 1A). Next, we sought to determine the STUB1 mRNA levels in human BCa tissues and revealed that STUB1 mRNA levels were downregulated (7/10) in BCa specimens compared with adjacent non-tumor tissues (Fig. 1B). Consistently, we also determined the STUB1 protein expression in BCa tissues and paired adjacent non-tumor tissues in our center by western blotting and found that the expression of STUB1 was evidently lower in BCa (Fig. 1C, D). We next assessed the expression of STUB1 protein in two tissue microarrays including 121 samples of BCa tissues and 34 samples of adjacent non-tumor tissues (Fig. 1E). Immunohistochemical analysis demonstrated that lower protein levels of STUB1 than in adjacent non-tumor tissues (Fig. 1F, G) (Supplementary Tables S1 and S2). Importantly, downregulation of STUB1 protein expression was correlated with tumor size (Fig. 1H) and poor histological grade (Fig. 1I), not correlated with age (Supplementary Fig. S1A) and metastasis (Supplementary Fig. S1B). Notably, BCa patients with low STUB1 expression had worse outcomes than those with high STUB1 expression (Fig. 1J). These data indicate a negative correlation of STUB1 expression with worse progression and poor prognosis of BCa patients.

### STUB1 overexpression inhibits BCa growth in vitro and in vivo

**Fig. 2:**
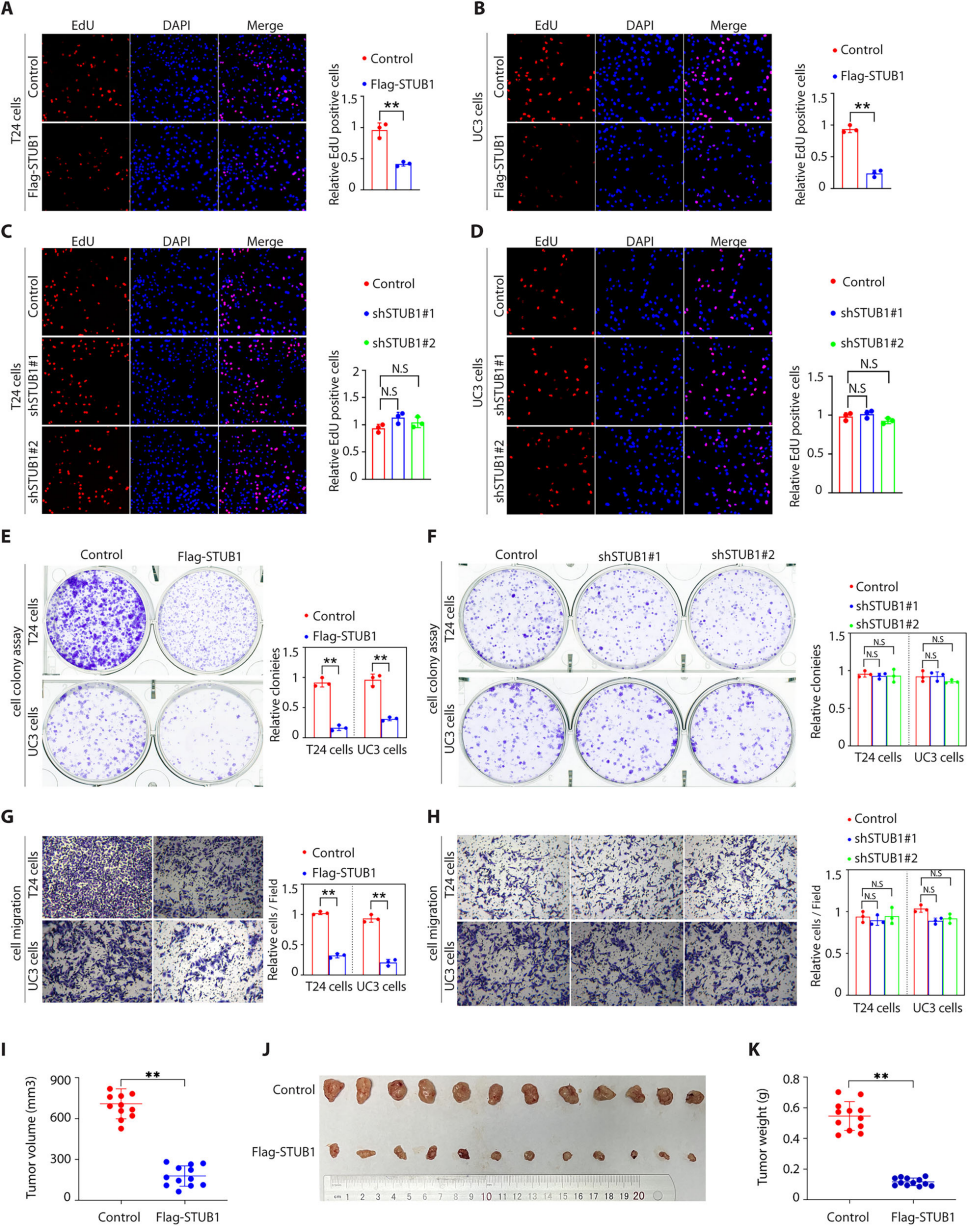
STUB1 overexpression inhibits BCa cell growth in vitro and in vivo. **A**–**H** The indicated T24 and UC3 cells which were overexpression or knockdown of STUB1 were performed by EdU assays (**A**–**D**), colony formation assays (**E** and **F**), and the motility was examined by migration assay (**G** and **H**). **I**–**K** Cells generated as described in Methods were injected into the BALB/c nude mice. The tumor sizes (**I**), images (**J**) and tumor weights (**K**) of subcutaneous xenografts are presented. Data are presented as mean ± SD. The *p* values were obtained by two-tailed unpaired *t* test or two-way ANOVA. ^*^p < 0.05, ^**^p < 0.01, N.S means not significant.

To explore the functional role of STUB1 in BCa, we overexpressed STUB1 by transfecting Flag-STUB1 plasmids (Supplementary Fig. S2A), and knocked down STUB1 expression via two STUB1-specific shRNA lentivirus (Supplementary Fig. S2B) in T24 and UC3 cells. STUB1 overexpression decreased the proliferation and motility of T24 and UC3 cells as determined through Edu assays (Fig. 2A, B), but STUB1 knockdown not increased cell proliferation (Fig. 2C, D). Similar results were observed by cell colony formation (Fig. 2E, F) and cell motility by migration assay (Fig. 2G, H). Similar, we knocked down STUB1 in the SC-V-HUC1 cells which express high levels of STUB1 and detect the cell colony formation and cell motility, we also found STUB1 knockdown did not affect cell colony formation and cell motility (Supplementary Fig. S2C–E). We then extended this observation to the in vivo xenograft tumor model derived from T24 cells after overexpression of STUB1 and found that consistent with cell culture model, STUB1 overexpression inhibited tumor growth (Fig. 2I–K). We also confirmed that STUB1 were indeed overexpressed in respective tumor tissues (Supplementary Fig. S2F) and the marker protein for proliferation (Ki67) in the tumor tissues of nude mice was decreased compared with control group (Supplementary Fig. S2G). Taken together, STUB1 is relatively a strong tumor suppressor in BCa cells.

### STUB1 interacts with GOT2 via its U-box domain

**Fig. 3:**
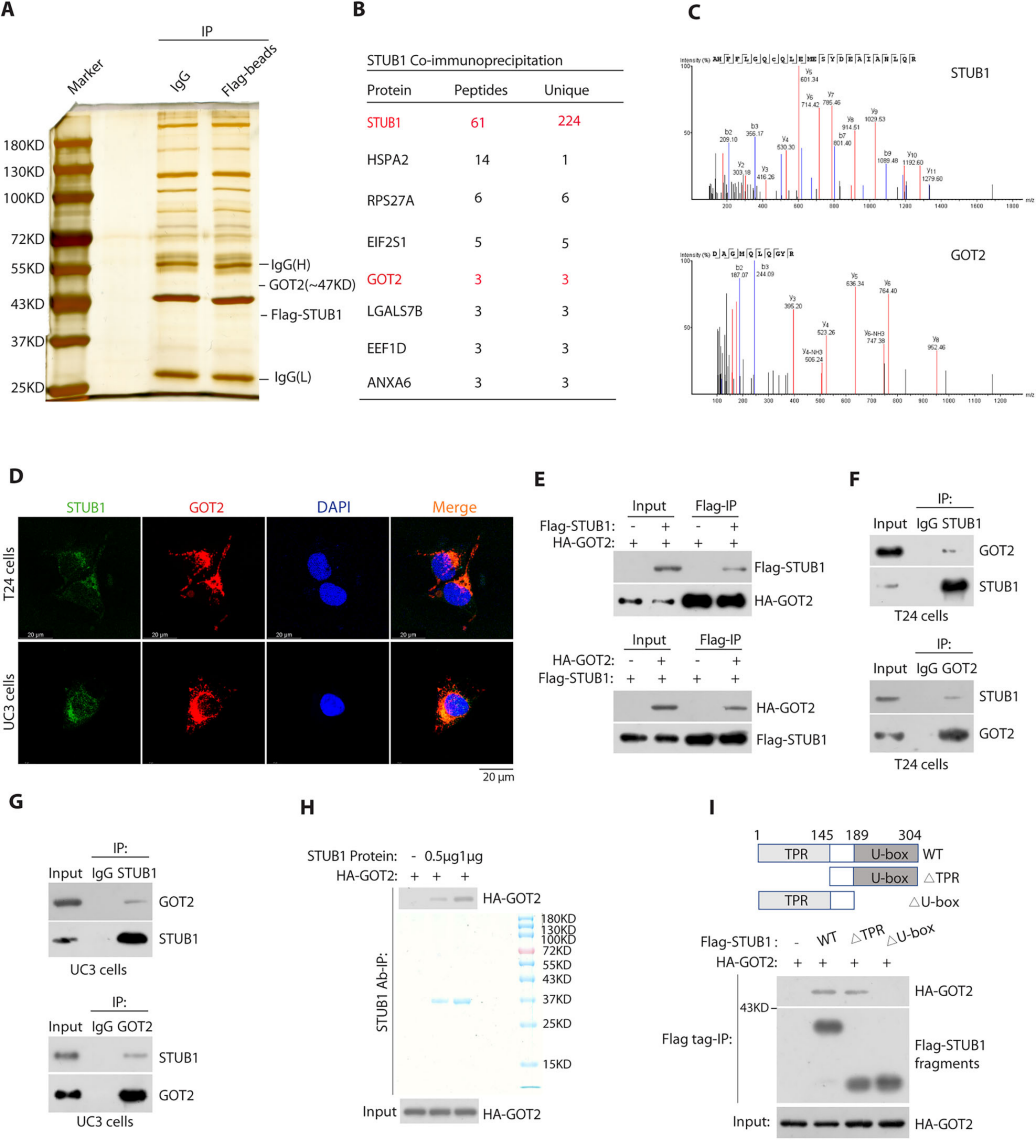
STUB1 directly interacts with GOT2. **A** The HEK293T cells were lysed and subjected to immunoprecipitation using anti-Flag Magnetic Beads. The complexes were then separated, and the gels were stained with silver. **B** List of STUB1-associated proteins were identified peptides and unique peptides more than or equal three by mass spectrometric analysis. **C** Representative best unique peptides of STUB1 and GOT2 were identified by mass spectrometry assays. **D** Immunostaining of STUB1 (green) and GOT2 (red) were detected by their antibody in both UC3 and T24 cells. Nuclear 4’, 6-diamidino-2-phenylindole (DAPI; blue). **E** HEK293T cells were co-transfected with HA-GOT2 and Flag-tagged STUB1 plasmids, and cell lysates were subjected to IP and detected with the indicated tagged antibodies. **F**, **G** UC3 or T24 cell lysates were subjected to immunoprecipitation with control IgG, anti-STUB1 or anti-GOT2 antibody. The immunoprecipitates were then blotted with the indicated antibodies. **H** Purified recombinant STUB1 proteins were incubated with extracts of HA- GOT2-transfected HEK293T cells, and then immunoblotted with the antibody to HA-tag antibody. Bottom, recombinant STUB1 protein were purified from bacteria and analyzed by SDS-PAGE and coomassie blue staining. **I** HEK293T cells were cotransfected with HA- GOT2 plasmids and Flag-tagged full-length STUB1 plasmids or its deletion mutant plasmids for 24 h, and then cell lysates were immunoprecipitated with anti-Flag magnetic beads, and immunoblotted with antibodies to HA and Flag tag antibody.

Given that the integrated anti-tumor potency of STUB1 is enzymatically dependent, it is essential identify potential substrates, which correlated aspartate metabolism. To this end, we captured Flag-STUB1 and its associated proteins from BCa cell lysates using anti-Flag magnetic beads (Fig. 3A). Mass spectrometry analysis (Fig. 3B, C) (Supplementary Table S3) showed that among the TOP8 interacted proteins (identified total peptides and unique peptides ≥3), only GOT2, a mitochondrial malate aspartate shuttle is essential for maintaining glycolysis and energy metabolism in tumors [23–25]. We select GOT2 for further analysis for several reasons. Our targeted amino acid metabolomics analysis showed that STUB1 overexpression significantly reduces aspartate synthesis by Volcano analysis (Supplementary Fig. S3A), Heatmap analysis (Supplementary Fig. S3B), KEGG pathway analysis (Supplementary Fig. S3C) and chart analysis (Supplementary Fig. S3D, Supplementary Table S4). Second, immunofluorescence staining revealed that endogenous STUB1 and GOT2 were primarily colocalized in the cytoplasm (Fig. 3D) and we verified that Flag-tagged STUB1 or HA-tagged GOT2 was readily detected in either HA-tagged GOT2 or Flag-tagged STUB1 immunoprecipitates in HEK293T cells (Fig. 3E), endogenous STUB1 and GOT2 coprecipitated in both T24 and UC3 cells (Fig. 3F, G). Additionally, we found that purified STUB1 was able to bind to HA-tagged GOT2 under cell-free conditions (Fig. 3H). STUB1 consists of an N-terminal TPR domain that interacts with chaperones, a central region, and a C-terminal U-box domain that recruits the E2 ubiquitin-conjugating enzyme (Fig. 3I). To map which domain of STUB1 interacts with GOT2, we performed co-immunoprecipitation (co-IP) experiments using truncated mutants of STUB1 (Fig. 3I). HA-GOT2 co-precipitated with mutant STUB1 lacking the TPR domain, but not a mutant lacking the U-box domain (Fig. 3I). Together, these results revealed that GOT2 interacts with STUB1 U-box domain.

### STUB1 negatively regulates GOT2 stability through ubiquitin GOT2 at Lys 73 by promoting its K6/K48-linked ubiquitination

**Fig. 4:**
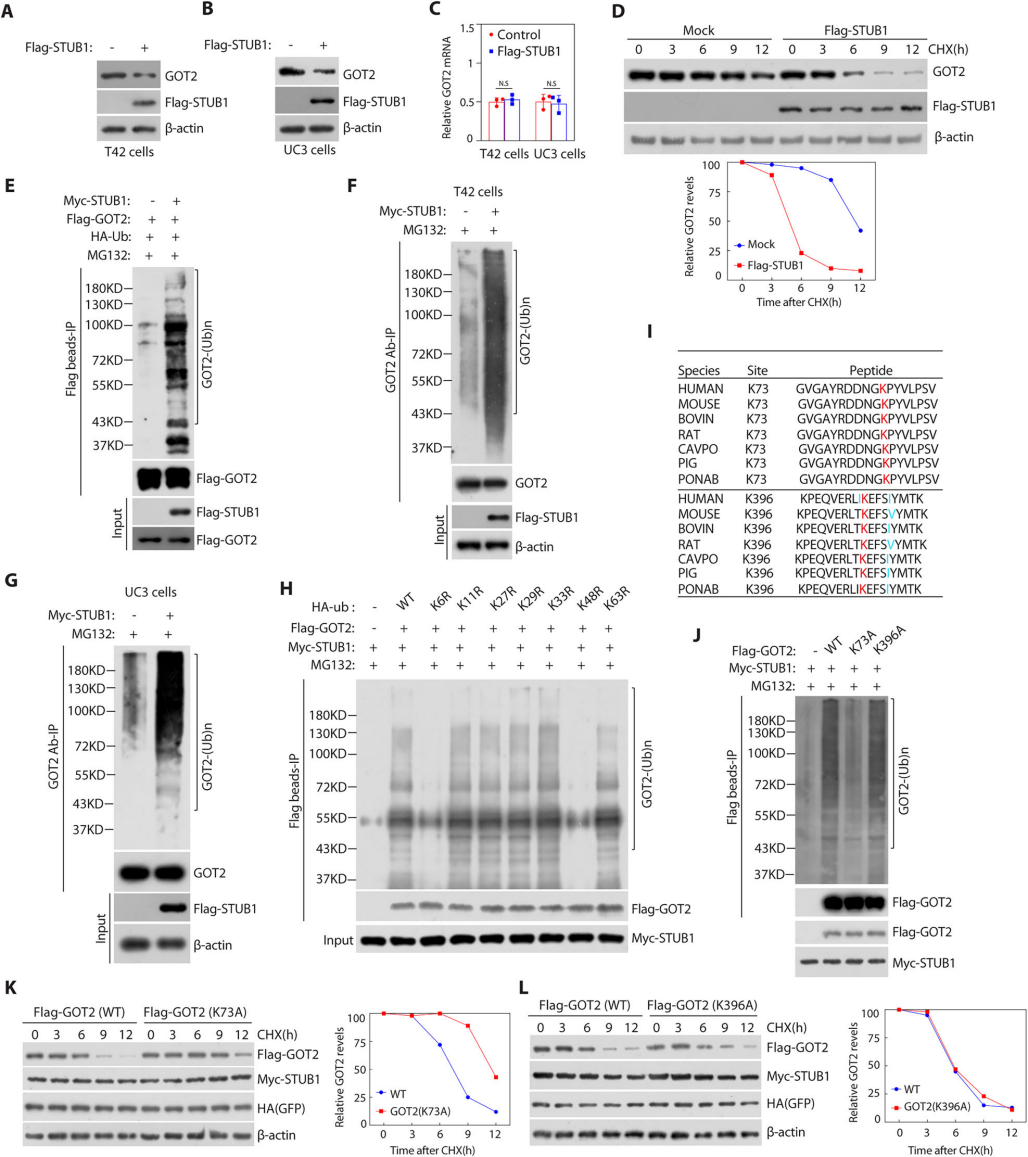
STUB1 promotes GOT2 degradation by ubiquitin. **A**–**C** T24 or UC3 cells transfected with Flag-STUB1 plasmids for 48 h, the cell were analyzed by western blotting and qRT-PCR assay. **D** HEK293T cells were transfected with Flag-STUB1 plasmids, treated with 50 mg/mL cycloheximide, harvested at different time points, and then immunoblotted with antibodies to GOT2, Flag tag and β-actin. Bottom, quantification of GOT2 protein levels (normalized to β-actin). **E** Immunoblotting analysis of the ubiquitination of GOT2 in HEK293T cells were co-transfected with Flag-GOT2, HA-ub plasmids cells ectopic expression of Myc-STUB1 or not. Cells were treated with 20 μM MG132 for 6 h before harvesting. **F**, **G** Immunoblotting analysis of the ubiquitination of GOT2 in T24 or UC3 cells transfected with or without Myc-STUB1 plasmids. Cells were treated with 20 μM MG132 for 6 h before harvesting. **H** HEK293T cells were transfected with Myc-STUB1, Flag-GOT2, and HA-ub (WT, K6R mutant, K11 mutant, K27 mutant, K29 mutant, K33 mutant, K48 mutant, and K63 mutant). The analysis was undertaken as described for (**E**). **I** Prediction ubiquitin site of GOT2 by alignment of the similarity of GOT2 sequences across multiple species. **J** Immunoblotting analysis of the ubiquitination of GOT2 in HEK293T cells were co-transfected with Myc-STUB1 plasmids and ectopic expression of GOT2 (wild type, K73A and K396A) or not. **K**, **L** HEK293T cells were co-transfected with Myc-STUB1 plasmids, HA-GFP plasmids (internal control) and ectopic expression of GOT2 (wild type, K82A or K364A), treated with 50 mg/mL cycloheximide (CHX), harvested at different time points, and then immunoblotted with antibodies to Flag tag, Myc tag, HA tag and β-actin. Right, quantification of GOT2 protein levels (normalized to β-actin).

Having established that STUB1 interacts with GOT2 and STUB1 was an E3 ubiquitin ligase, we next determined the effect of STUB1 manipulations on GOT2 levels. Ectopic expression of STUB1 decreased the protein levels of endogenous GOT2 in both T24 and UC3 cells (Fig. 4A, B), but not affect the mRNA levels of GOT2 (Fig. 4C), and markedly reduced GOT2 protein half-life (Fig. 4D). Thus, the stability of GOT2 is negatively regulated by STUB1. We then determined whether STUB1 promoted the ubiquitination of GOT2. Ectopic expression of STUB1 promoted the poly ubiquitination of ectopic expression of GOT2 in HEK293T cells (Fig. 4E) and also reduced the polyubiquitination of endogenous GOT2 in both T24 and UC3 cells (Fig. 4F, G). Using various ubiquitin mutants, we found that STUB1-mediated GOT2 polyubiquitination is via the K6/K48 linkage for targeted degradation (Fig. 4H). To identify the specific ubiquitination modification lysine sites in the GOT2 protein, we searched ubPred software (https://gpsuber.biocuckoo.cn/). Two high score potential ubiquitination sites at lysine residues (K73 and K396) were found in the GOT2 protein (Supplementary Table S5). Additionally, the Prediction of K73 lysine residue ubiquitin site is more conservation then K396 by alignment of the similarity of GOT2 sequences across multiple species (Fig. 4I). We subsequently constructed GOT2 mutants in which these lysine residues were replaced with alanine. Our results showed that overexpression of STUB1 increased GOT2 mutant polyubiquitination levels with K396A, but not in the mutant with K73A (Fig. 4J). Meanwhile, we demonstrated that the K73A GOT2 protein was more stable than wild type GOT2 or K396A GOT2 protein after overexpressed STUB1 in 293T cells (Fig. 4K, L). Collectively, these data indicate that STUB1 negatively regulates GOT2 stability through ubiquitin GOT2 at Lys 73 by promoting its K6/K48-linked ubiquitination.

### STUB1 negatively correlates with GOT2 in BCa samples and STUB1 overexpression-mediated GOT2 degradation in vivo

**Fig. 5:**
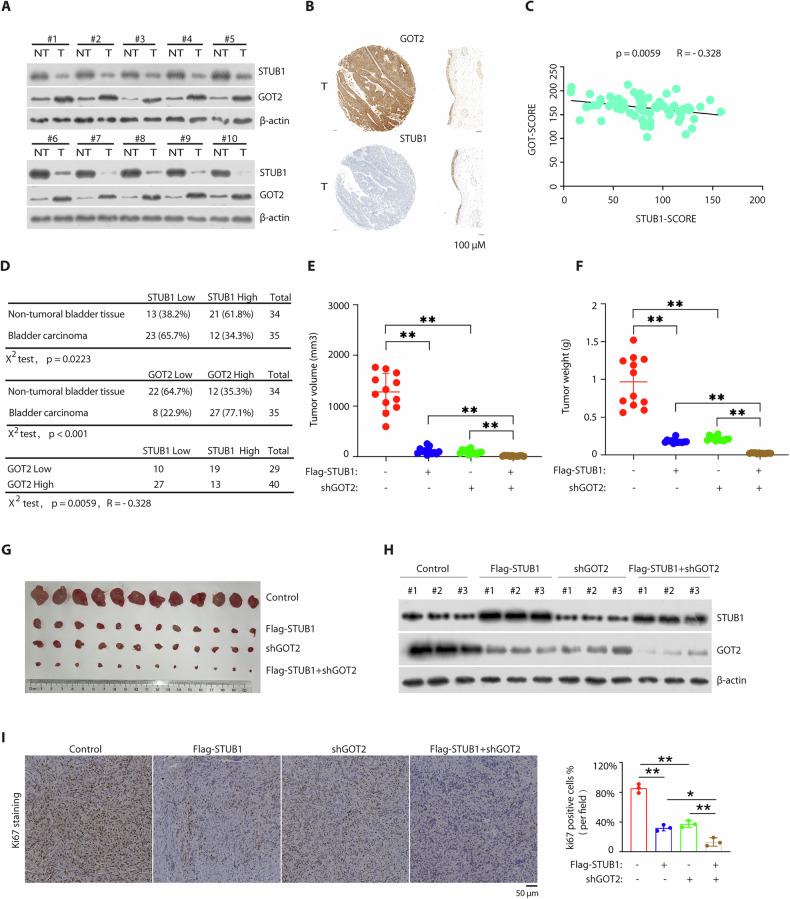
STUB1 negatively correlates with GOT2 in BCa samples and STUB1 overexpression-mediated GOT2 degradation in vivo. **A** Immunoblotting analysis with indicated antibodies in 10 BCa tissues (T) and paired adjacent non-tumor tissues (NT). **B** A representative IHC image showed a low STUB1 negatively with a high GOT2 abundance in one clinical BCa sample. **C**, **D** Quantification of STUB1 and GOT2 protein levels in adjacent non-tumor tissues and BCa tissues, and the correlation study of STUB1 and GOT2 expression level in BCa tissues. Statistical analyses were undertaken with the χ^2^ test, ^*^p < 0.05, ^**^p < 0.01^.^ R, pearson’s correlation coefficient. **E**–**G** The nude mice were performed by subcutaneous injection of T24 cells stably expressing control plasmids, Flag-STUB1 plasmids, GOT2 shRNA plasmids and or Flag-STUB1 plasmids + GOT2 shRNA. Tumor growth curve of each group and tumor weights were measured as shown in (**E**) and (**F**). Photographs of mice tumors of each group (*n* = 12) at the end of the experiment (**G**). **H**, **I** Expression of STUB1 and GOT2 were analyzed by western blotting in the tumors from (**G**) (each group *n* = 3). Representative staining of Ki67 on the tumor sections derived from above mice. The staining was developed by DAB (brown) and counterstained by hematoxylin (blue). Statistical analyses were performed with the ANOVA, ^*^p < 0.05; ^**^p < 0.01 and data are shown as mean ± SD.

To better characterize the role of STUB1 in BCa, we then examined the expression of STUB1 and GOT2 in BCa tissues by western blotting. Low STUB1 protein levels correlated with decreased GOT2 expression in most BCa tissues samples (Fig. 5A). To further confirm the results, we also performed immunohistochemical staining of GOT2 in tissue microarray, including 35 samples of BCa tissues and 34 paired adjacent non-tumor tissues, which was used to stain of STUB1 (Supplementary Fig. S4) (Supplementary Table S6). Moreover, we observed that lower expression of STUB1 was negatively associated with stronger expression of GOT2 in BCa tissues (Fig. 5B). Pearson’s correlation analyses revealed a significant negative correlation between the expression scores of STUB1 and GOT2 (R = −0.328, *p* = 0.0059) (Fig. 5C). Of the 35 BCa tissue samples, 23 cases showed weak STUB1 and 27 cases showed strong GOT2 expression (Fig. 5D). These results indicate that STUB1 is downpregulated in BCa tissues and negatively correlates with GOT2 expression, suggesting that STUB1 may inhibit BCa progression through GOT2.

### The STUB1- GOT2 axis regulates growth and survival of BCa cells

We next investigated the effect of the STUB1-GOT2 axis on BCa cell growth and survival in vivo xenograft tumor model derived from T24 cells after overexpression of STUB1 or shRNA-based knockdown of GOT2 or both and found that STUB1 overexpression or GOT2 knockdown inhibited tumor growth, while co-overexpression of STUB1 and GOT2 knockdown enhance this effect (Fig. 5E–G),

demonstrating a dominant role of STUB1- GOT2 axis in controlling of in vivo tumor growth. We also confirmed that STUB1 or GOT2 were indeed overexpressed or knocked down and STUB1 overexpression increased GOT2 expression in respective tumor tissues (Fig. 5H). Additionally, the marker protein (Ki67) expression for proliferation in the tumor tissues of nude mice showed a similar result as the above tumor growth (Fig. 5I). Taken together, STUB1 appears to be a strong suppressor, whereas GOT2 is a tumor oncogenic protein in BCa cells.

### STUB1-GOT2 axis promotes mitochondrial Asp synthesis and regulates mitochondrial dysfunction

**Fig. 6:**
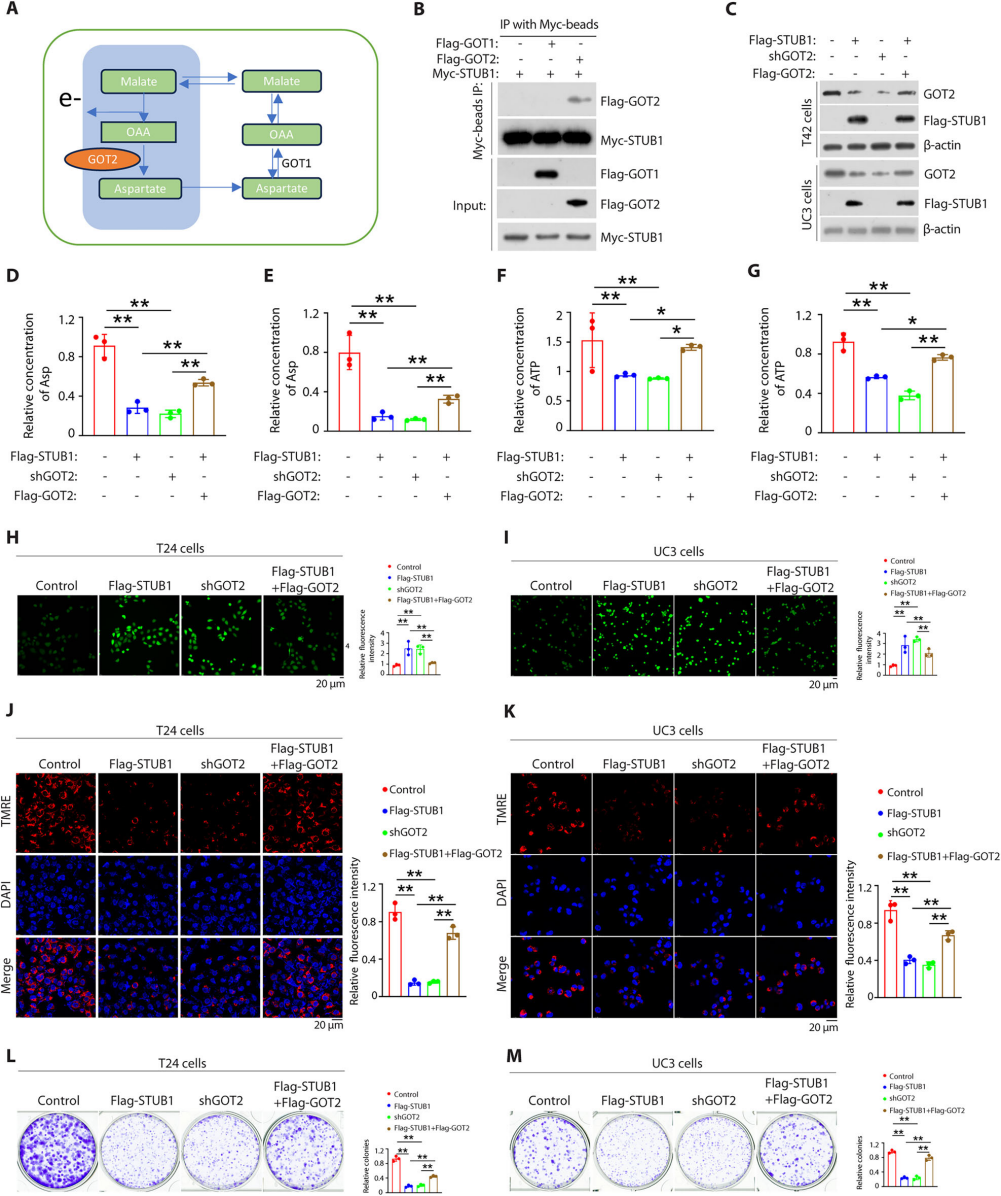
STUB1-GOT2 axis promotes mitochondrial aspartate (Asp) synthesis and regulates mitochondrial dysfunction. **A** The schematic showing the metabolic process of Asp. **B** The HEK293T cells transfected with the indicated plasmids for 48 h, then cell lysates were immunoprecipitated with anti-Myc magnetic beads, and immunoblotted with the indicated antibodies. **C** T24 or UC3 cells transfected with indicated plasmids for 48 h, Cell lysates were analyzed by western blotting using the indicated antibodies. T24 cells (**D**) or UC3 cells (**E**) were transfected with indicated plasmids for 48 h, Cell lysates were analyzed by Asp assay kit. T24 cells (**F**) or UC3 cells (**G**) were transfected with indicated plasmids for 48 h, Cell lysates were analyzed by ATP assay kit. **H**, **I** Immunofluorescent staining of reactive oxygen species (blue) with ROS Assay Kit in the T24 cells (**J**) or UC3 cells (**K**) transfected with indicated plasmids. **J**, **K** Immunofluorescent staining of mitochondrial membrane potential (red) with mitochondrial membrane potential assay Kit and nuclear (blue) with Nuclear 4’, 6-diamidino-2-phenylindole in the T24 cells (**H**) or UC3 cells (**I**) transfected with indicated plasmids. **L**, **M** T24 cells (**F**) or UC3 cells (**G**) were transfected with indicated plasmids for 48 h and then the cells were performed by colony formation assay. The *p* values were obtained by two-tailed unpaired *t* test. ^*^p < 0.05, ^**^p < 0.01.

The biosynthesis of aspartate is central in cancer cell autonomous proliferation [11]. Aspartate biosynthesis is driven largely by glucose- or glutamine-dependent refilling of the tricarboxylic acid (TCA) cycle to replenish mitochondrial TCA intermediate oxaloacetate, which is subsequently converted to aspartate through the activity of mitochondrial glutamic-oxaloacetic transaminase (GOT2) (Fig. 6A) or use reductive glutamine metabolism to generate aspartate through the cytosolic GOT1 isoform [11, 26]. To determine whether STUB1 regulates Asp synthesis through GOT2, we firstly stably expressed Myc-STUB1 plasmids together with or without Flag-GOT2 or Flag-GOT1 plasmids in HEK293T cells. We found that STUB1 interact with GOT2, not GOT1, and GOT2 knockdown reduced their interaction (Fig. 6B, C). STUB1 overexpression or GOT2 suppression decreased Asp synthesis, whereas GOT2 overexpression can significantly rescued the effect of STUB1 overexpression in both T24 and UC3 cells (Fig. 6D, E). Since the key bioenergetic indicators are adenosine triphosphate (ATP) synthesis and reactive oxygen species (ROS) production [27]. We firstly determine whether STUB1-GOT2 axis regulates ATP synthesis and ROS production. We revealed that STUB1 overexpression or GOT2 suppression decreased ATP synthesis, whereas GOT2 overexpression can significantly rescued the effect of STUB1 overexpression in both T24 and UC3 cells (Fig. 6F, G). A similar result was observed in detecting ROS production (Fig. 6H, I). GOT2 catalyzes mitochondrial membrane potential oxaloacetate to aspartate and depolarization of mitochondrial membrane potential has been widely reported as a hallmark event of mitochondrial dysfunction for cell death [28, 29]. Then, we explored whether STUB1-GOT2 axis promotes cell death through regulating mitochondrial dysfunction. To do so, we firstly overexpressed STUB1, silenced GOT2 or overexpressed GOT2 in the BCa cells of STUB1 overexpression to detect cell proliferation and the marker proteins of caspase (Bax and Bcl-2). We found that STUB1 overexpression or GOT2 suppression decreased cell proliferation, whereas GOT2 overexpression can significantly rescued the effect of STUB1 overexpression in both T24 and UC3 cells (Supplementary Fig. S5A–D). Furthermore, we revealed that STUB1 overexpression or GOT2 suppression decreased mitochondrial membrane potential, whereas GOT2 overexpression can significantly rescued the effect of STUB1 overexpression in both T24 and UC3 cells (Fig. 6J, K). A similar result was observed in cell colonic formation (Fig. 6L, M). Collectively, these data demonstrate that STUB1-GOT2 signal axis promotes mitochondrial Asp synthesis and regulates mitochondrial dysfunction.

### Glucose promotes Asp synthesis and tumor growth through STUB1-GOT2 axis

**Fig. 7:**
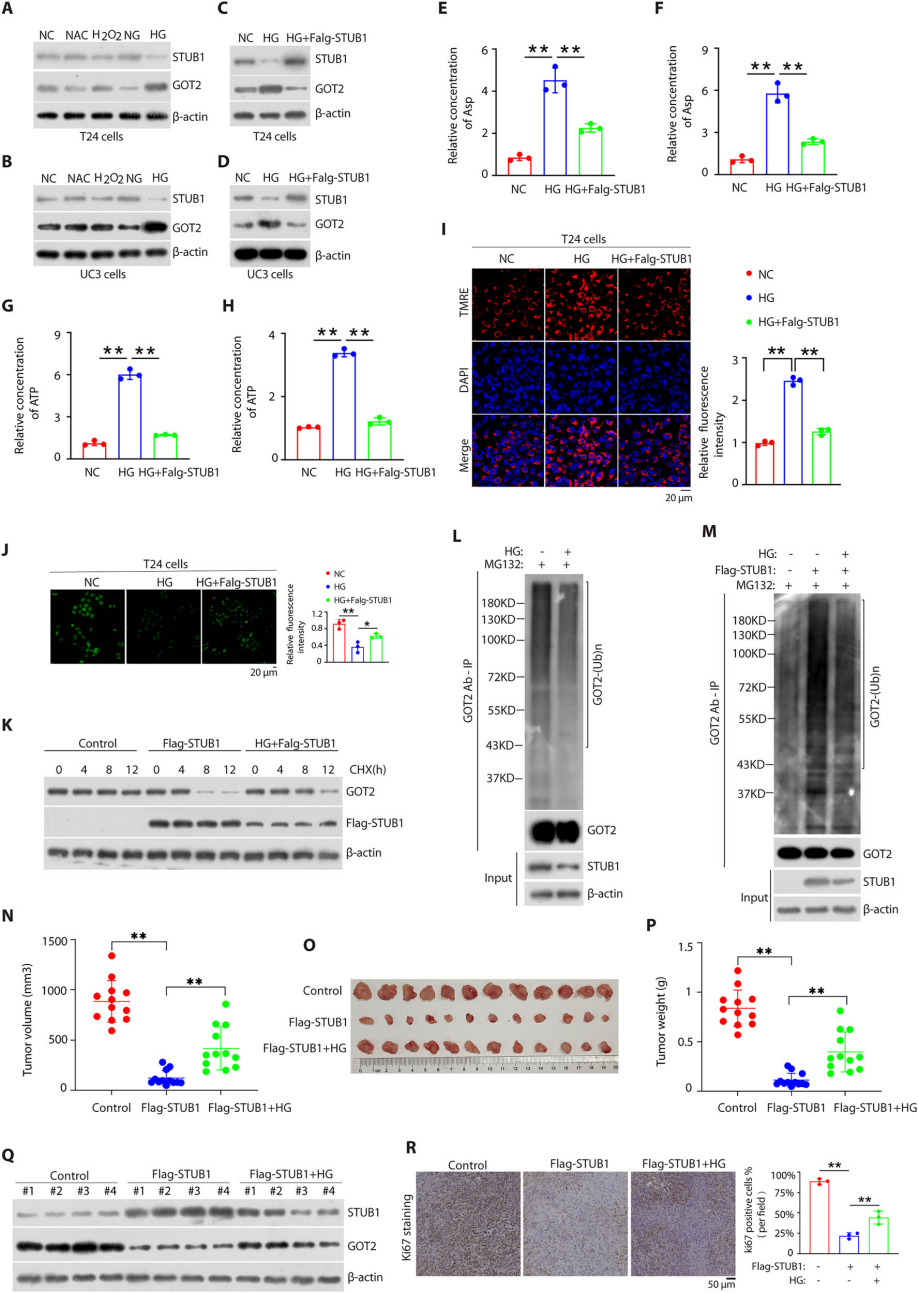
High glucose promotes Asp synthesis and tumor growth through STUB1-GOT2 axis. Western blotting analysis of both T24 cells (**A**) or UC3 cells (**B**) after stimulation with 10 mM NAC (N-acetyl-L-cysteine), 100 μM H_2_O_2_, 25 mM glucose or 0 g/L glucose for 6 h in serum free medium and before treatment, these cells were harvested for 6 h. Western blotting analysis of both T24 cells (**C**) or UC3 cells (**D**) with or without overexpressing STUB1 after stimulation with 4.5 g/L glucose for 6 h in serum free medium and before treatment, these cells were harvested for 6 h. The T24 cells (**E**) or UC3 cells (**F**) were described as (**C**) or (**D**) and the cell lysates were analyzed by Asp assay kit. The T24 cells (**G**) or UC3 cells (**H**) were described as (**C**) or (**D**) and the cell lysates were analyzed by Asp assay kit. **I** Immunofluorescent staining of mitochondrial membrane potential (red) with mitochondrial membrane potential assay Kit and nuclear (blue) with Nuclear 4’, 6-diamidino-2-phenylindole in the T24 cells with or without overexpressing STUB1 after stimulation with 4.5 g/L glucose for 6 h in serum free medium. **J** Immunofluorescent staining of reactive oxygen species (blue) with ROS Assay Kit in the T24 cells described as (**I**). **E**–**J** *p* values were obtained by two-tailed unpaired *t* test, ^*^p < 0.05, ^**^p < 0.01. **K** T24 cells were transfected with Flag-STUB1 plasmids for 48 h and then treated with or without 4.5 g/L glucose for 6 h in serum free medium. Before harvested, cells were treated with 50 mg/mL cycloheximide (CHX) and then immunoblotted with antibodies to GOT2, Flag tag and β-actin. **L** Immunoblotting analysis of the ubiquitination of GOT2 in T24 cells were treated with or without 4.5 g/L glucose for 6 h in serum free medium. Cells were treated with 20 μM MG132 for 6 h before harvesting. **M** Immunoblotting analysis of the ubiquitination of GOT2 in T24 cells with overexpressing STUB1 were treated with or without 4.5 g/L glucose for 6 h in serum free medium. Cells were treated with 20 μM MG132 for 6 h before harvesting. **N**–**R** The indicated T24 cells generated as described in Methods were injected into the BALB/c nude mice. The in vivo tumor growth was measured and plotted (mean ± SEM, *n* = 12) (**N**). Tumors were harvested at the end of experiment, photographed (**O**), weighted (**P**), and tumor tissues (*n* = 3) were lysed, followed by immunoblotting (**Q**), and Representative images of Ki67 (marker proteins of proliferation) staining in consecutive tumor tissues. Two-way ANOVA/Tukey’s multiple comparison tests for (**N**). Two-tailed, unpaired, *t* test for (**P**).

**Fig. 8:**
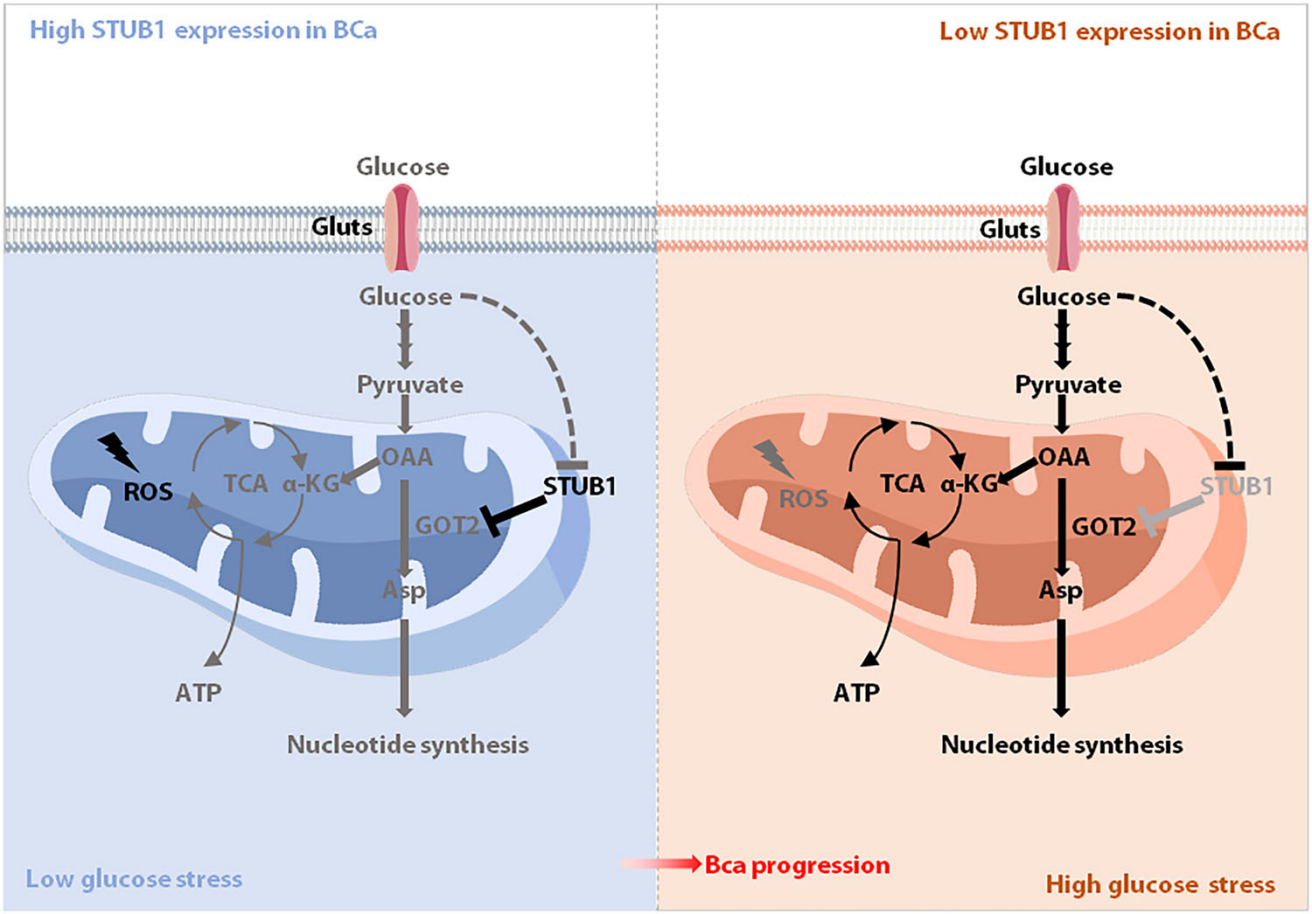
Schematic diagram of the STUB1-GOT2 axis in regulation of BCa progression (Created with figdraw.com.).

Previous studies suggest that STUB1 was response to oxidative stress [30, 31] and glucose stimulation [32, 33]. We next explore which upstream stress regulates STUB1-GOT2 signal axis. Interestingly, we determined that high glucose decreased STUB1 expression and upregulated GOT2 expression in both BCa cells (Fig. 7A, B). High glucose stimulation increased GOT2 expression (Fig. 7C, D), Asp synthesis (Fig. 7E, F), ATP synthesis (Fig. 7G, H), mitochondrial membrane potential (Fig. 7I and Supplementary Fig. S6A) and reduced ROS production (Fig. 7J and Supplementary Fig. S6B), whereas STUB1 overexpression can significantly rescued the effects in both T24 and UC3 cells. Moreover, high glucose stimulation could increase the stability of GOT2 (Fig. 7K) and significantly reduced polyubiquitination levels with GOT2, whereas STUB1 overexpression can significantly rescued this effect (Fig. 7L, M) (Supplementary Fig. S6C, D). These data demonstrated that high glucose promotes mitochondrial Asp synthesis and regulates mitochondrial dysfunction through STUB1-GOT2 signal axis. To examine the effects of high glucose induced STUB1-GOT2 axis in vivo, we used high glucose feed to feed tumor bearing mice. We found that STUB1 overexpression dramatically decreased tumor volumes (Fig. 7N), tumor sizes (Fig. 7O) and tumor weight (Fig. 7P), whereas high glucose feed can significantly rescued these effects. Additionally, the expression of STUB1 and GOT2 was further confirmed in a subcutaneous mouse xenograft model of T24 cells described as in Materials and methods (Fig. 7Q) and tumor cell proliferation measured by Ki67 was strongly reduced by STUB1 overexpression, whereas high glucose feed can significantly rescued this effect (Fig. 7R). Taken together, these results exemplify that glucose promotes Asp synthesis and BCa progression through STUB1-GOT2 axis (Fig. 8).

## Discussion

STUB1 regulates a variety of physiological processes including the occurrence and development of tumors by degrading its substrate protein [15, 20, 34]. In this study, STUB1 is highly enriched in the bladder tissue, reflecting a tissue-specific biological function. Low STUB1 expression is associated with a poor prognosis and STUB1 overexpression suppressed BCa cell growth in vitro and vivo, suggesting that STUB1 was a tumor suppressor. Amino acids perform critical metabolic functions. They are used for the synthesis of proteins, the formation of other low-molecular-weight compounds, and energy production by intermediate metabolites fueling other biosynthetic pathways [35, 36]. Herein, we discovered that STUB1 overexpression increased 18 amino acid metabolites and decreased 7 amino acid metabolites, especial Asp. These mean that STUB1 is critical for amino acid metabolic functions in BCa.

Mitochondrial glutamate-oxaloacetate transaminase 2 (GOT2) is part of the malate-aspartate shuttle, a mechanism by which cells transfer reducing equivalents from the cytosol to the mitochondria [25]. GOT2 is a key component of rewiring of glutamine metabolism in various cancers [37–39]. There are no reports on the regulatory mechanisms of GOT2 in BCa and the mechanisms governing GOT2 protein stability in human cancers remain largely unknown. Thus, the identification of the signaling pathway controlling GOT2 stabilization will be important to demonstrate GOT2 biology function and can be exploited for potential therapeutic interventions. Herein, by performing tandem affinity purification, we identified that the GOT2 binding protein, STUB1, functions as an E3 ligase, negatively regulates GOT2 stability through ubiquitin GOT2 at Lys 73 by promoting its K6/K48-linked ubiquitination. Thus, our study identified a key upstream effector which control GOT2 stabilization. Moreover, we observed that low expression of STUB1 was negatively correlated with high GOT2 expression in most human BCa tumors and STUB1 overexpression or GOT2 knockdown inhibited tumor growth, while co-overexpression of STUB1 and GOT2 knockdown enhance this effect, demonstrating a dominant role of STUB1-GOT2 axis in controlling of in vivo tumor growth. Our findings related to the STUB1/GOT2 complex are important as this mechanism may represent a general chaperone-ubiquitin-proteasome mechanism for the regulation of GOT2 protein stability that may be a potential therapeutic strategy in treating BCa.

The biosynthesis of aspartate is not only essential for protein synthesis but also for nucleotide biosynthesis in proliferating cells [26, 40, 41]. Asp biosynthesis is driven largely by glucose- or glutamine-dependent refilling of the tricarboxylic acid (TCA) cycle to replenish mitochondrial TCA intermediate oxaloacetate, which is subsequently converted to aspartate through the activity of GOT2 [11]. However, the intracellular signals that rewire aspartate biosynthesis to attenuate cell growth remain largely unknown. In this study, we provide evidence that STUB1 acts as a suppressor of tumor cell proliferation by controlling aspartate biosynthetic pathways through GOT2, not GOT1. The mitochondrial membrane potential is the key bio-energetic indicator that regulates respiration, ATP synthesis and ROS production, as well as the driving force for ion (beyond H+) and protein transport, which are essential for healthy mitochondria [27, 42]. Recent study reported that GOT2 knockdown reduced mitochondrial membrane potential. Here, we revealed that STUB1-GOT2 axis promoted ATP synthesis and ROS production through regulating mitochondrial dysfunction. The supportive evidence is as follows: (i) STUB1 overexpression or GOT2 suppression decreased ATP synthesis, whereas GOT2 overexpression can significantly rescued the effect of STUB1 overexpression in BCa cells. A similar result was observed in detecting ROS production. (ii) STUB1 overexpression or GOT2 suppression decreased mitochondrial membrane potential, whereas GOT2 overexpression can significantly rescued the effect of STUB1 overexpression in in BCa cells. These data show that STUB1-GOT2 pathway couples cellular oxygen supply and aspartate biosynthesis. Notably, hypoxia inducible factor-1α (HIF1α) acts as a direct repressor of aspartate biosynthesis to suppress several key aspartate-producing proteins, including mitochondrial GOT2 [11] and STUB1 reportedly interact with HIF-1α and promotes degradation of HIF-1α [43]. Further study will be needed to understand whether STUB1-HIF1α-GOT2 pathway couples cellular oxygen supply and aspartate biosynthesis.

Aberrant glucose metabolism is one of the characteristics of malignant tumors [44]. Large cohort studies have demonstrated that high glucose metabolism is crucial for the tumorigenesis of various solid tumors including BCa. Lattermann et al. found that the plasma concentration of glucose was elevated in patients with bladder cancer, which contributes to the development and progression of bladder cancer [5, 6]. Here, we accidentally found that high glucose stimulation increased GOT2 expression, Asp synthesis, ATP synthesis, mitochondrial membrane potential and reduced ROS production, whereas STUB1 overexpression can significantly rescued these effects. These findings explain why STUB1 was always downregulated in many tumors. High glucose metabolism in cancers may be the major upstream stress for maintaining the low expression of STUB1. Notably, we identified a novel STUB1-GOT2 axis, to clarify the relationship between STUB1 and glucose metabolism in bladder cancer cells.

In summary, our findings demonstrate that STUB1 is expressed at low levels in BCa. Downregulation of STUB1 is positively correlated with the outcome of patients with BCa. We also reveal that STUB1 negatively regulates GOT2 stability by promoting its K6/K48-linked ubiquitination, which promotes mitochondrial Asp synthesis and regulates mitochondrial dysfunction. Moreover, we identified that high glucose promotes Asp synthesis and tumor growth through STUB1-GOT2 axis. This study provides new insight regarding the pathogenesis of BCa under hyperglycemic conditions and might reveal ideal candidates by targeting STUB1-GOT2 axis for the development of drugs for BCa.

## Supplementary information

Supplemental Figures, Tables, Methods and References

original western blots

## Data Availability

The datasets used and/or analyzed during the current study are available from the corresponding author on reasonable request.
