# Supplementary material for: Glucose-induced STUB1-GOT2 axis promotes aspartate synthesis and mitochondrial dysfunction in bladder cancer
Source: Cell Death Dis. 2025 Jul 12;16(1):516. doi: 10.1038/s41419-025-07840-5 (PMC12255758; doi:10.1038/s41419-025-07840-5)
Supplement: Supplementary file 1 — Supplemental Figures, Tables, Methods and References [file 41419_2025_7840_MOESM1_ESM.doc]

**Supplementary Materials**

**Supplemental Figures, Tables, Methods and References**

**Supplementary Figures**

**Fig. S1**

**
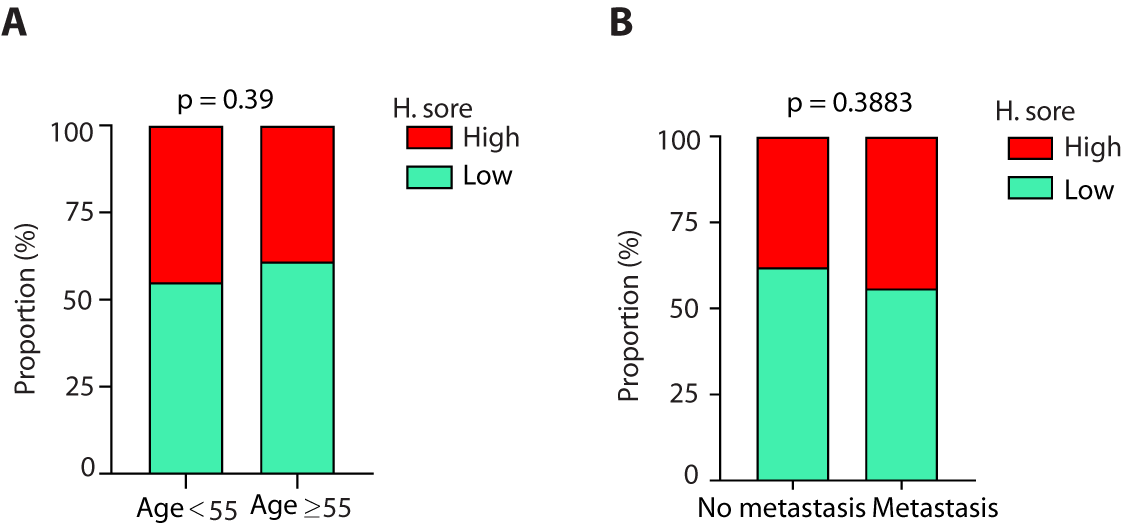
**

**Fig. S1**

**A, B** A low STUB1 expression was not related with age (*p* = 0.39) (**A**) and a lymph node metastasis (*p* = 0.3883) (**B**).

**Fig. S2**

**
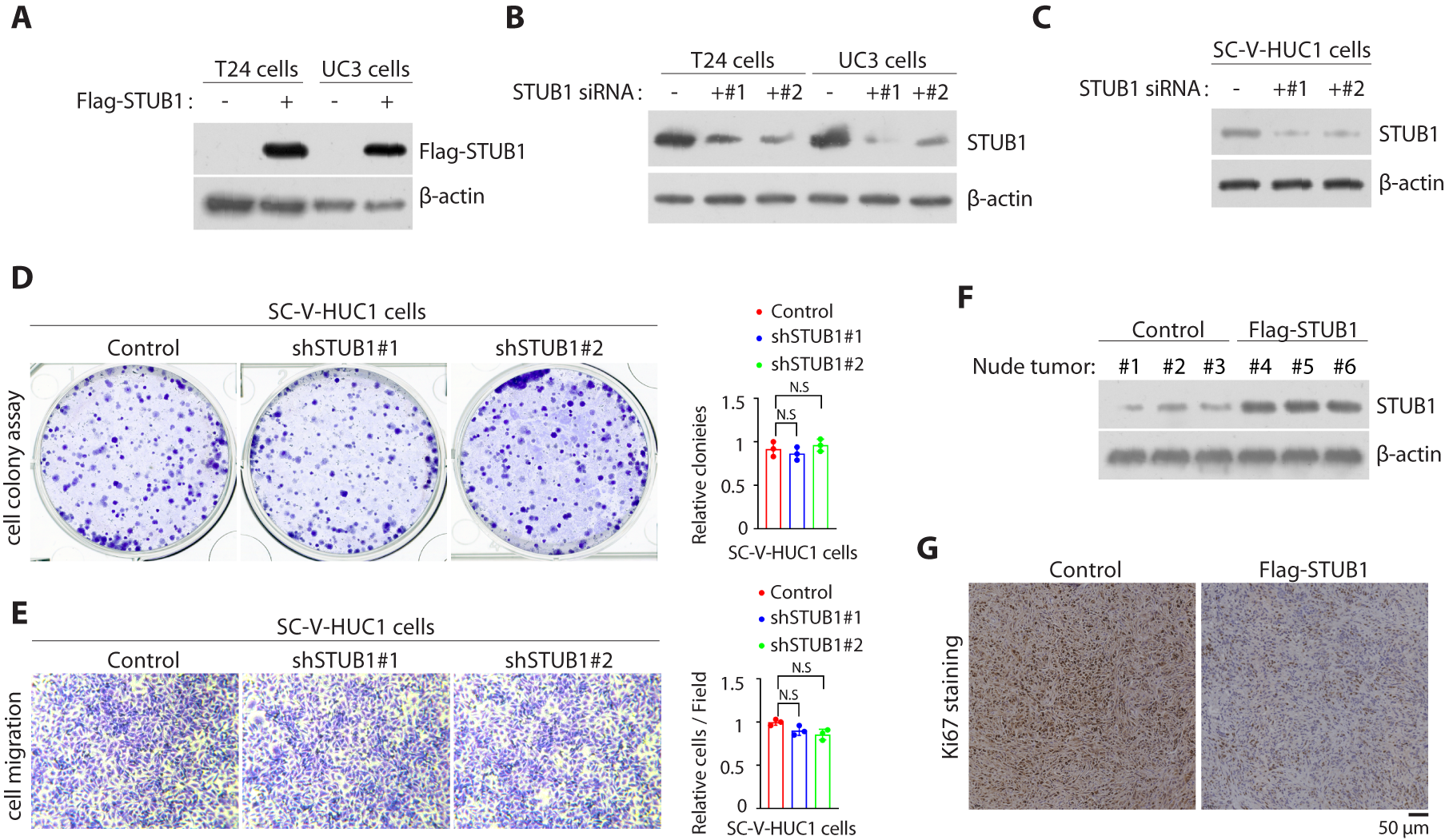
**

**Fig. S2**

**A, B** Overexpression (**A**) or knockdown (**B**) of STUB1 in T24 or UC3 cells was analyzed by Western Blot. **C**-E Knockdown of STUB1 in SC-V-HUC1 cells was analyzed by Western Blot (**C**) and cells were performed by colony formation assays (**D**) and the motility was examined by migration assay (**E**). **F** The tumor tissues from the indicated nude mice (each group n = 3) were used to detect STUB1 protein expression. **G** Ki-67 staining was performed in the tumor tissues (each group n = 1) from the indicated nude mice.

**Fig. S3**


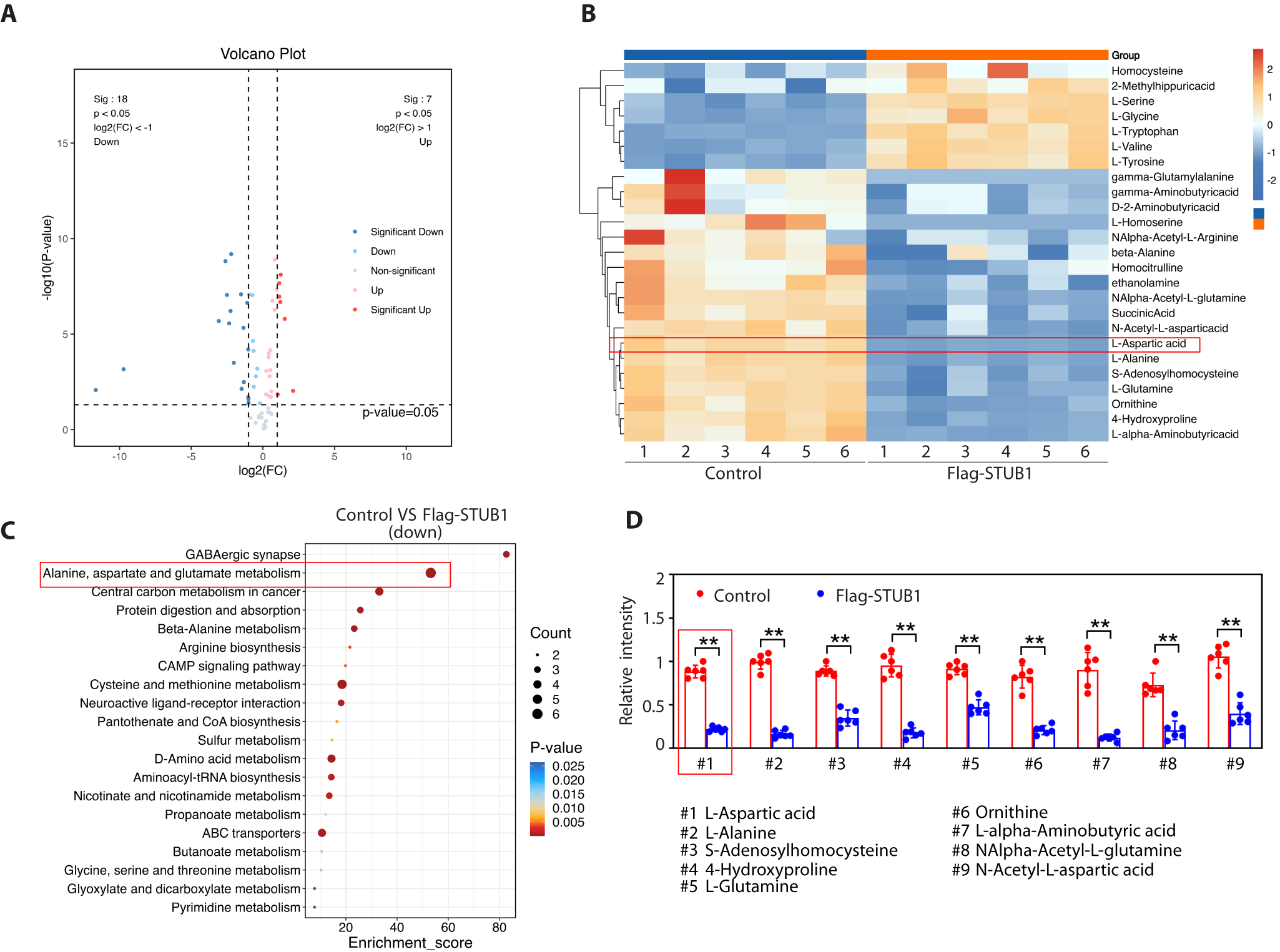


**Fig. S3**

**A-C** Volcano analysis (**A**), Heatmap analysis (**B**), KEGG pathway (**C**). **D** Bar chart was used to cluster of all amino acids altering more than 2-fold down-regulation upon STUB1 overexpression in T24 cells by using metabolomics sequencing assay. The *p* values were obtained by two-tailed unpaired t test. ^*^*p* < 0.05, ^**^*p* < 0.01.

**Fig. S4**

**
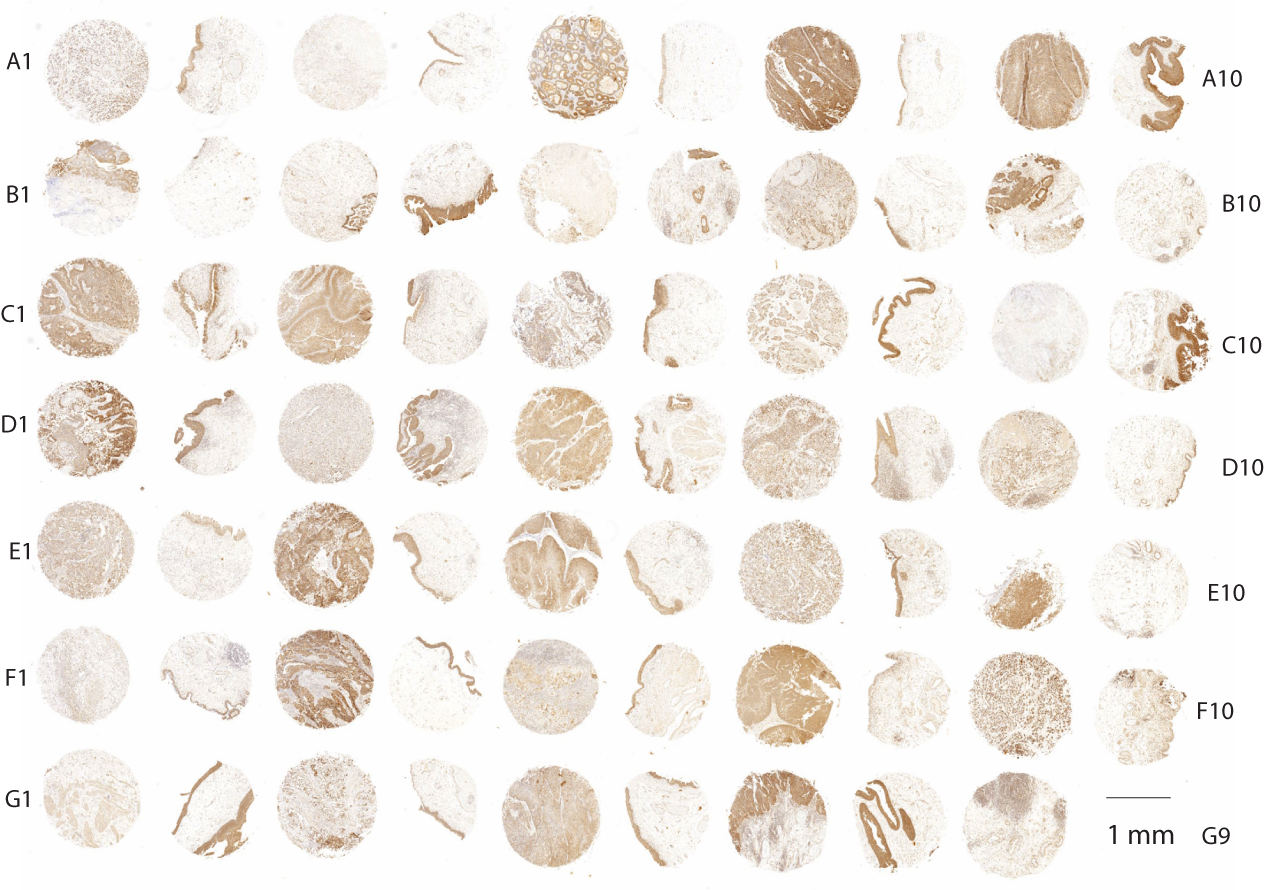
**

**Fig. S4**

Immunohistochemical staining of GOT2 tissue microarray containing 35 samples of PCa tissues and matched 34 samples of adjacent non-tumor tissues.

**Fig. S5**

**
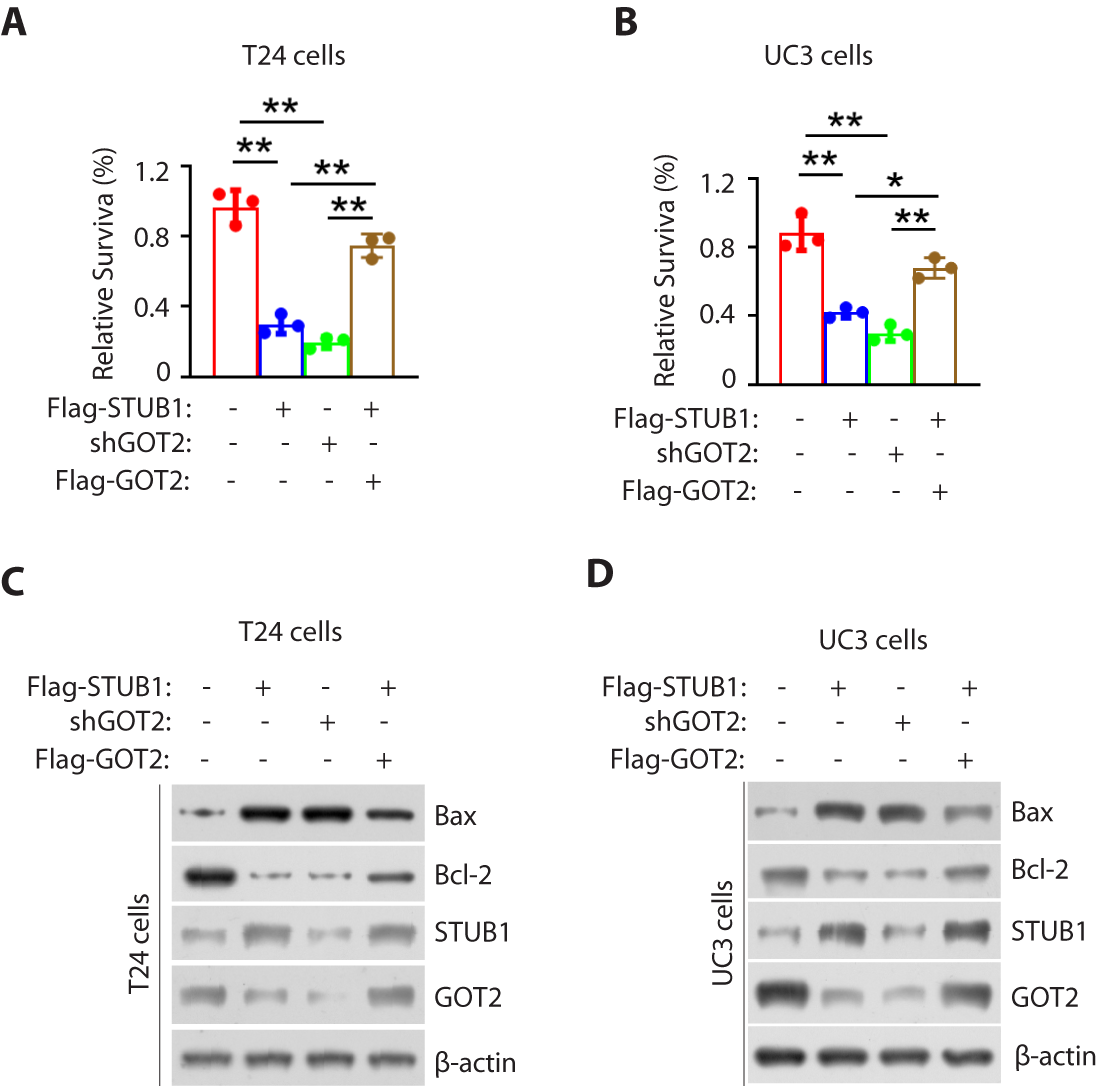
**

**Fig. S5**

**A, B** The ability of the proliferation of the indicated T24 cells and UC3 cells were performed by CCK8 assay. **C, D** The indicated T24 cells and UC3 cells were analyzed by Western Blot to detect the marker protein of caspase (Bax and Bcl-2).

**Fig. S6**

**
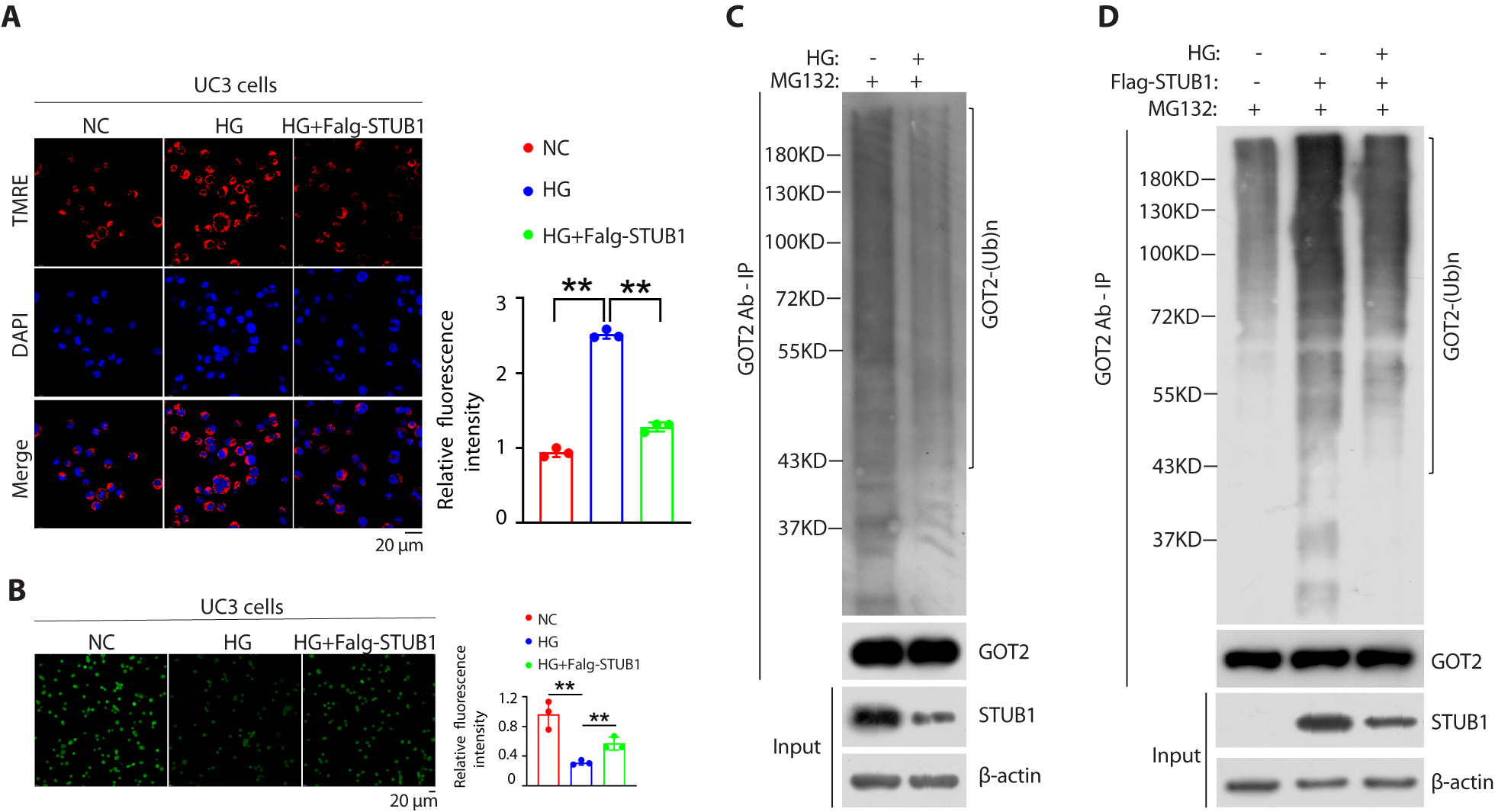
**

**Fig. S6**

**A** Immunofluorescent staining of mitochondrial membrane potential (red) with mitochondrial membrane potential assay Kit and nuclear (blue) with Nuclear 4', 6-diamidino-2-phenylindole in the UC3 cells with or without overexpressing STUB1 after stimulation with 4.5g/L glucose for 6 hr in serum free medium. **B** Immunofluorescent staining of reactive oxygen species (blue) with ROS Assay Kit in the UC3 cells described as **Fig. 7I. C** UC3 cells were described and analyzed as **Fig. 7L**. **D** UC3 cells were described and analyzed as

**Supplementary Tables**

**Table S1**

| location | number | age | gender | organ | pathology | TNM | Grade | Stage | Type | STUB1-SCORE |
| --- | --- | --- | --- | --- | --- | --- | --- | --- | --- | --- |
| A1 | 1 | 70 | male | bladder | urothelial carcinoma | T2bN1M0 | 3 | IIIA | tumor | 24.12 |
| A2 | 2 | 70 | male | bladder | urothelial carcinoma | T2bN1M0 | 3 | IIIA | tumor | 13.79 |
| A3 | 3 | 85 | male | bladder | urothelial carcinoma | T2aN0M0 | 3 | II | tumor | 5.816 |
| A4 | 4 | 85 | male | bladder | urothelial carcinoma | T2aN0M0 | 3 | II | tumor | 26.24 |
| A5 | 5 | 75 | male | bladder | urothelial carcinoma | T2N1M0 | 3 | IIIA | tumor | 6.487 |
| A6 | 6 | 75 | male | bladder | urothelial carcinoma | T2N1M0 | 3 | IIIA | tumor | 5.858 |
| A7 | 7 | 73 | male | bladder | urothelial carcinoma | T2N0M0 | 3 | II | tumor | 124.5 |
| A8 | 8 | 73 | male | bladder | urothelial carcinoma | T2N0M0 | 3 | II | tumor | 106.7 |
| A9 | 9 | 45 | female | bladder | urothelial carcinoma | T2N0M0 | 3 | II | tumor | 6.553 |
| A10 | 10 | 45 | female | bladder | urothelial carcinoma | T2N0M0 | 3 | II | tumor | 6.962 |
| A11 | 11 | 61 | male | bladder | urothelial carcinoma | T2N0M0 | 3 | II | tumor | 43.95 |
| A12 | 12 | 61 | male | bladder | urothelial carcinoma | T2N0M0 | 3 | II | tumor | 45.18 |
| A13 | 13 | 58 | male | bladder | urothelial carcinoma | T2N0M0 | 3 | II | tumor | 45.12 |
| A14 | 14 | 58 | male | bladder | urothelial carcinoma | T2N0M0 | 3 | II | tumor | 36.81 |
| A15 | 15 | 63 | male | bladder | urothelial carcinoma | T1N0M0 | 1 | I | tumor | 8.057 |
| A16 | 16 | 63 | male | bladder | urothelial carcinoma | T1N0M0 | 1 | I | tumor | 2.485 |
| B1 | 17 | 81 | male | bladder | urothelial carcinoma | T4aN0M0 | 3 | IIIA | tumor | 3.901 |
| B2 | 18 | 81 | male | bladder | urothelial carcinoma | T4aN0M0 | 3 | IIIA | tumor | 4.099 |
| B3 | 19 | 82 | male | bladder | urothelial carcinoma | T2N0M0 | 3 | II | tumor | 32.54 |
| B4 | 20 | 82 | male | bladder | urothelial carcinoma | T2N0M0 | 3 | II | tumor | 36.27 |
| B5 | 21 | 46 | female | bladder | urothelial carcinoma | T2N0M0 | 3 | II | tumor | 30.63 |
| B6 | 22 | 46 | female | bladder | urothelial carcinoma | T2N0M0 | 3 | II | tumor | 55.05 |
| B7 | 23 | 84 | male | bladder | urothelial carcinoma | T2N0M0 | 3 | II | tumor | 2.954 |
| B8 | 24 | 84 | male | bladder | urothelial carcinoma | T2N0M0 | 3 | II | tumor | 4.495 |
| B9 | 25 | 62 | male | bladder | urothelial carcinoma | T3aN0M0 | 3 | IIIA | tumor | 16.59 |
| B10 | 26 | 62 | male | bladder | urothelial carcinoma | T3aN0M0 | 3 | IIIA | tumor | 33.3 |
| B11 | 27 | 65 | male | bladder | urothelial carcinoma | T2bN0M0 | 1 | II | tumor | 12.2 |
| B12 | 28 | 65 | male | bladder | urothelial carcinoma | T2bN0M0 | 3 | II | tumor | 6.715 |
| B13 | 29 | 65 | female | bladder | urothelial carcinoma | T1N0M0 | 3 | I | tumor | 29.51 |
| B14 | 30 | 65 | female | bladder | urothelial carcinoma | T1N0M0 | 3 | I | tumor | 23.92 |
| B15 | 31 | 53 | male | bladder | urothelial carcinoma | T2aN0M0 | 3 | II | tumor | 16.17 |
| B16 | 32 | 53 | male | bladder | urothelial carcinoma | T2aN0M0 | 3 | II | tumor | 12.97 |
| C1 | 33 | 74 | female | bladder | urothelial carcinoma | T1N0M0 | 3 | I | tumor | 57.53 |
| C2 | 34 | 74 | female | bladder | urothelial carcinoma | T1N0M0 | 3 | I | tumor | 45.25 |
| C3 | 35 | 46 | male | bladder | urothelial carcinoma | T2aN0M0 | 3 | II | tumor | 56.97 |
| C4 | 36 | 46 | male | bladder | urothelial carcinoma | T2aN0M0 | 3 | II | tumor | 102.2 |
| C5 | 37 | 52 | male | bladder | urothelial carcinoma | T2aN0M0 | 3 | II | tumor | 109 |
| C6 | 38 | 52 | male | bladder | urothelial carcinoma | T2aN0M0 | 3 | II | tumor | 66.22 |
| C7 | 39 | 92 | male | bladder | urothelial carcinoma | T2N0M0 | 3 | II | tumor | 68.53 |
| C8 | 40 | 92 | male | bladder | urothelial carcinoma | T2N0M0 | 3 | II | tumor | 90.78 |
| C9 | 41 | 46 | male | bladder | urothelial carcinoma | T2aN0M0 | 3 | II | tumor | 32.7 |
| C10 | 42 | 46 | male | bladder | urothelial carcinoma | T2aN0M0 | 3 | II | tumor | 68.33 |
| C11 | 43 | 90 | male | bladder | urothelial carcinoma | T3N0M0 | 3 | IIIA | tumor | 9.569 |
| C12 | 44 | 90 | male | bladder | urothelial carcinoma | T3N0M0 | 3 | IIIA | tumor | 17.58 |
| C13 | 45 | 49 | female | bladder | urothelial carcinoma | T2bN0M0 | 2 | II | tumor | 19.19 |
| C14 | 46 | 49 | female | bladder | urothelial carcinoma | T2bN0M0 | 2 | II | tumor | 29.54 |
| D1 | 47 | 64 | male | bladder | urothelial carcinoma | T2bN0M0 | 3 | II | tumor | 30.57 |
| D2 | 48 | 64 | male | bladder | urothelial carcinoma | T2bN0M0 | 3 | II | tumor | 8.643 |
| D3 | 49 | 68 | male | bladder | urothelial carcinoma | T3aN0M0 | 3 | IIIA | tumor | 1.578 |
| D4 | 50 | 68 | male | bladder | urothelial carcinoma | T3aN0M0 | 3 | IIIA | tumor | 5.411 |
| D5 | 51 | 81 | male | bladder | urothelial carcinoma | T2bN0M0 | 3 | II | tumor | 83.19 |
| D6 | 52 | 81 | male | bladder | urothelial carcinoma | T2bN0M0 | 3 | II | tumor | 100.7 |
| D7 | 53 | 42 | female | bladder | urothelial carcinoma | T1N0M0 | 3 | I | tumor | 55.69 |
| D8 | 54 | 42 | female | bladder | urothelial carcinoma | T1N0M0 | 3 | I | tumor | 77.31 |
| D9 | 55 | 78 | male | bladder | urothelial carcinoma | T2N0M0 | 3 | II | tumor | 6.291 |
| D10 | 56 | 78 | male | bladder | urothelial carcinoma | T2N0M0 | 3 | II | tumor | 23.08 |
| D11 | 57 | 55 | male | bladder | urothelial carcinoma | T2aN0M0 | 3 | II | tumor | 48.04 |
| D12 | 58 | 55 | male | bladder | urothelial carcinoma | T2aN0M0 | 3 | II | tumor | 45.48 |
| D13 | 59 | 39 | female | bladder | urothelial carcinoma | T2aN0M0 | 3 | II | tumor | 39.55 |
| D14 | 60 | 39 | female | bladder | urothelial carcinoma | T2aN0M0 | 3 | II | tumor | 14.71 |
| E1 | 61 | 76 | male | bladder | urothelial carcinoma | T2bN0M0 | 3 | II | tumor | 16.07 |
| E2 | 62 | 76 | male | bladder | urothelial carcinoma | T2bN0M0 | 3 | II | tumor | 25.4 |
| E3 | 63 | 69 | male | bladder | urothelial carcinoma | T4aN1M0 | 3 | IIIA | tumor | 25.49 |
| E4 | 64 | 69 | male | bladder | urothelial carcinoma | T4aN1M0 | 3 | IIIA | tumor | 11.11 |
| E5 | 65 | 61 | female | bladder | urothelial carcinoma | T2bN1M0 | 3 | IIIA | tumor | 37.56 |
| E6 | 66 | 61 | female | bladder | urothelial carcinoma | T2bN1M0 | 3 | IIIA | tumor | 60.25 |
| E7 | 67 | 82 | male | bladder | urothelial carcinoma | T2bN0M0 | 3 | II | tumor | 2.435 |
| E8 | 68 | 82 | male | bladder | urothelial carcinoma | T2bN0M0 | 3 | II | tumor | 1.644 |
| E9 | 69 | 81 | male | bladder | urothelial carcinoma | T2bN0M0 | 3 | II | tumor | 47.43 |
| E10 | 70 | 81 | male | bladder | urothelial carcinoma | T2bN0M0 | 3 | II | tumor | 43.01 |
| E11 | 71 | 84 | male | bladder | urothelial carcinoma | T3N0M0 | 3 | IIIA | tumor | 36.73 |
| E12 | 72 | 84 | male | bladder | urothelial carcinoma | T3N0M0 | 3 | IIIA | tumor | 36.17 |
| E13 | 73 | 68 | male | bladder | urothelial carcinoma | T2bN0M0 | 3 | II | tumor | 2.33 |
| E14 | 74 | 68 | male | bladder | urothelial carcinoma | T2bN0M0 | 3 | II | tumor | 1.299 |
| F1 | 75 | 68 | male | bladder | urothelial carcinoma | T3bN0M0 | 3 | IIIA | tumor | 26.48 |
| F2 | 76 | 68 | male | bladder | urothelial carcinoma | T3bN0M0 | 3 | IIIA | tumor | 38.37 |
| F3 | 77 | 73 | male | bladder | urothelial carcinoma | T2bN0M0 | 3 | II | tumor | 1.41 |
| F4 | 78 | 73 | male | bladder | urothelial carcinoma | T2bN0M0 | 3 | II | tumor | 1.366 |
| F5 | 79 | 69 | male | bladder | urothelial carcinoma | T4aN0M0 | 3 | IIIA | tumor | 6.544 |
| F6 | 80 | 69 | male | bladder | urothelial carcinoma | T4aN0M0 | 3 | IIIA | tumor | 7.8 |
| F7 | 81 | 73 | female | bladder | urothelial carcinoma | T2N0M0 | 3 | II | tumor | 1.262 |
| F8 | 82 | 73 | female | bladder | urothelial carcinoma | T2N0M0 | 3 | II | tumor | 3.173 |
| F9 | 83 | 67 | female | bladder | urothelial carcinoma | T3bN0M0 | 3 | IIIA | tumor | 3.367 |
| F10 | 84 | 67 | female | bladder | urothelial carcinoma | T3bN0M0 | 3 | IIIA | tumor | 23.81 |
| F11 | 85 | 68 | male | bladder | urothelial carcinoma | T2N0M0 | 3 | II | tumor | 1.441 |
| F12 | 86 | 68 | male | bladder | urothelial carcinoma | T2N0M0 | 3 | II | tumor | 2.429 |
| F13 | 87 | 69 | male | bladder | urothelial carcinoma | T2bN0M0 | 3 | II | tumor | 9.46 |
| F14 | 88 | 69 | male | bladder | urothelial carcinoma | T2bN0M0 | 3 | II | tumor | 14.8 |
| G1 | 89 | 54 | male | bladder | urothelial carcinoma | T2bN0M0 | 3 | II | tumor | 2.082 |
| G2 | 90 | 54 | male | bladder | urothelial carcinoma | T2bN0M0 | 3 | II | tumor | 1.824 |
| G3 | 91 | 78 | female | bladder | urothelial carcinoma | T2bN0M0 | 3 | II | tumor | 19.97 |
| G4 | 92 | 78 | female | bladder | urothelial carcinoma | T2bN0M0 | 3 | II | tumor | 9.517 |
| G5 | 93 | 78 | male | bladder | urothelial carcinoma | T2aN0M0 | 3 | II | tumor | 3.822 |
| G6 | 94 | 78 | male | bladder | urothelial carcinoma | T2aN0M0 | 3 | II | tumor | 3.217 |
| G7 | 95 | 91 | male | bladder | urothelial carcinoma | T4aN1M0 | 3 | IIIA | tumor | 5.001 |
| G8 | 96 | 91 | male | bladder | urothelial carcinoma | T4aN1M0 | 3 | IIIA | tumor | 4.777 |
| G9 | 97 | 70 | male | bladder | urothelial carcinoma | T1N0M0 | 1 | I | tumor | 24.84 |
| G10 | 98 | 70 | male | bladder | urothelial carcinoma | T1N0M0 | 1 | I | tumor | 31.84 |
| G11 | 99 | 59 | male | bladder | urothelial carcinoma | T4aN1M0 | 3 | IIIA | tumor | 20.06 |
| G12 | 100 | 59 | male | bladder | urothelial carcinoma | T4aN1M0 | 3 | IIIA | tumor | 23.78 |
| G13 | 101 | 61 | male | bladder | urothelial carcinoma | T2aN1M0 | 3 | IIIA | tumor | 1.161 |
| G14 | 102 | 61 | male | bladder | urothelial carcinoma | T2aN1M0 | 3 | IIIA | tumor | 3.83 |
| H1 | 103 | 49 | male | bladder | urothelial carcinoma | T2bN1M0 | 2 | IIIA | tumor | 3.844 |
| H2 | 104 | 49 | male | bladder | urothelial carcinoma | T2bN1M0 | 2 | IIIA | tumor | 4.539 |
| H3 | 105 | 51 | male | bladder | urothelial carcinoma | T2bN0M0 | 2 | II | tumor | 2.539 |
| H4 | 106 | 51 | male | bladder | urothelial carcinoma | T2bN0M0 | 2 | II | tumor | 1.357 |
| H5 | 107 | 69 | female | bladder | urothelial carcinoma | T2aN0M0 | 1 | II | tumor | 1.366 |
| H6 | 108 | 69 | female | bladder | urothelial carcinoma | T2aN0M0 | 1 | II | tumor | 1.441 |
| H7 | 109 | 85 | female | bladder | urothelial carcinoma | T2bN0M0 | 2 | II | tumor | 5.117 |
| H8 | 110 | 85 | female | bladder | urothelial carcinoma | T2bN0M0 | 2 | II | tumor | 4.813 |
| H9 | 111 | 81 | male | bladder | urothelial carcinoma | T2bN0M0 | 3 | II | tumor | 16.05 |
| H10 | 112 | 81 | male | bladder | urothelial carcinoma | T2bN0M0 | 3 | II | tumor | 43.06 |
| H11 | 113 | 69 | male | bladder | urothelial carcinoma | T2aN0M0 | 3 | II | tumor | 19.61 |
| H12 | 114 | 69 | male | bladder | urothelial carcinoma | T2aN0M0 | 3 | II | tumor | 28.55 |
| H13 | 115 | 55 | male | bladder | urothelial carcinoma | T2bN0M0 | 3 | II | tumor | 27.89 |
| H14 | 116 | 55 | male | bladder | urothelial carcinoma | T2bN0M0 | 3 | II | tumor | 29.34 |
| I1 | 117 | 62 | female | bladder | urothelial carcinoma | T2bN0M0 | 3 | II | tumor | 11.74 |
| I2 | 118 | 62 | female | bladder | urothelial carcinoma | T2bN0M0 | 3 | II | tumor | 9.292 |
| I3 | 119 | 55 | male | bladder | urothelial carcinoma | T2bN0M0 | 3 | II | tumor | 33.36 |
| I4 | 120 | 55 | male | bladder | urothelial carcinoma | T2bN0M0 | 3 | II | tumor | 15.63 |
| I5 | 121 | 59 | female | bladder | urothelial carcinoma | T2bN0M0 | 3 | II | tumor | 43.73 |
| I6 | 122 | 59 | female | bladder | urothelial carcinoma | T2bN0M0 | 3 | II | tumor | 33.88 |
| I7 | 123 | 60 | male | bladder | urothelial carcinoma | T1N0M0 | 1 | I | tumor | 46.81 |
| I8 | 124 | 60 | male | bladder | urothelial carcinoma | T1N0M0 | 1 | I | tumor | 45.63 |
| I9 | 125 | 70 | female | bladder | urothelial carcinoma | T2bN0M0 | 3 | II | tumor | 5.603 |
| I10 | 126 | 70 | female | bladder | urothelial carcinoma | T2bN0M0 | 3 | II | tumor | 11.84 |
| I11 | 127 | 74 | male | bladder | urothelial carcinoma | T4aN0M0 | 3 | IIIA | tumor | 30.54 |
| I12 | 128 | 74 | male | bladder | urothelial carcinoma | T4aN0M0 | 3 | IIIA | tumor | 46.78 |
| I13 | 129 | 64 | male | bladder | urothelial carcinoma | T2bN2M0 | 3 | IIIB | tumor | 18.37 |
| I14 | 130 | 64 | male | bladder | urothelial carcinoma | T2bN2M0 | 3 | IIIB | tumor | 21.37 |
| J1 | 131 | 49 | male | bladder | urothelial carcinoma | T1N0M0 | 3 | I | tumor | 2.441 |
| J2 | 132 | 49 | male | bladder | urothelial carcinoma | T1N0M0 | 3 | I | tumor | 2.871 |
| J3 | 133 | 60 | male | bladder | urothelial carcinoma | T1N0M0 | 1 | I | tumor | 61.61 |
| J4 | 134 | 60 | male | bladder | urothelial carcinoma | T1N0M0 | 1 | I | tumor | 49.06 |
| J5 | 135 | 63 | male | bladder | urothelial carcinoma | T2bN0M0 | 3 | II | tumor | 44.31 |
| J6 | 136 | 63 | male | bladder | urothelial carcinoma | T2bN0M0 | 3 | II | tumor | 31.66 |
| J7 | 137 | 55 | female | bladder | urothelial carcinoma | T2bN0M0 | 3 | II | tumor | 9.129 |
| J8 | 138 | 55 | female | bladder | urothelial carcinoma | T2bN0M0 | 3 | II | tumor | 8.314 |
| J9 | 139 | 67 | male | bladder | urothelial carcinoma | T2aN0M0 | 3 | II | tumor | 90.45 |
| J10 | 140 | 67 | male | bladder | urothelial carcinoma | T2aN0M0 | 3 | II | tumor | 78.76 |
| J11 | 141 | 82 | male | bladder | urothelial carcinoma | T2aN0M0 | 3 | II | tumor | 11.12 |
| J12 | 142 | 82 | male | bladder | urothelial carcinoma | T2aN0M0 | 3 | II | tumor | 14.81 |
| J13 | 143 | 63 | male | bladder | urothelial carcinoma | T2aN0M0 | 3 | II | tumor | 18.6 |
| J14 | 144 | 63 | male | bladder | urothelial carcinoma | T2aN0M0 | 3 | II | tumor | 37 |
| K1 | 145 | 71 | female | bladder | urothelial carcinoma | T2bN0M0 | 3 | II | tumor | 8.798 |
| K2 | 146 | 71 | female | bladder | urothelial carcinoma | T2bN0M0 | 3 | II | tumor | 9.473 |
| K3 | 147 | 72 | male | bladder | urothelial carcinoma | T2aN0M0 | 3 | II | tumor | 68.93 |
| K4 | 148 | 72 | male | bladder | urothelial carcinoma | T2aN0M0 | 3 | II | tumor | 52.07 |
| K5 | 149 | 71 | male | bladder | urothelial carcinoma | T2bN0M0 | 3 | II | tumor | 12.18 |
| K6 | 150 | 71 | male | bladder | urothelial carcinoma | T2bN0M0 | 3 | II | tumor | 23.03 |
| K7 | 151 | 57 | male | bladder | urothelial carcinoma | T2N0M0 | 3 | II | tumor | 1.917 |
| K8 | 152 | 57 | male | bladder | urothelial carcinoma | T2N0M0 | 3 | II | tumor | 2.935 |
| K9 | 153 | 79 | male | bladder | urothelial carcinoma | T3bN0M0 | 3 | IIIA | tumor | 3.562 |
| K10 | 154 | 79 | male | bladder | urothelial carcinoma | T3bN0M0 | 3 | IIIA | tumor | 2.691 |
| K11 | 155 | 49 | male | bladder | urothelial carcinoma | T2N0M0 | 3 | II | tumor | 78.41 |
| K12 | 156 | 49 | male | bladder | urothelial carcinoma | T2N0M0 | 3 | II | tumor | 62.36 |
| K13 | 157 | 78 | male | bladder | urothelial carcinoma | T2bN0M0 | 3 | II | tumor | 4.352 |
| K14 | 158 | 78 | male | bladder | urothelial carcinoma | T2bN0M0 | 3 | II | tumor | 8.804 |
| L1 | 159 | 65 | male | bladder | urothelial carcinoma | T3bN0M0 | 3 | IIIA | tumor | 42.9 |
| L2 | 160 | 65 | male | bladder | urothelial carcinoma | T3bN0M0 | 3 | IIIA | tumor | 11.79 |
| L3 | 161 | 67 | male | bladder | urothelial carcinoma | T2N1M0 | 3 | IIIA | tumor | 71.26 |
| L4 | 162 | 67 | male | bladder | urothelial carcinoma | T2N1M0 | 3 | IIIA | tumor | 12.84 |
| L5 | 163 | 70 | female | bladder | urothelial carcinoma | T2N0M0 | 3 | II | tumor | 47.66 |
| L6 | 164 | 70 | female | bladder | urothelial carcinoma | T2N0M0 | 3 | II | tumor | 37.51 |
| L7 | 165 | 63 | female | bladder | urothelial carcinoma | T1N0M0 | 3 | I | tumor | 39.57 |
| L8 | 166 | 63 | female | bladder | urothelial carcinoma | T1N0M0 | 3 | I | tumor | 55.71 |
| L9 | 167 | 65 | male | bladder | urothelial carcinoma | T2aN0M0 | 3 | II | tumor | 19.11 |
| L10 | 168 | 65 | male | bladder | urothelial carcinoma | T2aN0M0 | 3 | II | tumor | 12.69 |
| L11 | 169 | 63 | male | bladder | urothelial carcinoma | T2aN0M0 | 3 | II | tumor | 56.05 |
| L12 | 170 | 63 | male | bladder | urothelial carcinoma | T2aN0M0 | 3 | II | tumor | 81.33 |
| L13 | 171 | 48 | male | bladder | urothelial carcinoma | T2bN1M0 | 3 | IIIA | tumor | 42.99 |
| L14 | 172 | 48 | male | bladder | urothelial carcinoma | T2bN1M0 | 3 | IIIA | tumor | 8.617 |

**Table S2**

| location | number | age | gender | organ | pathology | TNM | Grade | Stage | Type | STUB1-SCORE |
| --- | --- | --- | --- | --- | --- | --- | --- | --- | --- | --- |
| A1 | 1 | 64 | male | bladder | urothelial carcinoma | T2bN0M0 | 3 | II | tumor | 34.179 |
| A2 | 2 | 64 | male | bladder | pericarcinomatous tissue | - | - | - | normal | 122.871 |
| A3 | 3 | 87 | male | bladder | urothelial carcinoma | T2aN0M0 | 1 | II | tumor | 30.295 |
| A4 | 4 | 87 | male | bladder | pericarcinomatous tissue | - | - | - | normal | 84.09 |
| A5 | 5 | 50 | male | bladder | urothelial carcinoma | T2aN0M0 | 1 | II | tumor | 117.785 |
| A6 | 6 | 50 | male | bladder | pericarcinomatous tissue | - | - | - | normal | 77.478 |
| A7 | 7 | 69 | male | bladder | urothelial carcinoma | T4N0M0 | 3 | IIIA | tumor | 7.909 |
| A8 | 8 | 69 | male | bladder | pericarcinomatous tissue | - | - | - | normal | 87.052 |
| A9 | 9 | 61 | male | bladder | urothelial carcinoma | T1N0M0 | 3 | I | tumor | 91.888 |
| A10 | 10 | 61 | male | bladder | pericarcinomatous tissue | - | - | - | normal | 118.614 |
| B1 | 11 | 54 | male | bladder | urothelial carcinoma | T2bN0M0 | 3 | II | tumor | 79.148 |
| B2 | 12 | 54 | male | bladder | pericarcinomatous tissue | - | - | - | normal | 53.922 |
| B3 | 13 | 64 | male | bladder | urothelial carcinoma | T1N0M0 | 1 | I | tumor | 85.415 |
| B4 | 14 | 64 | male | bladder | pericarcinomatous tissue | - | - | - | normal | 125.368 |
| B5 | 15 | 68 | male | bladder | urothelial carcinoma | T2bN0M0 | 3 | II | tumor | 79.906 |
| B6 | 16 | 68 | male | bladder | pericarcinomatous tissue | - | - | - | normal | 127.5 |
| B7 | 17 | 65 | male | bladder | urothelial carcinoma | T3aN0M0 | 3 | IIIA | tumor | 76.592 |
| B8 | 18 | 65 | male | bladder | pericarcinomatous tissue | - | - | - | normal | 80.435 |
| B9 | 19 | 64 | male | bladder | urothelial carcinoma | T3aN0M0 | 2 | IIIA | tumor | 96.452 |
| B10 | 20 | 64 | male | bladder | pericarcinomatous tissue | - | - | - | normal | 37.154 |
| C1 | 21 | 67 | male | bladder | urothelial carcinoma | T1N0M0 | 3 | I | tumor | 77.58 |
| C2 | 22 | 67 | male | bladder | pericarcinomatous tissue | - | - | - | normal | 92.576 |
| C3 | 23 | 52 | female | bladder | urothelial carcinoma | T2aN0M0 | 3 | II | tumor | 97.674 |
| C4 | 24 | 52 | female | bladder | pericarcinomatous tissue | - | - | - | normal | 106.611 |
| C5 | 25 | 76 | male | bladder | urothelial carcinoma | T2aN0M0 | 3 | II | tumor | 55.481 |
| C6 | 26 | 76 | male | bladder | pericarcinomatous tissue | - | - | - | normal | 115.914 |
| C7 | 27 | 70 | male | bladder | urothelial carcinoma | T4aN2M0 | 3 | IIIB | tumor | 111.219 |
| C8 | 28 | 70 | male | bladder | pericarcinomatous tissue | - | - | - | normal | 147.351 |
| C9 | 29 | 70 | male | bladder | urothelial carcinoma | T2aN0M0 | 3 | II | tumor | 16.379 |
| C10 | 30 | 70 | male | bladder | pericarcinomatous tissue | - | - | - | normal | 103.516 |
| D1 | 31 | 77 | female | bladder | urothelial carcinoma | T1N0M0 | 3 | I | tumor | 70.94 |
| D2 | 32 | 77 | female | bladder | pericarcinomatous tissue | - | - | - | normal | 120.464 |
| D3 | 33 | 41 | male | bladder | urothelial carcinoma | T3aN0M0 | 3 | III | tumor | 36.754 |
| D4 | 34 | 41 | male | bladder | pericarcinomatous tissue | - | - | - | normal | 129.055 |
| D5 | 35 | 92 | male | bladder | urothelial carcinoma | T4aN1M0 | 3 | IIIA | tumor | 100.437 |
| D6 | 36 | 92 | male | bladder | pericarcinomatous tissue | - | - | - | normal | 131.252 |
| D7 | 37 | 82 | male | bladder | urothelial carcinoma | T2aN0M0 | 3 | II | tumor | 24.618 |
| D8 | 38 | 82 | male | bladder | pericarcinomatous tissue | - | - | - | normal | 76.009 |
| D9 | 39 | 72 | male | bladder | urothelial carcinoma | T2bN1M0 | 3 | IIIA | tumor | 46.938 |
| D10 | 40 | 72 | male | bladder | pericarcinomatous tissue | - | - | - | normal | 61.125 |
| E1 | 41 | 60 | male | bladder | urothelial carcinoma | T2bN1M0 | 3 | IIIA | tumor | 71.725 |
| E2 | 42 | 60 | male | bladder | pericarcinomatous tissue | - | - | - | normal | 80.68 |
| E3 | 43 | 78 | male | bladder | urothelial carcinoma | T2bN0M0 | 3 | II | tumor | 7.356 |
| E4 | 44 | 78 | male | bladder | pericarcinomatous tissue | - | - | - | normal | 93.926 |
| E5 | 45 | 61 | male | bladder | urothelial carcinoma | T2aN0M0 | 3 | II | tumor | 78.415 |
| E6 | 46 | 61 | male | bladder | pericarcinomatous tissue | - | - | - | normal | 104.298 |
| E7 | 47 | 73 | male | bladder | urothelial carcinoma | T2aN0M0 | 3 | II | tumor | 158.201 |
| E8 | 48 | 73 | male | bladder | pericarcinomatous tissue | - | - | - | normal | 116.145 |
| E9 | 49 | 66 | male | bladder | urothelial carcinoma | T1N0M0 | 1 | I | tumor | 137.595 |
| E10 | 50 | 66 | male | bladder | pericarcinomatous tissue | - | - | - | normal | 49.024 |
| F1 | 51 | 67 | male | bladder | urothelial carcinoma | T1N0M0 | 1 | I | tumor | 22.828 |
| F2 | 52 | 67 | male | bladder | pericarcinomatous tissue | - | - | - | normal | 45.709 |
| F3 | 53 | 78 | male | bladder | urothelial carcinoma | T2aN0M0 | 3 | II | tumor | 119.288 |
| F4 | 54 | 78 | male | bladder | pericarcinomatous tissue | - | - | - | normal | 79.295 |
| F5 | 55 | 72 | male | bladder | urothelial carcinoma | T1N0M0 | 1 | I | tumor | 67.043 |
| F6 | 56 | 72 | male | bladder | pericarcinomatous tissue | - | - | - | normal | 87.239 |
| F7 | 57 | 66 | male | bladder | urothelial carcinoma | T2aN0M0 | 3 | II | tumor | 49.822 |
| F8 | 58 | 66 | male | bladder | pericarcinomatous tissue | - | - | - | normal | 57.295 |
| F9 | 59 | 59 | male | bladder | urothelial carcinoma | T2bN1M0 | 3 | II | tumor | 113.449 |
| F10 | 60 | 59 | male | bladder | pericarcinomatous tissue | - | - | - | normal | 74.959 |
| G1 | 61 | 52 | male | bladder | urothelial carcinoma | TisN0M0 | 1 | Ois | tumor | 38.036 |
| G2 | 62 | 52 | male | bladder | pericarcinomatous tissue | - | - | - | normal | 107.508 |
| G3 | 63 | 67 | male | bladder | urothelial carcinoma | T3N1M0 | 3 | IIIA | tumor | 60.278 |
| G4 | 64 | 67 | male | bladder | pericarcinomatous tissue | - | - | - | normal | 58.095 |
| G5 | 65 | 62 | male | bladder | urothelial carcinoma | T4aN0M0 | 3 | IIIA | tumor | 49.979 |
| G6 | 66 | 62 | male | bladder | pericarcinomatous tissue | - | - | - | normal | 86.767 |
| G7 | 67 | 65 | male | bladder | urothelial carcinoma | T1N0M0 | 3 | I | tumor | 64.688 |
| G8 | 68 | 65 | male | bladder | pericarcinomatous tissue | - | - | - | normal | 125.716 |
| G9 | 69 | 64 | male | bladder | urothelial carcinoma | TisN0M0 | 3 | Ois | tumor | 26.48 |

**Table S3**

| Protein Group | Protein ID | Accession | -10lgP | Coverage (%) | Coverage (%) 1-FLAG | Area 1-FLAG | #Peptides | #Unique | #Spec 1-FLAG | PTM | Avg. Mass | Description |
| --- | --- | --- | --- | --- | --- | --- | --- | --- | --- | --- | --- | --- |
| 1 | 5 | sp|Q9UNE7|CHIP_HUMAN | 83 | 83 | 5.48E+09 | 66 | 61 | 224 | Carbamidomethylation; Deamidation (NQ); Oxidation (M); Pyro-glu from E | 34856 | E3 ubiquitin-protein ligase CHIP OS=Homo sapiens OX=9606 GN=STUB1 PE=1 SV=2 | |
| 50 | 99 | sp|P54652|HSP72_HUMAN | 201.2 | 21 | 21 | 3.87E+06 | 14 | 1 | 32 | Carbamidomethylation; Deamidation (NQ) | 70021 | Heat shock-related 70 kDa protein 2 OS=Homo sapiens OX=9606 GN=HSPA2 PE=1 SV=1 |
| 233 | 155 | sp|P62979|RS27A_HUMAN | 112.6 | 35 | 35 | 1.14E+07 | 6 | 6 | 6 |  | 17965 | Ubiquitin-40S ribosomal protein S27a OS=Homo sapiens OX=9606 GN=RPS27A PE=1 SV=2 |
| 278 | 236 | sp|P05198|IF2A_HUMAN | 97.9 | 17 | 17 | 1.61E+06 | 5 | 5 | 5 |  | 36112 | Eukaryotic translation initiation factor 2 subunit 1 OS=Homo sapiens OX=9606 GN=EIF2S1 PE=1 SV=3 |
| 338 | 293 | sp|P00505|AATM_HUMAN | 78 | 7 | 7 | 4.97E+05 | 3 | 3 | 3 |  | 47518 | Aspartate aminotransferase mitochondrial OS=Homo sapiens OX=9606 GN=GOT2 PE=1 SV=3 |
| 430 | 288 | sp|P47929|LEG7_HUMAN | 103.6 | 29 | 29 | 5.86E+06 | 3 | 3 | 3 |  | 15075 | Galectin-7 OS=Homo sapiens OX=9606 GN=LGALS7B PE=1 SV=2 |
| 431 | 297 | sp|P29692|EF1D_HUMAN | 90.64 | 17 | 17 | 1.06E+06 | 3 | 3 | 3 |  | 31122 | Elongation factor 1-delta OS=Homo sapiens OX=9606 GN=EEF1D PE=1 SV=5 |
| 433 | 296 | sp|P08133|ANXA6_HUMAN | 80.52 | 6 | 6 | 7.72E+05 | 3 | 3 | 3 |  | 75873 | Annexin A6 OS=Homo sapiens OX=9606 GN=ANXA6 PE=1 SV=3 |

**Table S4**

| Metabolites | Control-1 | Control-2 | Control-3 | Control-4 | Control-5 | Control-6 | Flag-STUB1-1 | Flag-STUB1-2 | Flag-STUB1-3 | Flag-STUB1-4 | Flag-STUB1-5 | Flag-STUB1-6 |
| --- | --- | --- | --- | --- | --- | --- | --- | --- | --- | --- | --- | --- |
| L-Aspartic acid | 5033.539 | 4056.746 | 4536.483 | 4509.357 | 4043.505 | 4470.852 | 942.2344 | 875.0219 | 1070.12 | 977.549 | 1046.572 | 846.8503 |
| L-Alanine | 13179.32 | 11120.22 | 13994.55 | 13190.26 | 12995.07 | 14431.2 | 1963.583 | 1261.239 | 2824.749 | 1865.536 | 2913.251 | 2075.187 |
| L-Serine | 1135.287 | 871.3462 | 745.8989 | 987.6832 | 813.0586 | 912.1188 | 2020.218 | 2091.288 | 2286.322 | 2102.568 | 2201.604 | 2273.085 |
| L-Tryptophan | 605.1366 | 666.363 | 671.8613 | 631.7284 | 652.9412 | 644.383 | 1425.993 | 1563.566 | 1312.287 | 1473.582 | 1258.12 | 1514.914 |
| S-Adenosylhomocysteine | 87.9095 | 73.21945 | 78.17776 | 74.79192 | 78.15195 | 77.20643 | 27.93531 | 16.93971 | 37.51761 | 22.1617 | 33.60485 | 24.92006 |
| 4-Hydroxyproline | 170.2097 | 135.351 | 143.1284 | 180.3968 | 151.7864 | 192.3729 | 28.62376 | 13.02727 | 39.14617 | 26.8663 | 28.24485 | 35.09574 |
| L-Valine | 2163.537 | 2085.411 | 2176.615 | 2488.694 | 2313.374 | 2547.083 | 4387.831 | 5669.358 | 5184.352 | 5052.66 | 4718.825 | 5489.542 |
| L-Tyrosine | 1850.471 | 1587.225 | 2137.787 | 1923.287 | 1788.24 | 2229.082 | 4246.015 | 5059.135 | 4350.017 | 4299.905 | 3907.767 | 4945.874 |
| L-Glutamine | 6135.569 | 5033.158 | 5225.958 | 5791.428 | 5547.233 | 5851.507 | 2463.774 | 1990.941 | 3265.01 | 2487.676 | 2765.976 | 2775.441 |
| Ornithine | 818.8994 | 643.9571 | 492.6294 | 695.7922 | 663.9194 | 725.2666 | 185.6048 | 90.03888 | 108.3827 | 121.1887 | 130.005 | 211.6608 |
| L-Glycine | 2482.598 | 1807.363 | 1706.119 | 2002.181 | 1915.429 | 2116.384 | 4987.385 | 4843.421 | 7090.896 | 5273.037 | 6308.877 | 6111.579 |
| L-alpha-Aminobutyricacid | 15.20149 | 10.87558 | 9.591729 | 15.46305 | 13.77802 | 17.58966 | 1.721894 | 2.022799 | 1.191151 | 0.948818 | 1.543291 | 2.326534 |
| NAlpha-Acetyl-L-glutamine | 4.154894 | 2.789398 | 2.772866 | 2.843947 | 3.014871 | 2.598132 | 0.342344 | 0.387137 | 1.090419 | 0.426283 | 0.704772 | 0.604901 |
| N-Acetyl-L-asparticacid | 713.8412 | 778.5942 | 744.2342 | 857.0091 | 593.3562 | 833.5804 | 233.3347 | 297.2141 | 458.1406 | 259.456 | 280.2457 | 239.9369 |
| SuccinicAcid | 1779.935 | 1344.464 | 1209.757 | 1434.624 | 1489.011 | 1495.602 | 604.3539 | 503.399 | 1041.323 | 604.759 | 868.7859 | 682.6916 |
| ethanolamine | 61.15314 | 40.48911 | 30.2946 | 29.46088 | 53.17936 | 42.37625 | 9.291245 | 6.71209 | 19.74937 | 4.81107 | 20.45715 | 1.9612 |
| L-Homoserine | 2.083345 | 1.821905 | 2.872559 | 5.349399 | 4.673389 | 1.715378 | 0.003659 | 0.003659 | 0.003659 | 0.003659 | 0.003659 | 0.003659 |
| Homocitrulline | 2.607476 | 1.741078 | 1.403045 | 1.37363 | 1.491885 | 2.624825 | 0.448483 | 0.385994 | 0.417281 | 0.761098 | 1.078765 | 1.382921 |
| beta-Alanine | 133.6928 | 112.9073 | 102.7267 | 143.0807 | 109.8542 | 178.1835 | 0.003659 | 0.003659 | 125.9764 | 75.81527 | 0.003659 | 77.67505 |
| gamma-Glutamylalanine | 5.961706 | 29.42851 | 5.762101 | 11.56027 | 8.582989 | 9.922056 | 0.003659 | 0.003659 | 0.003659 | 0.003659 | 0.003659 | 0.003659 |
| Homocysteine | 0.923998 | 0.032043 | 3.113177 | 0.164385 | 3.166782 | 1.901871 | 5.631717 | 9.861025 | 4.144727 | 12.48974 | 3.68298 | 4.228575 |
| 2-Methylhippuricacid | 0.397949 | 0.003659 | 0.374673 | 0.373737 | 0.003659 | 0.427856 | 0.413008 | 0.668197 | 0.516925 | 0.426267 | 0.651749 | 0.571406 |
| gamma-Aminobutyricacid | 110.4547 | 168.3643 | 65.13908 | 67.44529 | 83.59107 | 83.8325 | 22.19442 | 70.07604 | 69.85275 | 30.96958 | 49.48424 | 42.30787 |
| NAlpha-Acetyl-L-Arginine | 0.242868 | 0.132496 | 0.114605 | 0.142872 | 0.115397 | 0.064981 | 0.029408 | 0.088546 | 0.08848 | 0.075346 | 0.046466 | 0.075809 |
| D-2-Aminobutyricacid | 110.5696 | 200.1761 | 61.35027 | 79.76221 | 83.51711 | 86.47087 | 29.51507 | 73.96474 | 75.17651 | 29.71362 | 54.29094 | 47.54069 |

**Table** S5

| ID | Position | Code | E3 enzymes | Peptide | Score | Cutoff |
| --- | --- | --- | --- | --- | --- | --- |
| unnamed | 52 | K | General | DPILGVTEAFKRDTNSKKMNL | 0.419 | 0.2655 |
| unnamed | 59 | K | General | EAFKRDTNSKKMNLGVGAYRD | 0.5853 | 0.2655 |
| unnamed | 73 | K | General | GVGAYRDDNGKPYVLPSVRKA | 0.7656 | 0.2655 |
| unnamed | 82 | K | General | GKPYVLPSVRKAEAQIAAKNL | 0.361 | 0.2655 |
| unnamed | 90 | K | General | VRKAEAQIAAKNLDKEYLPIG | 0.5234 | 0.2655 |
| unnamed | 94 | K | General | EAQIAAKNLDKEYLPIGGLAE | 0.4383 | 0.2655 |
| unnamed | 122 | K | General | LALGENSEVLKSGRFVTVQTI | 0.6573 | 0.2655 |
| unnamed | 227 | K | General | TGVDPRPEQWKEIATVVKKRN | 0.3829 | 0.2655 |
| unnamed | 256 | K | General | YQGFASGDGDKDAWAVRHFIE | 0.4544 | 0.2655 |
| unnamed | 296 | K | General | ERVGAFTMVCKDADEAKRVES | 0.6006 | 0.2655 |
| unnamed | 302 | K | General | TMVCKDADEAKRVESQLKILI | 0.4486 | 0.2655 |
| unnamed | 309 | K | General | DEAKRVESQLKILIRPMYSNP | 0.4615 | 0.2655 |
| unnamed | 338 | K | General | AAILNTPDLRKQWLQEVKVMA | 0.4952 | 0.2655 |
| unnamed | 345 | K | General | DLRKQWLQEVKVMADRIIGMR | 0.5787 | 0.2655 |
| unnamed | 363 | K | General | GMRTQLVSNLKKEGSTHNWQH | 0.3446 | 0.2655 |
| unnamed | 364 | K | General | MRTQLVSNLKKEGSTHNWQHI | 0.2909 | 0.2655 |
| unnamed | 396 | K | General | LKPEQVERLIKEFSIYMTKDG | 0.7701 | 0.2655 |
| unnamed | 404 | K | General | LIKEFSIYMTKDGRISVAGVT | 0.6656 | 0.2655 |

**TableS6**

| Location | Age | Pathology | TNM | Grade | Stage | GOT2-SCORE |
| --- | --- | --- | --- | --- | --- | --- |
| A1 | 64 | urothelial carcinoma | T2bN0M0 | 3 | II | 169.378 |
| A2 | 64 | pericarcinomatous tissue | - | - | - | 108.486 |
| A3 | 87 | urothelial carcinoma | T2aN0M0 | 1 | II | 169.274 |
| A4 | 87 | pericarcinomatous tissue | - | - | - | 102.794 |
| A5 | 50 | urothelial carcinoma | T2aN0M0 | 1 | II | 143.19 |
| A6 | 50 | pericarcinomatous tissue | - | - | - | 166.118 |
| A7 | 69 | urothelial carcinoma | T4N0M0 | 3 | IIIA | 207.534 |
| A8 | 69 | pericarcinomatous tissue | - | - | - | 124.606 |
| A9 | 61 | urothelial carcinoma | T1N0M0 | 3 | I | 165.231 |
| A10 | 61 | pericarcinomatous tissue | - | - | - | 154.527 |
| B1 | 54 | urothelial carcinoma | T2bN0M0 | 3 | II | 138.751 |
| B2 | 54 | pericarcinomatous tissue | - | - | - | 162.589 |
| B3 | 64 | urothelial carcinoma | T1N0M0 | 1 | I | 185.459 |
| B4 | 64 | pericarcinomatous tissue | - | - | - | 165.323 |
| B5 | 68 | urothelial carcinoma | T2bN0M0 | 3 | II | 170.004 |
| B6 | 68 | pericarcinomatous tissue | - | - | - | 160.51 |
| B7 | 65 | urothelial carcinoma | T3aN0M0 | 3 | IIIA | 179.327 |
| B8 | 65 | pericarcinomatous tissue | - | - | - | 156.99 |
| B9 | 64 | urothelial carcinoma | T3aN0M0 | 2 | IIIA | 176.8 |
| B10 | 64 | pericarcinomatous tissue | - | - | - | 182.266 |
| C1 | 67 | urothelial carcinoma | T1N0M0 | 3 | I | 194.05 |
| C2 | 67 | pericarcinomatous tissue | - | - | - | 167.78 |
| C3 | 52 | urothelial carcinoma | T2aN0M0 | 3 | II | 169.013 |
| C4 | 52 | pericarcinomatous tissue | - | - | - | 149.445 |
| C5 | 76 | urothelial carcinoma | T2aN0M0 | 3 | II | 183.379 |
| C6 | 76 | pericarcinomatous tissue | - | - | - | 135.359 |
| C7 | 70 | urothelial carcinoma | T4aN2M0 | 3 | IIIB | 168.831 |
| C8 | 70 | pericarcinomatous tissue | - | - | - | 180.597 |
| C9 | 70 | urothelial carcinoma | T2aN0M0 | 3 | II | 138.178 |
| C10 | 70 | pericarcinomatous tissue | - | - | - | 194.01 |
| D1 | 77 | urothelial carcinoma | T1N0M0 | 3 | I | 197.059 |
| D2 | 77 | pericarcinomatous tissue | - | - | - | 162.094 |
| D3 | 41 | urothelial carcinoma | T3aN0M0 | 3 | III | 176.532 |
| D4 | 41 | pericarcinomatous tissue | - | - | - | 120.862 |
| D5 | 92 | urothelial carcinoma | T4aN1M0 | 3 | IIIA | 168.255 |
| D6 | 92 | pericarcinomatous tissue | - | - | - | 140.439 |
| D7 | 82 | urothelial carcinoma | T2aN0M0 | 3 | II | 166.993 |
| D8 | 82 | pericarcinomatous tissue | - | - | - | 162.021 |
| D9 | 72 | urothelial carcinoma | T2bN1M0 | 3 | IIIA | 187.864 |
| D10 | 72 | pericarcinomatous tissue | - | - | - | 165.924 |
| E1 | 60 | urothelial carcinoma | T2bN1M0 | 3 | IIIA | 147.647 |
| E2 | 60 | pericarcinomatous tissue | - | - | - | 126.104 |
| E3 | 78 | urothelial carcinoma | T2bN0M0 | 3 | II | 196.874 |
| E4 | 78 | pericarcinomatous tissue | - | - | - | 163.65 |
| E5 | 61 | urothelial carcinoma | T2aN0M0 | 3 | II | 170.253 |
| E6 | 61 | pericarcinomatous tissue | - | - | - | 160.238 |
| E7 | 73 | urothelial carcinoma | T2aN0M0 | 3 | II | 155.917 |
| E8 | 73 | pericarcinomatous tissue | - | - | - | 157.225 |
| E9 | 66 | urothelial carcinoma | T1N0M0 | 1 | I | 166.42 |
| E10 | 66 | pericarcinomatous tissue | - | - | - | 182.707 |
| F1 | 67 | urothelial carcinoma | T1N0M0 | 1 | I | 165.835 |
| F2 | 67 | pericarcinomatous tissue | - | - | - | 167.716 |
| F3 | 78 | urothelial carcinoma | T2aN0M0 | 3 | II | 157.891 |
| F4 | 78 | pericarcinomatous tissue | - | - | - | 180.794 |
| F5 | 72 | urothelial carcinoma | T1N0M0 | 1 | I | 154.014 |
| F6 | 72 | pericarcinomatous tissue | - | - | - | 135.067 |
| F7 | 66 | urothelial carcinoma | T2aN0M0 | 3 | II | 175.519 |
| F8 | 66 | pericarcinomatous tissue | - | - | - | 169.058 |
| F9 | 59 | urothelial carcinoma | T2bN1M0 | 3 | II | 203.859 |
| F10 | 59 | pericarcinomatous tissue | - | - | - | 188.262 |
| G1 | 52 | urothelial carcinoma | TisN0M0 | 1 | Ois | 136.7 |
| G2 | 52 | pericarcinomatous tissue | - | - | - | 155.69 |
| G3 | 67 | urothelial carcinoma | T3N1M0 | 3 | IIIA | 175.699 |
| G4 | 67 | pericarcinomatous tissue | - | - | - | 160.7 |
| G5 | 62 | urothelial carcinoma | T4aN0M0 | 3 | IIIA | 172.469 |
| G6 | 62 | pericarcinomatous tissue | - | - | - | 152.174 |
| G7 | 65 | urothelial carcinoma | T1N0M0 | 3 | I | 212.716 |
| G8 | 65 | pericarcinomatous tissue | - | - | - | 160.07 |
| G9 | 64 | urothelial carcinoma | TisN0M0 | 3 | Ois | 193.55 |

**Supplementary Materials and Methods**

**Antibodies and reagents**

Anti-GOT2 antibody (Cat No. 14800-1-AP), anti-β-actin antibody (Cat No. HRP-66009), anti-HA antibody (Cat No. HRP-81290), anti-Flag antibody (Cat No. 66008-4-Ig) and anti-Myc antibody (Cat No. 16286-1-AP) were purchased from Proteintech (Wuhan, China). Anti-STUB1 antibody (M01236-2) was purchased from Boster (Wuhan, China) and anti-Ki67 antibody (ab15580) were purchased from Abcam (Cambridge, United Kingdom). All were used according to the manufacturers’ recommendations.

**Plasmids and cloning**

Flag-STUB1， MYC-STUB1， Flag-GOT2 and Flag-GOT1 expression plasmid were generated by subcloning the into pcDNA3 vector and were obtained from MiaoLingBio, China. STUB1 different domains were subcloned into the pcDNA3-Flag vector. Primers used for cloning are available upon request.

**shRNA lentiviral vector packaging and transduction**

shRNA lentiviral vector packaging and transduction were described as previously[^1^](#_ENREF_1). lentiviral vectors shRNA-STUB1, shRNA-GOT2 and shRNA-GFP pLKO1 (control vector) were purchased from QEgene (Shanghai, China). Lentiviral vector encoding shRNA was packaged in 293T cells by calcium phosphate transfection. The supernatants that contained lentiviral particles was collected 48h after transfection. The indicated BCa cells were then transduced with the supernatant in the presence of polybrene (8 μg/mL) for 24 h before replacement with fresh growth media. Cells were analyzed at 48 or 72 h post transduction.

**Quantitative real-time PCR (qRT-PCR)**

qRT-PCR was described as previously[^1^](#_ENREF_1). Primers for qPCR analysis of human gene transcripts were:

STUB1:

Forward Primer: 5’- GAGGCCAAGCACGACAAG-3’

Reverse Primer: 5’- CTCCCGCATCAGCTCAAA-3’

β-actin：

Forward Primer: 5’- TCTCCCAAGTCCACACAGG -3’

Reverse Primer: 5’- GGCACGAAGGCTCATCA -3’

GOT2:

Forward Primer: 5’- CCAAGGCTTTGCCAGTGGTGAT-3’

Reverse Primer: 5’- AGTGAAGGCTCCTACACGCTCA-3’

Western blotting

Proteins were prepared as previously described[^2^](#_ENREF_2). Briefly, protein extracts were separated by gradient SDS-PAGE gel and then electroblotted onto a PVDF membrane (Cytiva, catalog number: 10600021). The membranes were incubated with the indicated primary antibodies at 1: 1000 at 4°C overnight, respectively, followed by incubation with corresponding secondary antibodies at 1: 10000 at room temperature for 1 h.

Cell proliferation and clony formation assays

Cell proliferation, clony formation and soft agar colony-formation assays were described as previously[^3^](#_ENREF_3).

Migration

Migration results were performed as previously described[^3^](#_ENREF_3), using transwell assays according to the manufacturer’s directions (Corning Inc., Corning, NY). The indicated BCa cells (1 × 10^5^) were plated in the top well in no FBS RPMI MEM or DMEM medium. In the bottom well, control media, CM or different concentrations of recombinant GDF15 (Cat.8944-GD-025, R&D Systems Minneapolis, MN) and 10% FBS were added as a chemoattractant. Cells underneath the inserts were analyzed using light microscopy and photographed. The numbers of cells were counted in five random fields for each insert. Each data point represents the average number from three wells.

CCK-8 assay

The indicated cells were respectively seeded in 96-well culture plates (1 × 10^4^/well) and placed at 37 °C, Incubate overnight in a 5% CO2 incubator. Wash the cells inoculated the previous day with PBS twice, then add 100 μl DMEM medium for use. Discard the old solution at 48 h after changing the medium, add 95 μl of medium and 5ul of CCK-8 to each well of the 96-well plate to be tested, and incubate at 37 °C for 2 h. A microplate reader (BioTek Instruments, Inc., Winooski, VT, USA) was used to measure the OD value of each experimental well at 450 nm, and to detect changes in cell proliferation ability of each group.

**Edu assay**

The Edu proliferation assay using the Cell-Light™ Edu Apollo In Vitro Kit from RiboBio involves infecting T24 and UC3 cells with indicated lentivirus, digesting and resuspending the cells in 2 mL of complete medium, and seeding them into 96-well plates at a density of 6000 cells per well. The cells are treated with 50 μM Edu working solution for 2 hours, then fixed with 4% paraformaldehyde for 20 minutes, permeabilized with 0.5% Triton X-100, and stained with Apollo reaction cocktail and DAPI. Fluorescence microscopy is used to capture images of Edu-labeled proliferating cells, and ImageJ software analyzes the percentage of Edu-positive cells for statistical analysis.

Protein immunoprecipitation (IP) and Liquid Chromatography-MS Analysis

Co-IP or IP was described as previously[^1^](#_ENREF_1). Cell lysates were captured on Flag-M2 beads or protein A/G agarose beads (Santa Cruz, USA). The complexes were then separated, and the gels were stained with silver or were detected by Western blotting. For Liquid Chromatography-MS Analysis, immunoprecipitation of STUB1 antibodies was performed as described above. The precipitated proteins were eluted 3 times with lysis buffer. The eluted samples were subjected to in-solution trypsin digestion, followed by liquid chromatography-MS analysis.

Ubiquitination assay

Ubiquitination assay was described as previously[^1^](#_ENREF_1). In brief, the cells were transfected with indicated plasmids and lysed with the immunoprecipitation buffer. For immunoprecipitation, 2 mg of protein was incubated with indicated antibodies at 4°C overnight before Flag-M2 beads or protein A/G beads were added for 2 h. Beads were washed once with TBS, 1% Triton X-100, 1% SDS, twice with 0.5 M LiCl, TBS buffer and again in PBS 1% Triton X-100 containing buffer. Proteins were loaded onto 8% SDS-PAGE gels and immunoblotted with the indicated antibodies.

Immunofluorescence

**The indicated** cells were fixed in 4% paraformaldehyde for 15 min. After washing with PBS, the cover slides were blocked in PBS containing 0.1% Triton-X100, 2% BSA, and 1% normal goat serum for 1 h at room temperature and then incubated the slides with 1:500/1:1000 dilution of the indicated antibody for 2 h at room temperature. The Alexa 488-conjugated anti-rabbit/anti-mouse antibody or Rhodamine-conjugated anti-rabbit/anti- mouse antibody was added and incubated for 1 hr at room temperature. The slides were then washed three times with PBS and counterstained with 4,6-diamidino-2-phenylindole (DAPI) to visualize nuclei. The slides were captured by using an inverted microscope under a x 60 oil-immersion objective and scanned with a laser confocal system.

Aspartate Assay

Intracellular aspartate was quantified using a plate-based Aspartate Colorimetric Assay Kit (abcam, ab102512). The lysates (50 μL) from the indicated cells were incubated for 30 minutes with the enzyme reaction mixture (50 μL). Absorbance was measured at an optical density of 570 nm and aspartate concentration was extrapolated from a standard curve following background subtraction.

ATP Production measurement

Cellular ATP levels were measured using a firefly luciferase based ATP assay kit (Beyotime, Beijing, China) based on fluorescence technique.

ROS detection by fluorescence microscopy

After treatment, For cytosolic ROS detection, treated primary astrocytes were loaded with 10 μM DCFH-DA (Beyotime, S0033S, China) at 37 ℃ for 30 min. Cells were then washed with PBS three times before scanned by a microplate reader.

Mitochondrial Membrane Potential Measurement

For mitochondrial membrane potential measurement, the indicated live bladder cancer cells were stained with 10 μM TMRE (Beyotime, C2001S, China) for 30 minutes, followed by imaging using laser confocal microscopy.

Supplemental References

1. Xu S, Fan L, Jeon HY, Zhang F, Cui X, Mickle MB*, et al.* p300-Mediated Acetylation of Histone Demethylase JMJD1A Prevents Its Degradation by Ubiquitin Ligase STUB1 and Enhances Its Activity in Prostate Cancer. *Cancer research* 2020, **80**(15)**:** 3074-3087.

2. Xu SH, Huang JZ, Xu ML, Yu G, Yin XF, Chen D*, et al.* ACK1 promotes gastric cancer epithelial-mesenchymal transition and metastasis through AKT-POU2F1-ECD signalling. *The Journal of pathology* 2015, **236**(2)**:** 175-185.

3. Tang DE, Dai Y, He JX, Lin LW, Leng QX, Geng XY*, et al.* Targeting the KDM4B-AR-c-Myc axis promotes sensitivity to androgen receptor-targeted therapy in advanced prostate cancer. *The Journal of pathology* 2020, **252**(2)**:** 101-113.
