# Supplementary material for: Glucose-induced STUB1-GOT2 axis promotes aspartate synthesis and mitochondrial dysfunction in bladder cancer
Source: Cell Death Dis. 2025 Jul 12;16(1):516. doi: 10.1038/s41419-025-07840-5 (PMC12255758; doi:10.1038/s41419-025-07840-5)

FIGURE1

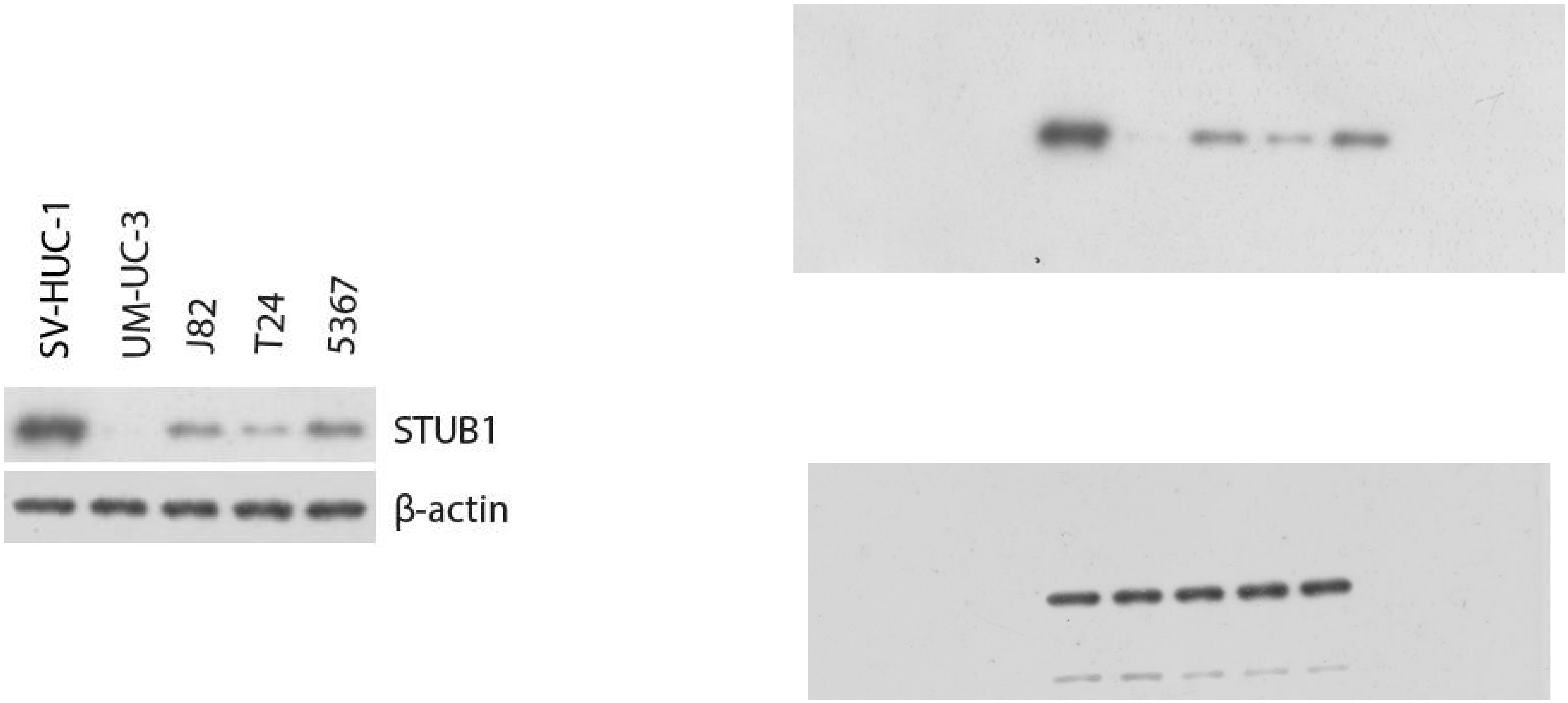

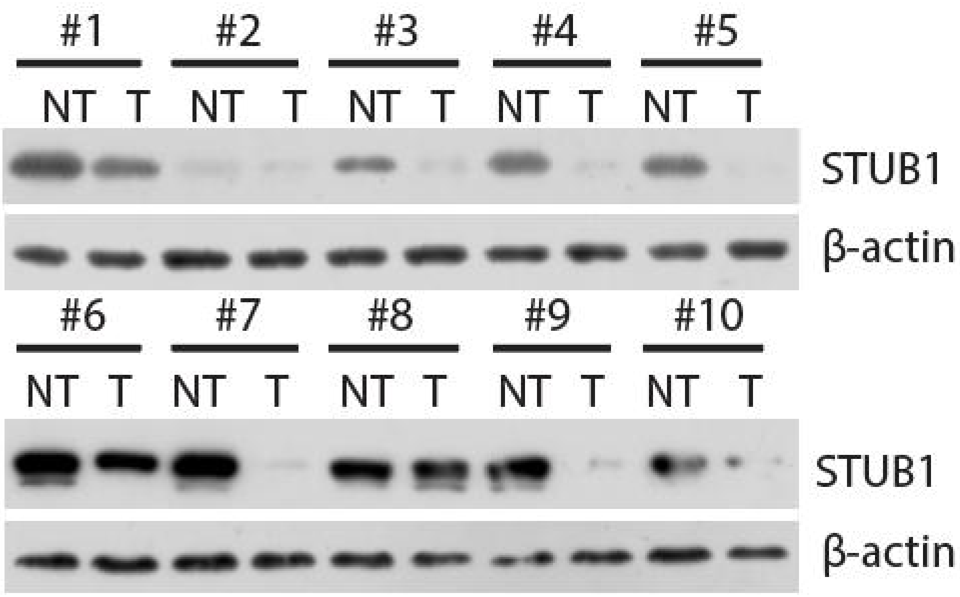
#1
#2
#3
#4
#5
NT T NT T NT T NT T NT T
STUB1
β-actin
#6
#7
#8
#9
#10
NT T NT T NT T NT T NT T
STUB1
β-actin

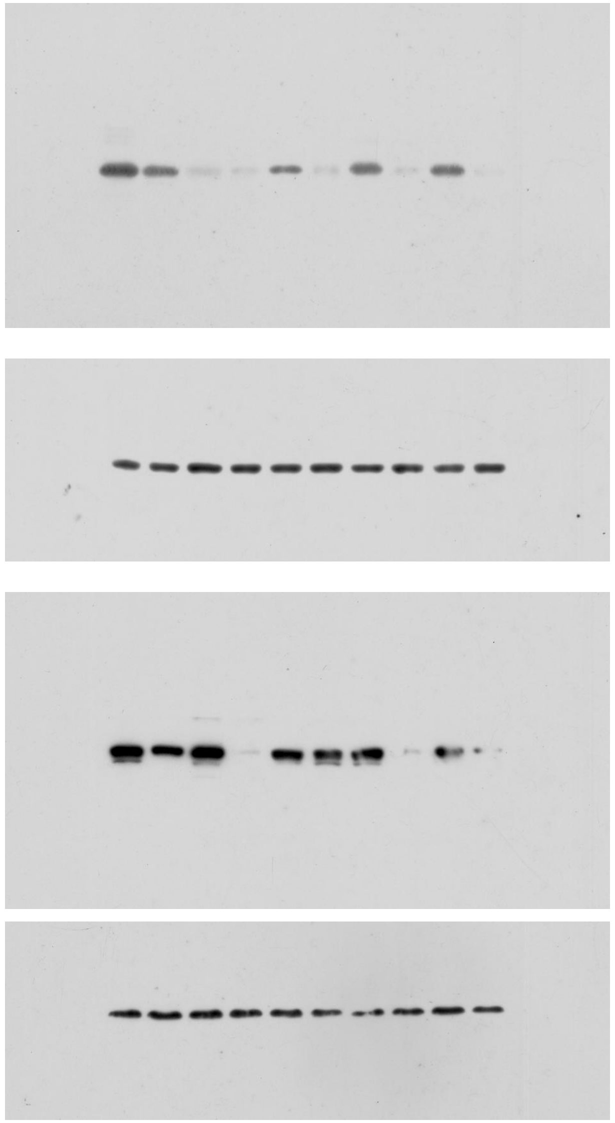

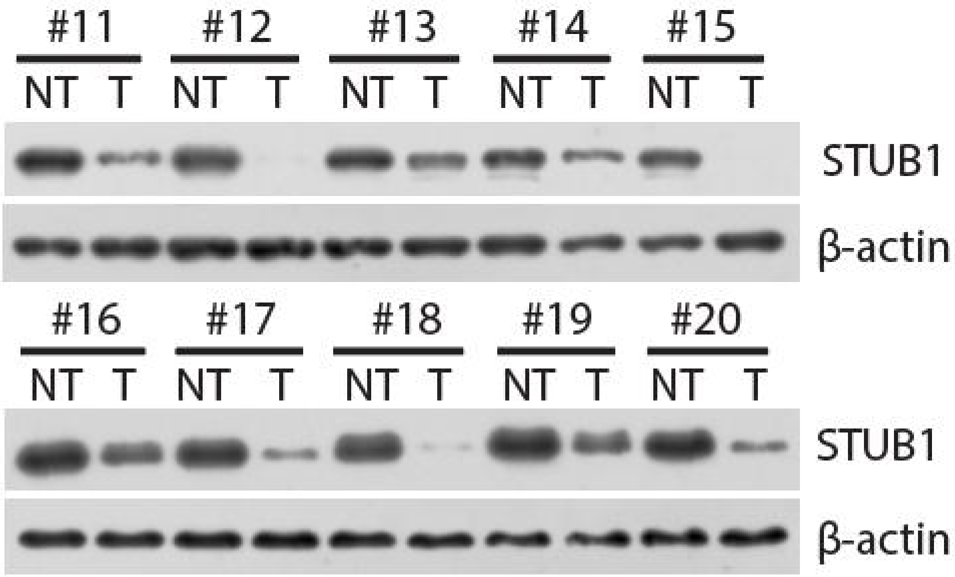
#11
#12
#13
#14
#15
NT T NT T NT T NT T NT T
STUB1
β-actin
#16
#17
#18
#19
#20
NT T NT T NT T NT T NT T
STUB1
β-actin

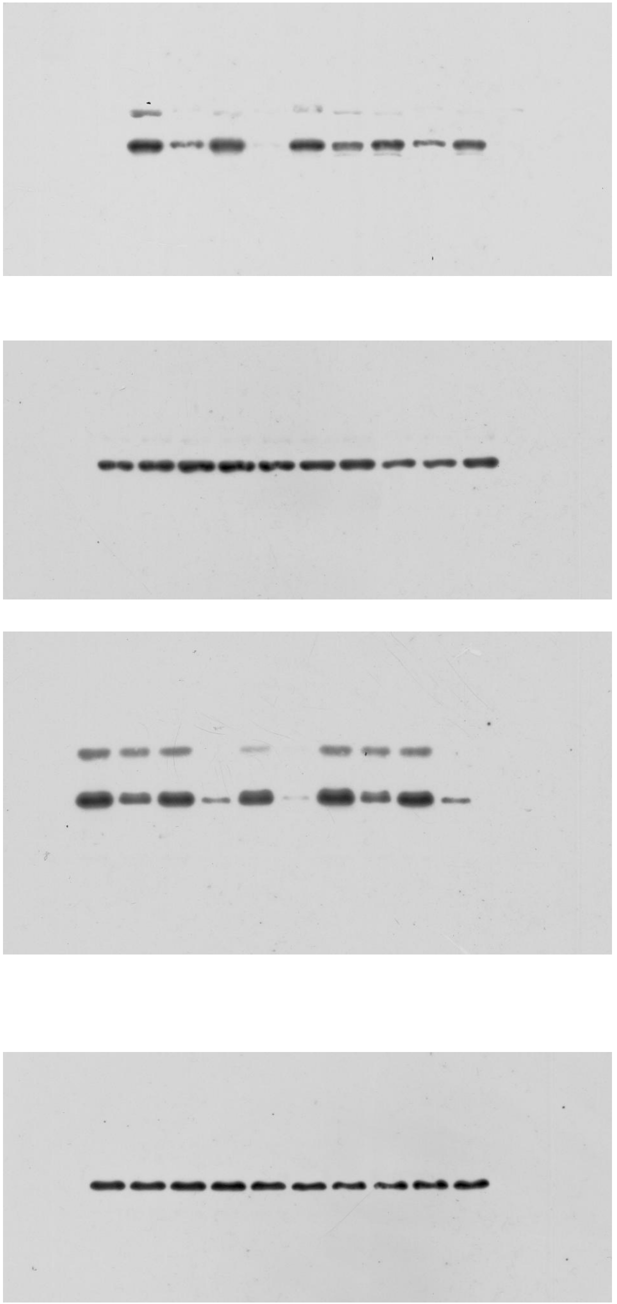

Figure4

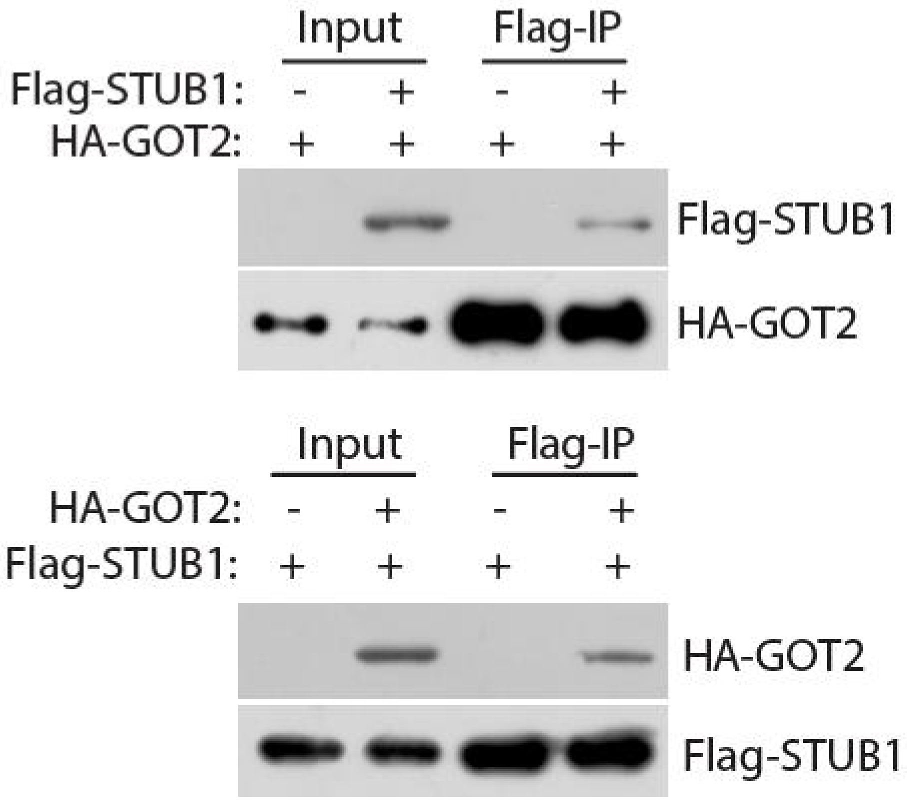
Input
Flag-IP
Flag-STUB1: - + - +
HA-GOT2: + + + +
Flag-STUB1
HA-GOT2
Input
Flag-IP
HA-GOT2: - + - +
Flag-STUB1: + + + +
HA-GOT2
Flag-STUB1

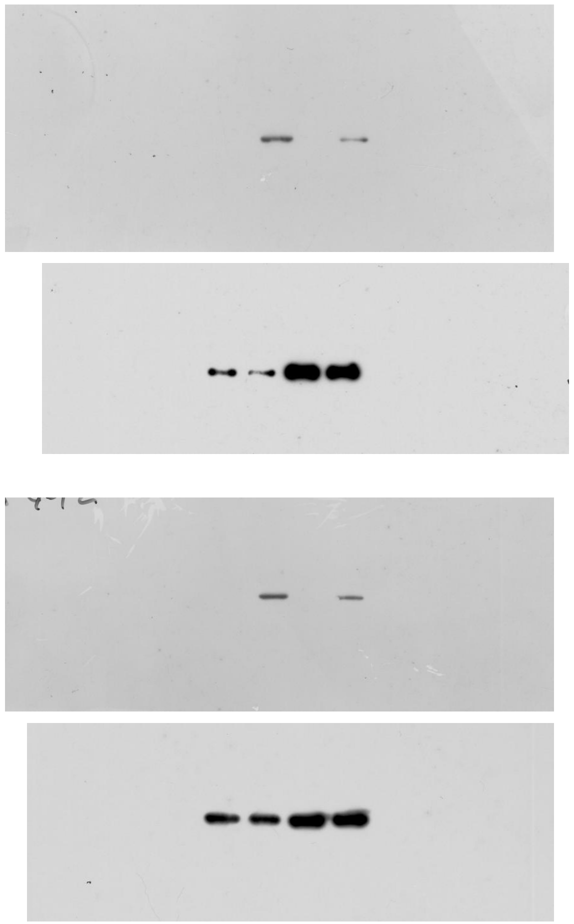

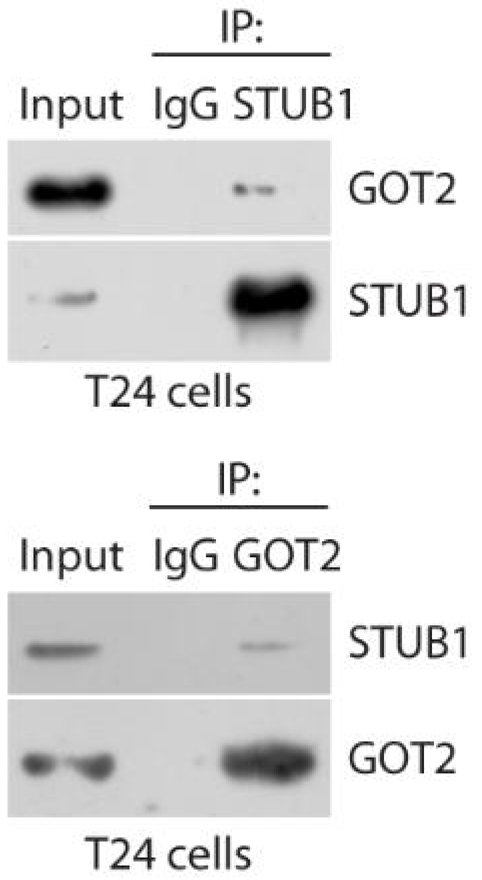
IP:
Input
IgG
STUB1
GOT2
STUB1
T24 cells
IP:
Input
IgG
GOT2
STUB1
GOT2
T24 cells

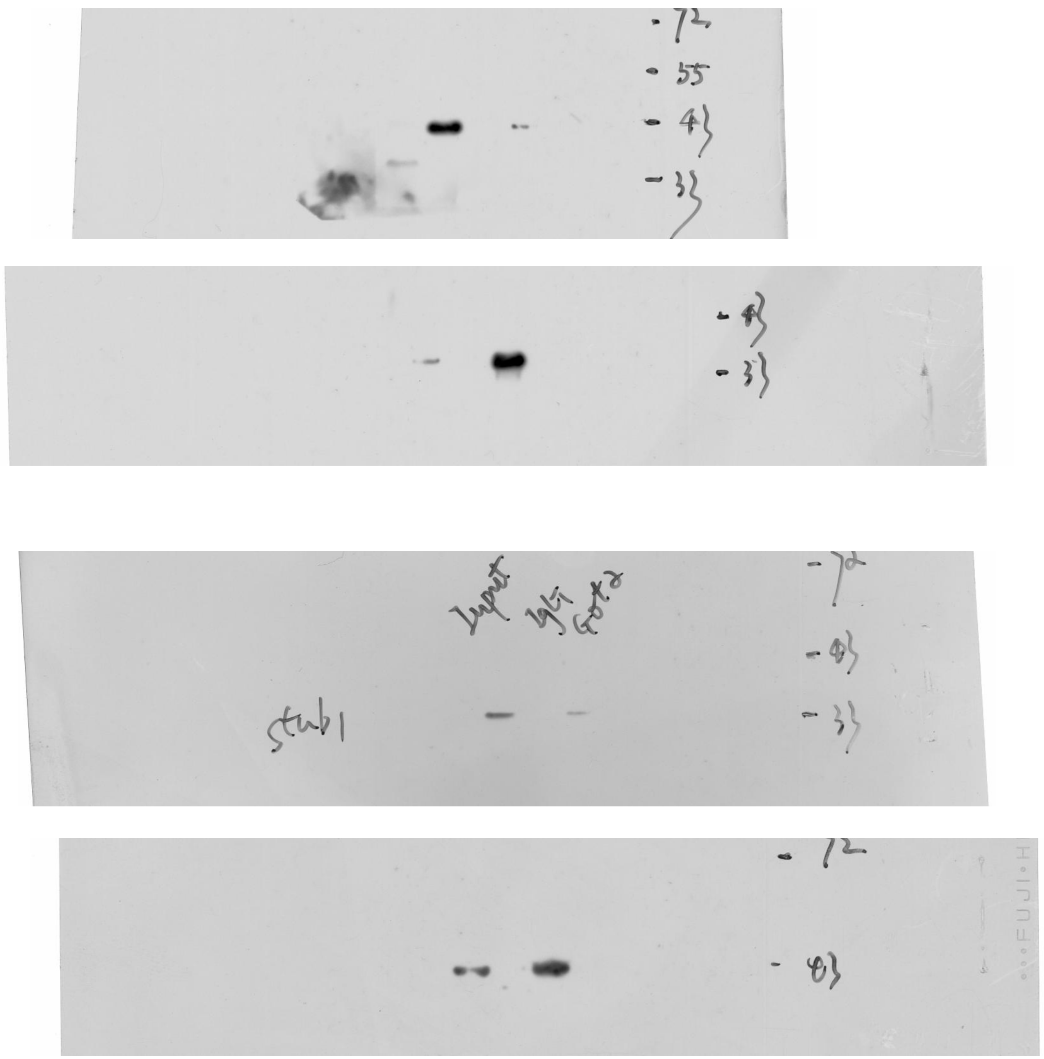

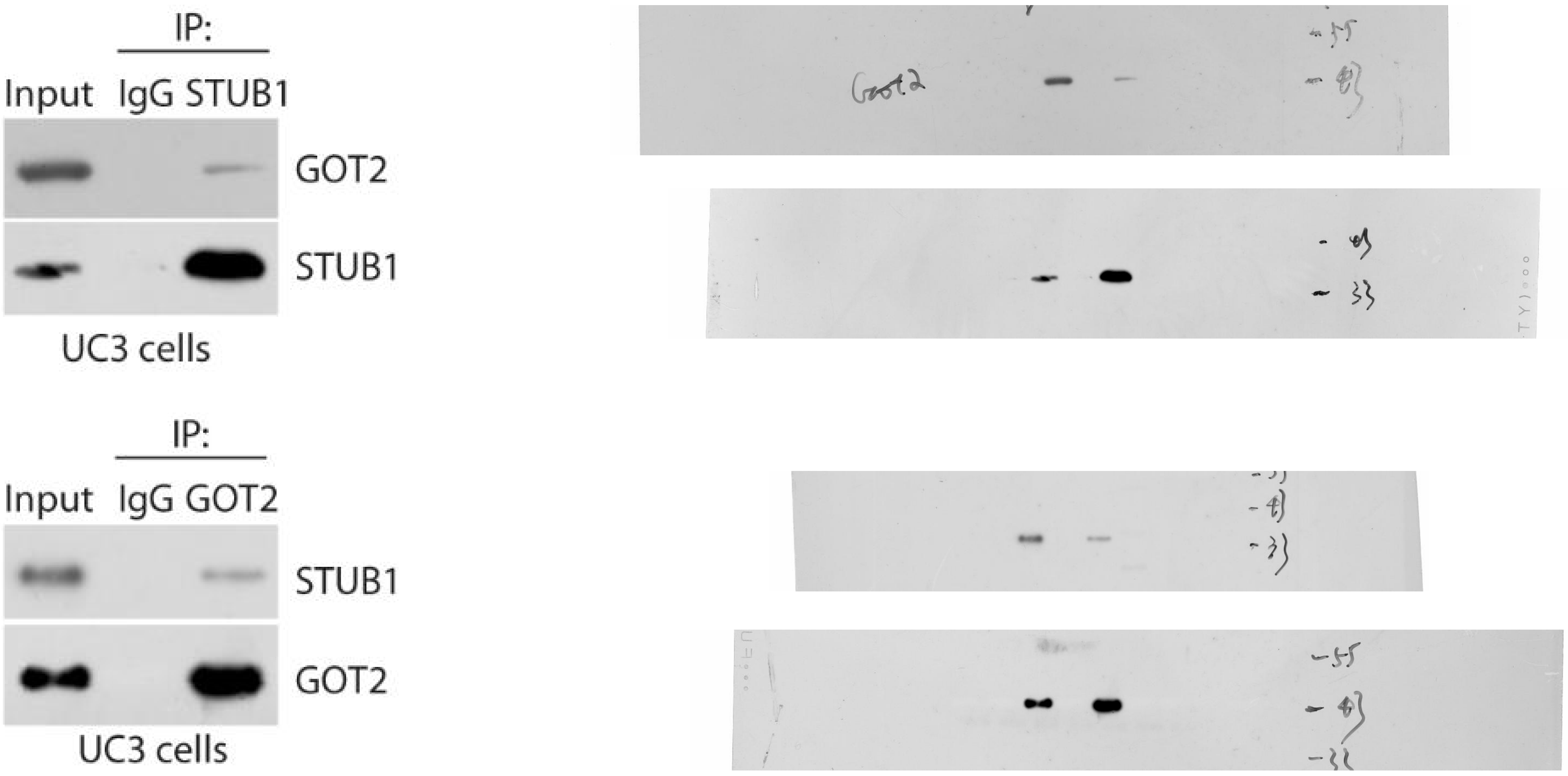
IP:
Input
IgG
STUB1
GOT2
STUB1
UC3 cells
IP:
Input
IgG
GOT2
STUB1
GOT2
UC3 cells

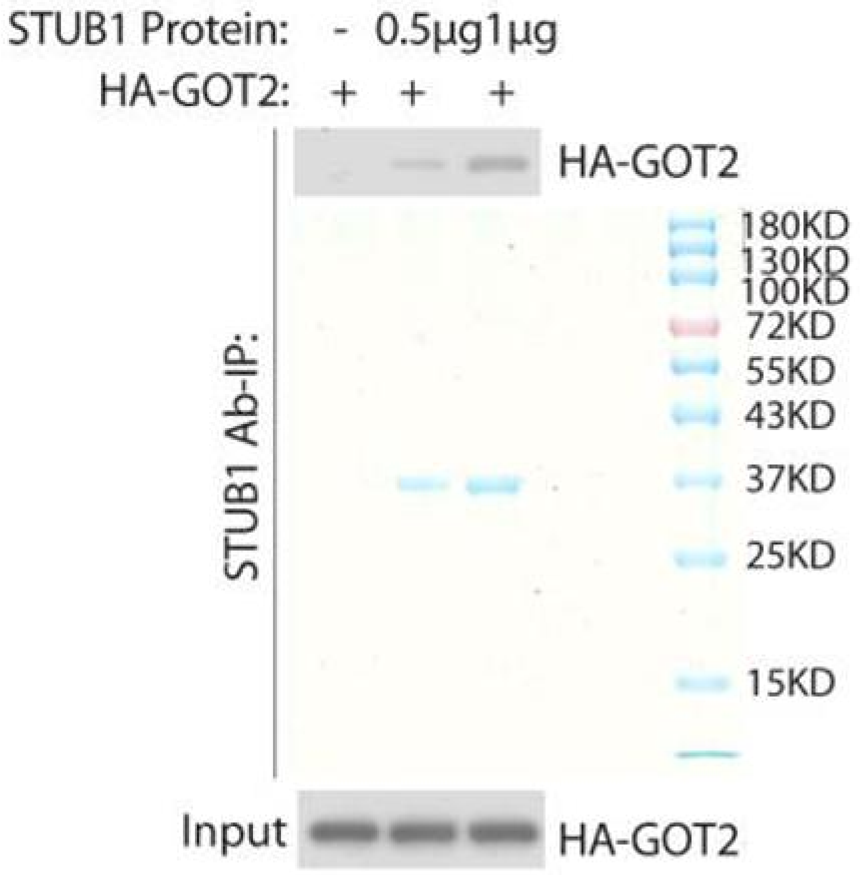
STUB1 Protein: - 0.5μg 1μg
HA-GOT2: + + +
HA-GOT2
STUB1 Ab-IP:
180KD
130KD
100KD
72KD
55KD
43KD
37KD
25KD
15KD
Input
HA-GOT2

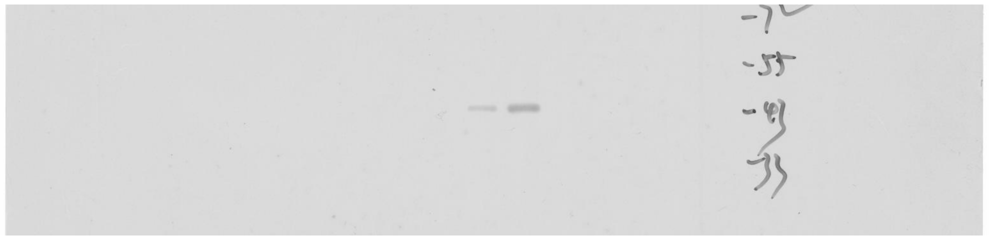

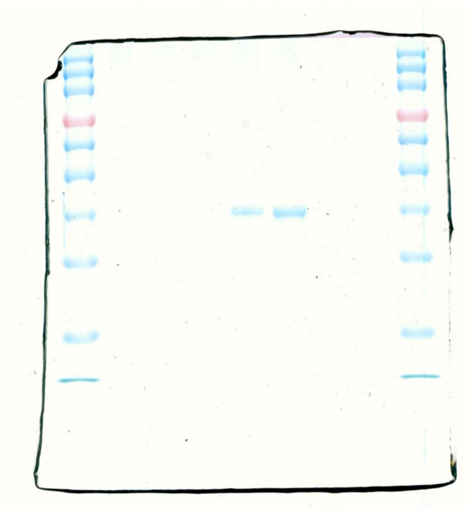

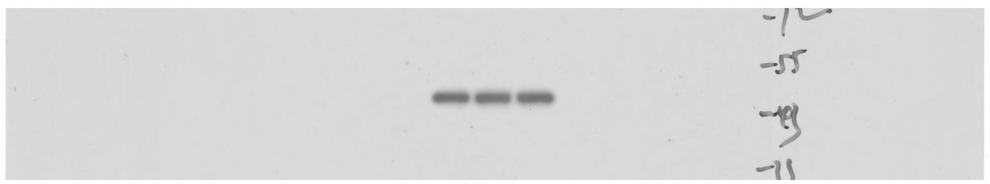

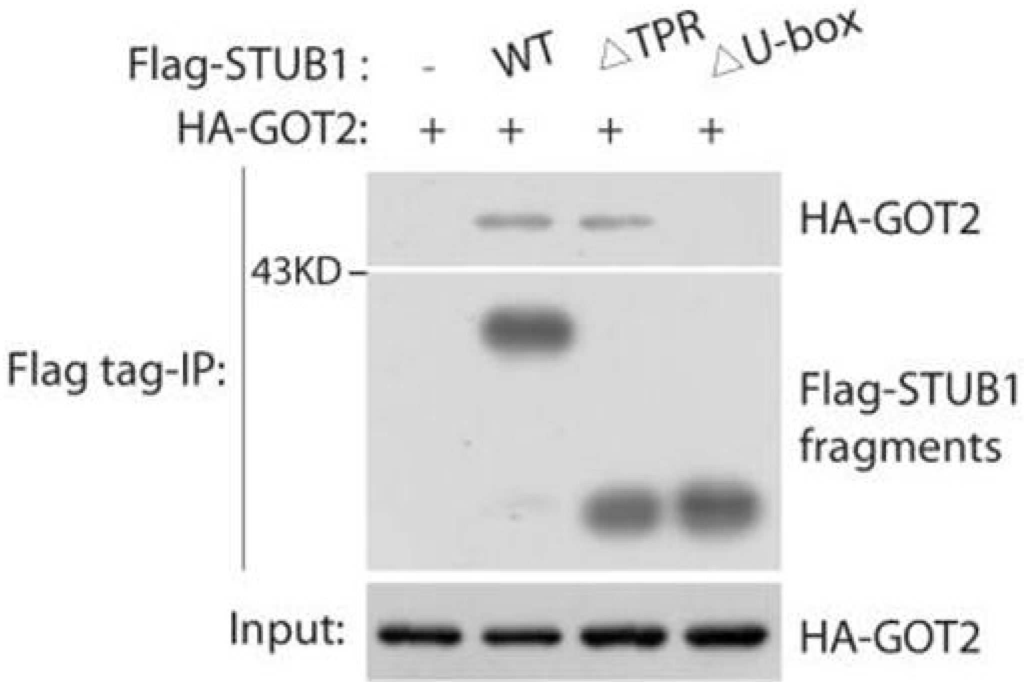
Flag-STUB1 :
-
WT
△TPR
△U-box
HA-GOT2:
+
+
+
+
HA-GOT2
43KD
Flag tag-IP:
Flag-STUB1
fragments
Input:
HA-GOT2

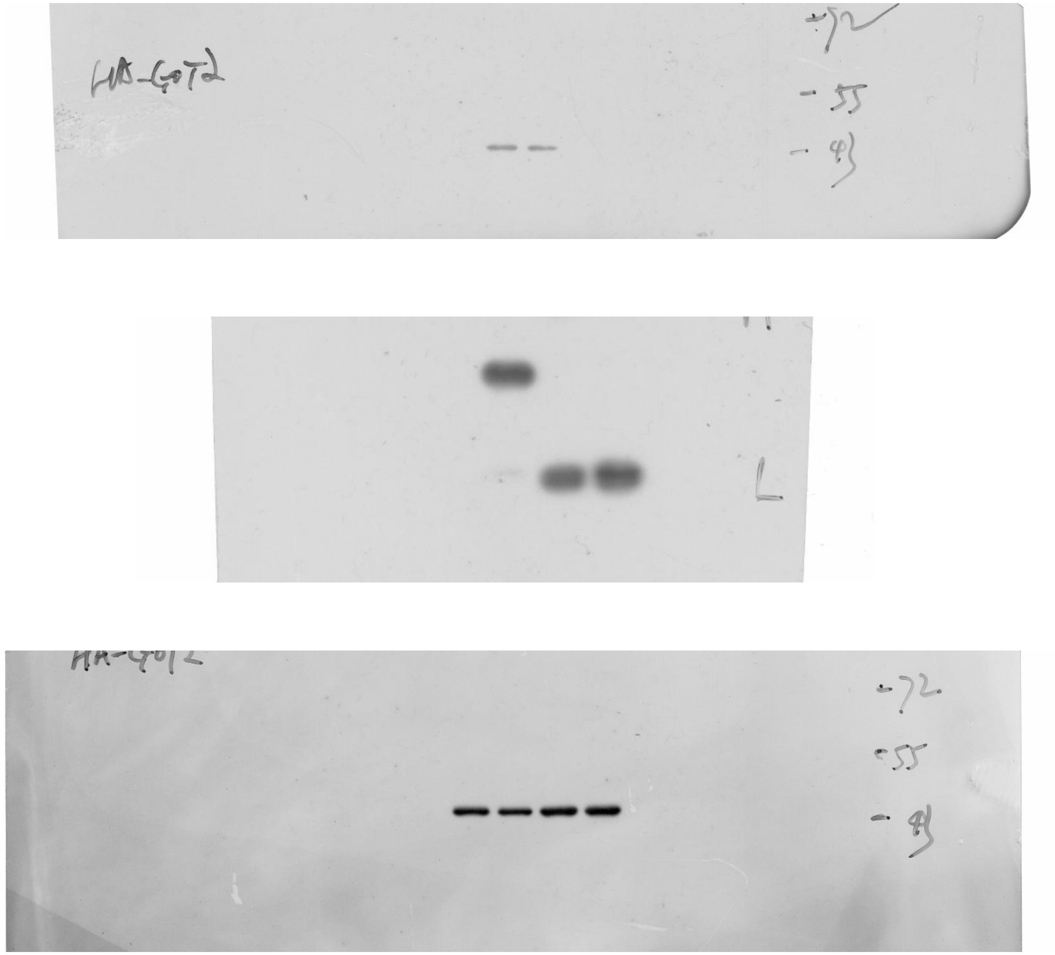
HA-GOT2
72
55

Figure5

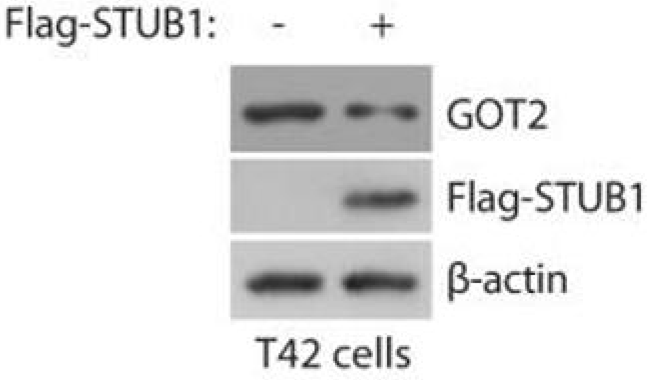

Flag-STUB1: - +
GOT2
Flag-STUB1
β-actin
T42 cells

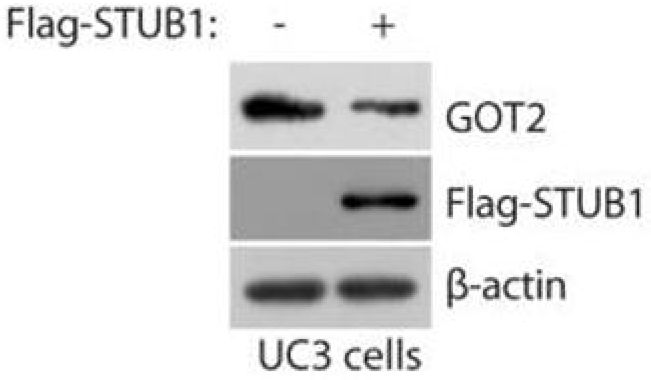

Flag-STUB1: - +
GOT2
Flag-STUB1
β-actin
UC3 cells

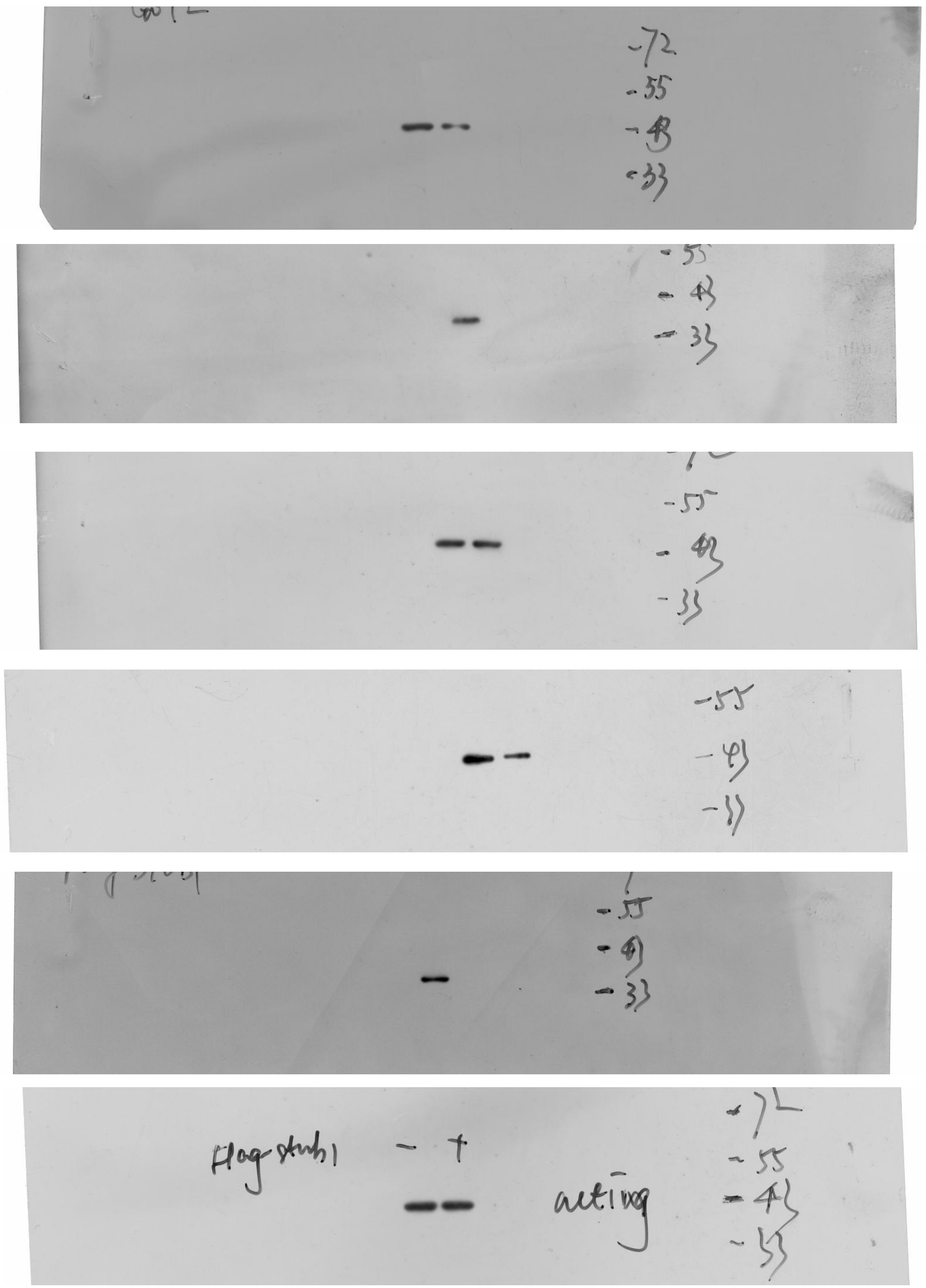

72
55
43
33

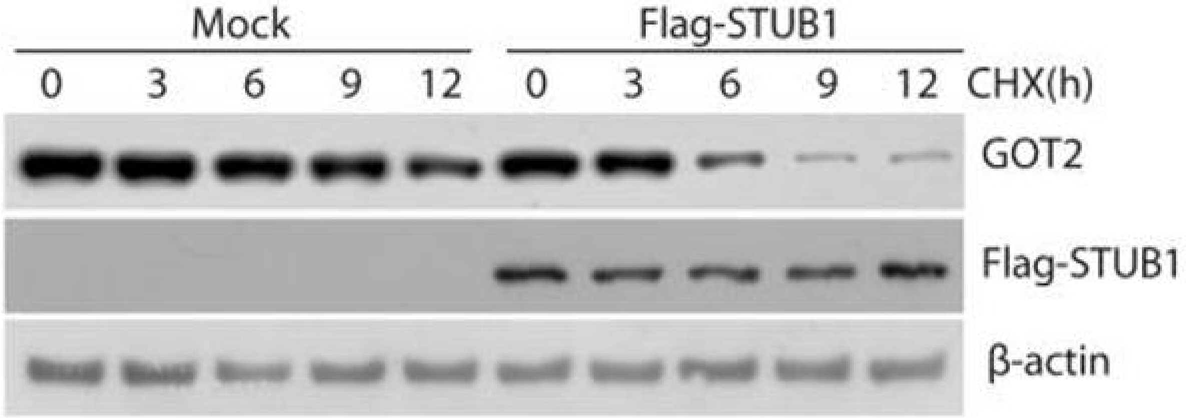
Mock
Flag-STUB1
0 3 6 9 12 0 3 6 9 12 CHX(h)
GOT2
Flag-STUB1
β-actin

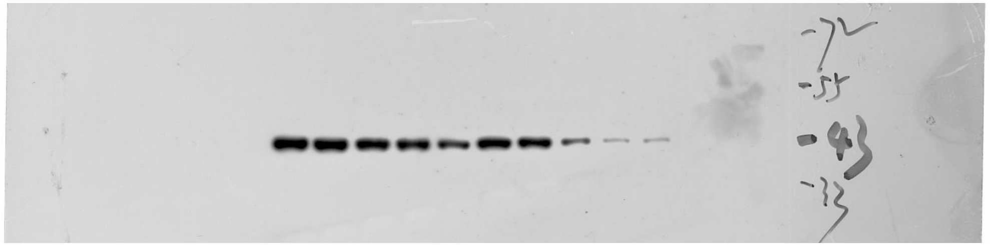
72
55
43
33

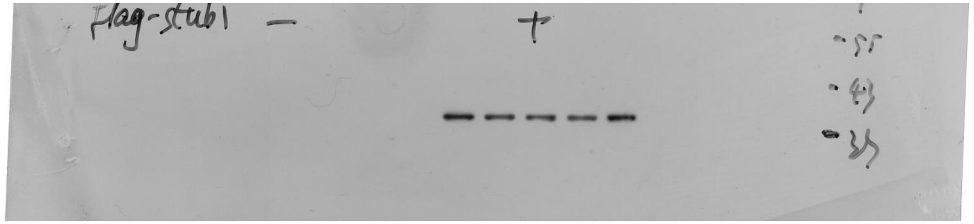
Flag-stub1 − +
55
43
33

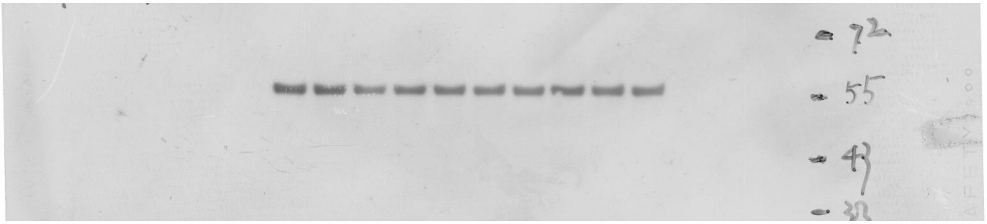
72
55
43

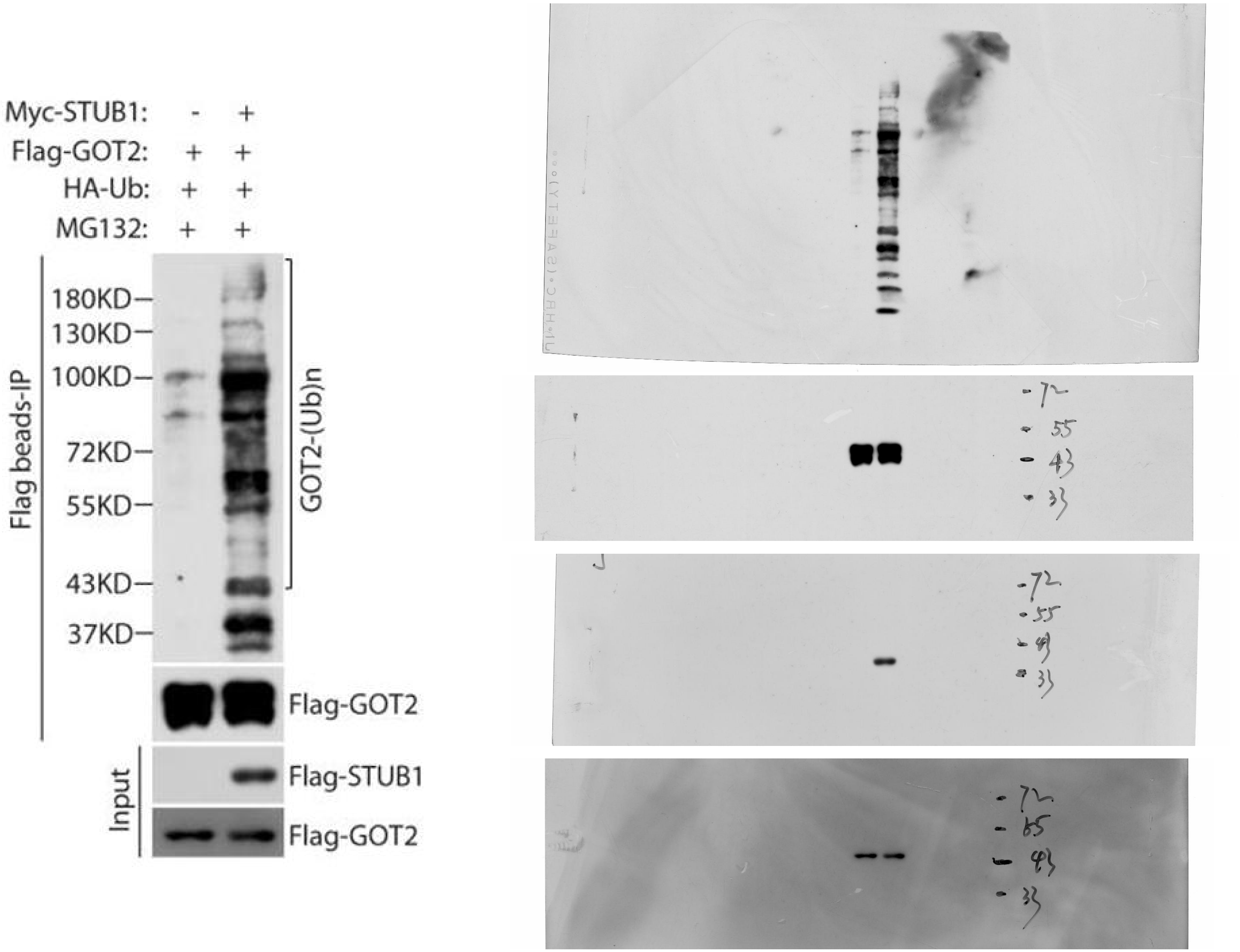

Myc-STUB1: - +
Flag-GOT2: + +
HA-Ub: + +
MG132: + +
Flag beads-IP
180KD
130KD
100KD
72KD
55KD
43KD
37KD
GOT2-(Ub)n
Flag-GOT2
Input
Flag-STUB1
Flag-GOT2
72
55
43
33

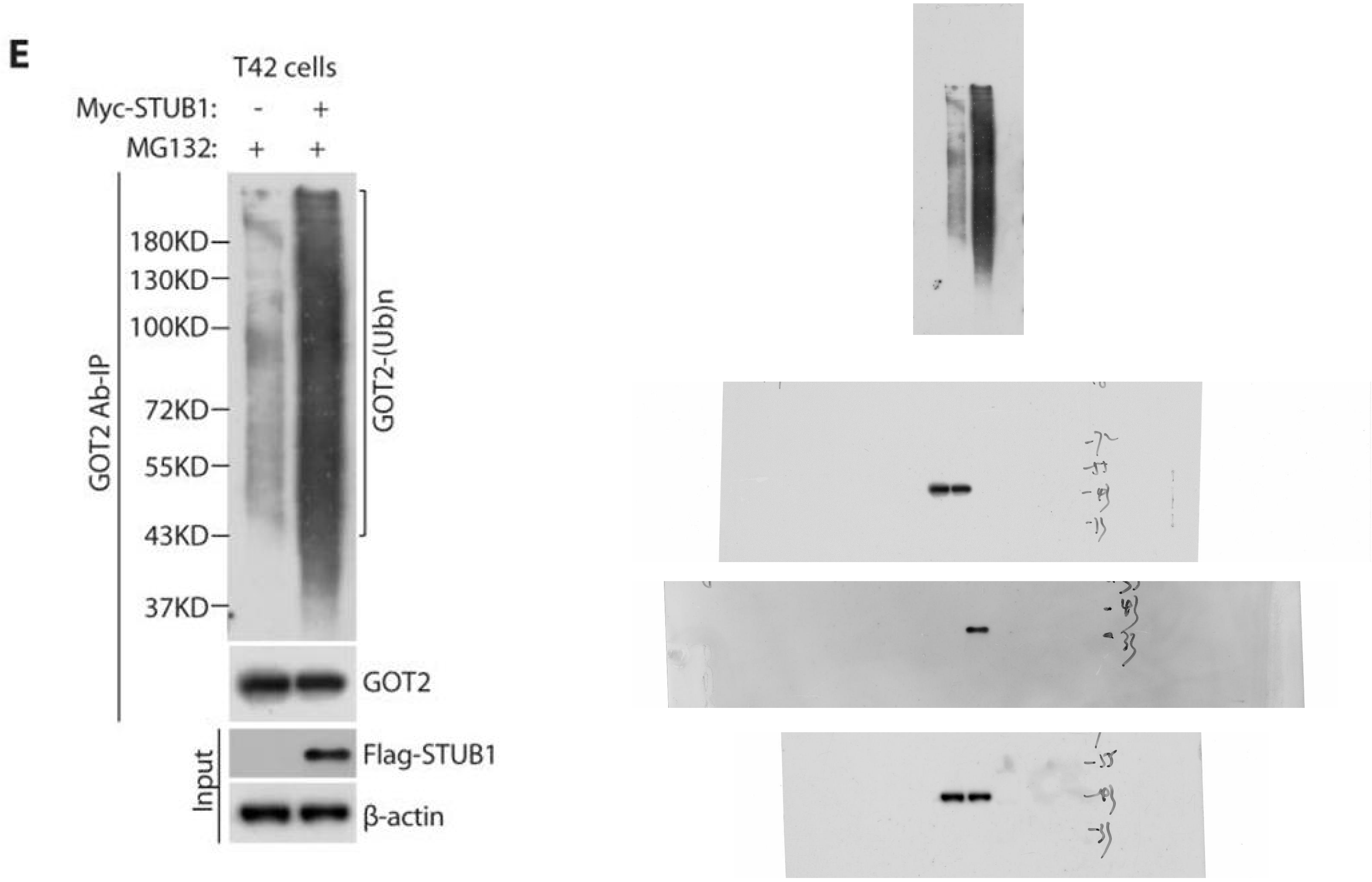
E
T42 cells
Myc-STUB1: - +
MG132: + +
GOT2 Ab-IP
180KD
130KD
100KD
72KD
55KD
43KD
37KD
GOT2-(Ub)n
GOT2
Input
Flag-STUB1
β-actin

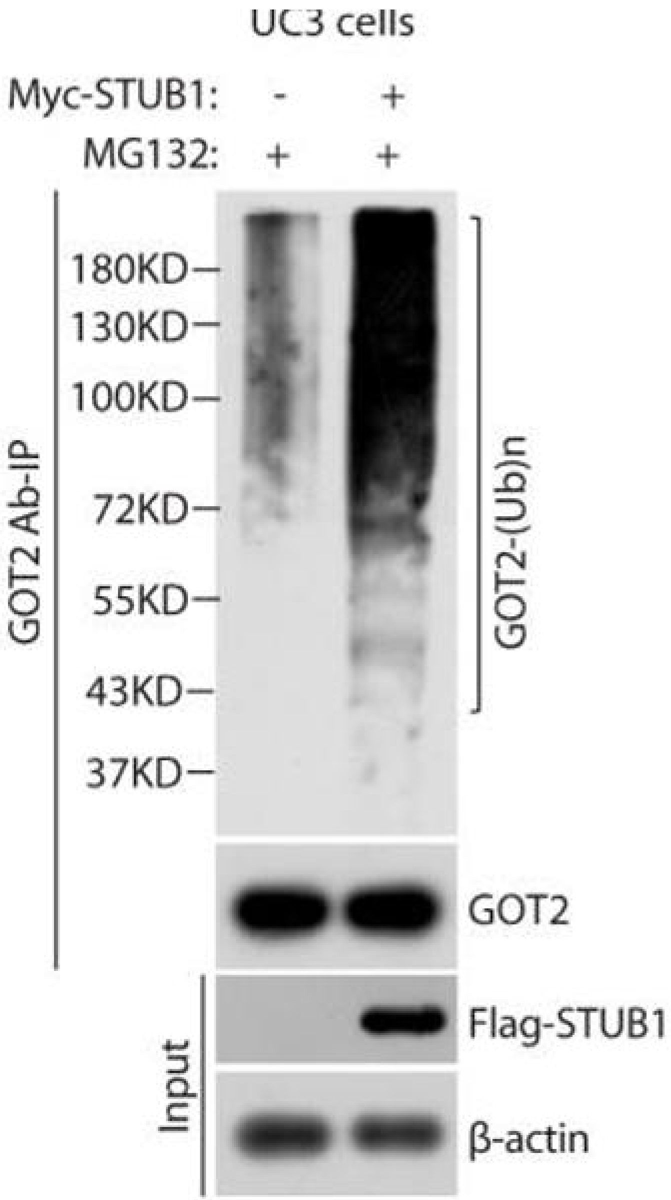

UC3 cells
Myc-STUB1: - +
MG132: + +
180KD
130KD
100KD
72KD
55KD
43KD
37KD
GOT2 Ab-IP
GOT2-(Ub)n
GOT2
Flag-STUB1
β-actin
Input

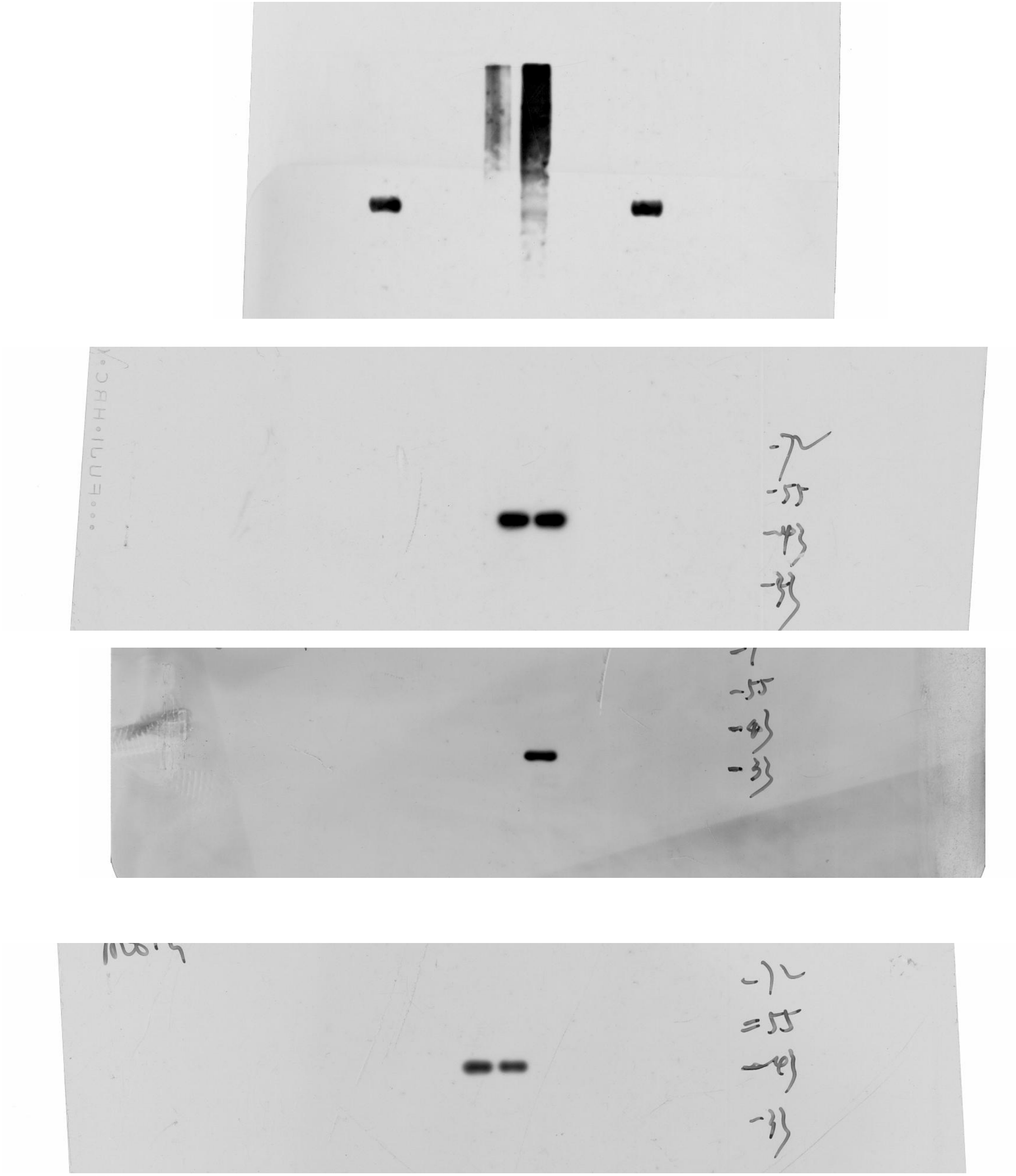

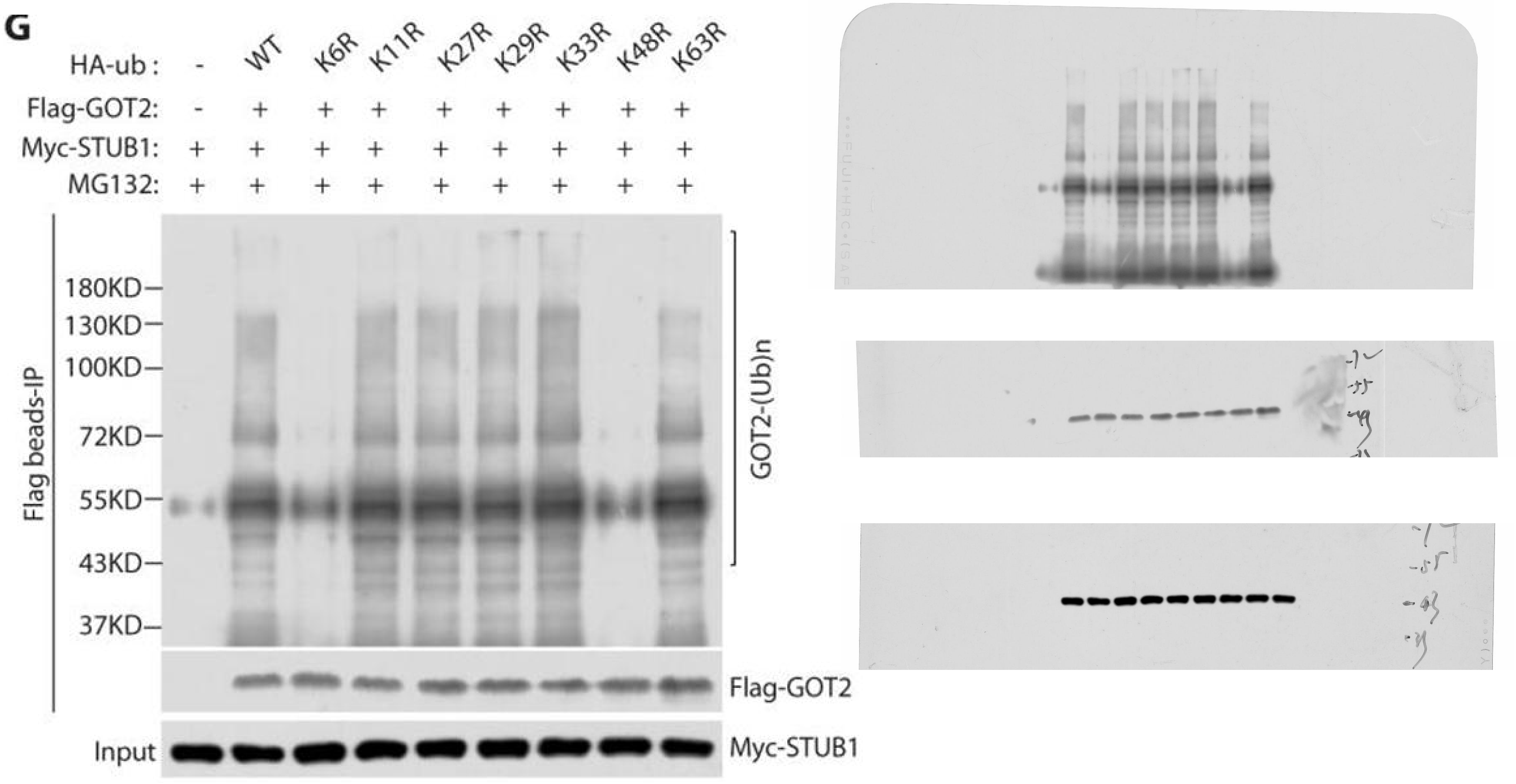
G
HA-ub :
-
WT
K6R
K11R
K27R
K29R
K33R
K48R
K63R
Flag-GOT2:
Myc-STUB1:
MG132:
180KD
130KD
100KD
72KD
55KD
43KD
37KD
Flag beads-IP
GOT2-(Ub)n
Flag-GOT2
Input
Myc-STUB1

I

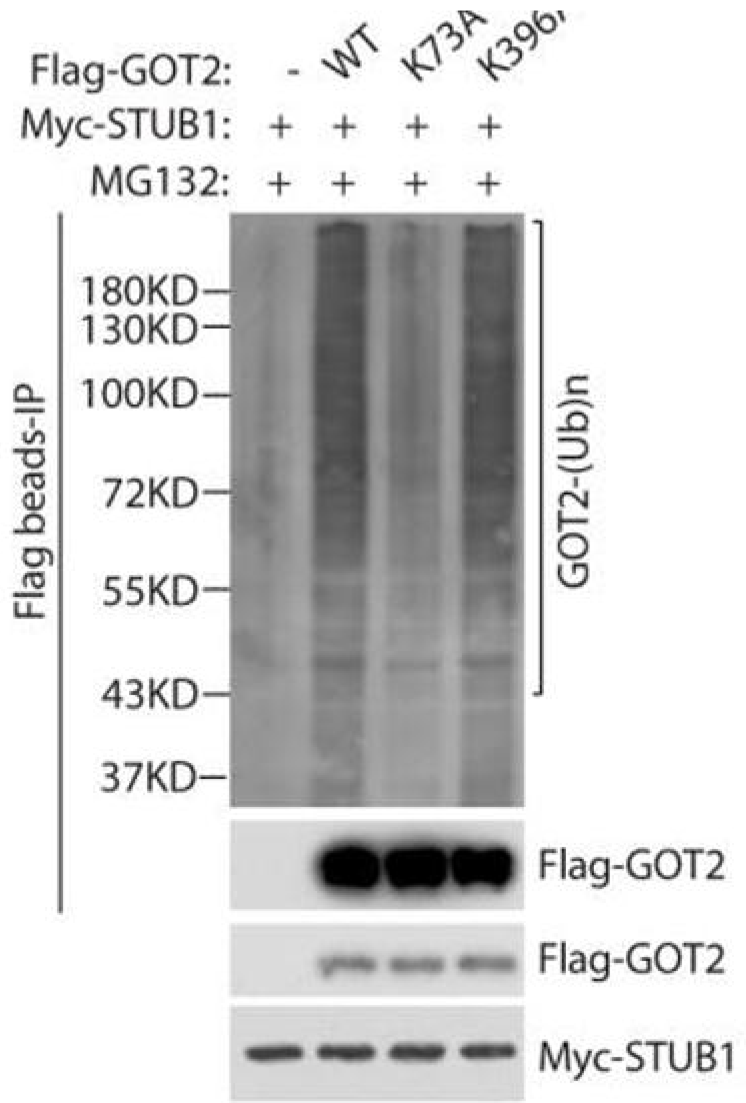

Flag-GOT2: - WT K73A K396
Myc-STUB1: + + + +
MG132: + + + +
Flag beads-IP
180KD
130KD
100KD
72KD
55KD
43KD
37KD
GOT2-(Ub)n
Flag-GOT2
Flag-GOT2
Myc-STUB1

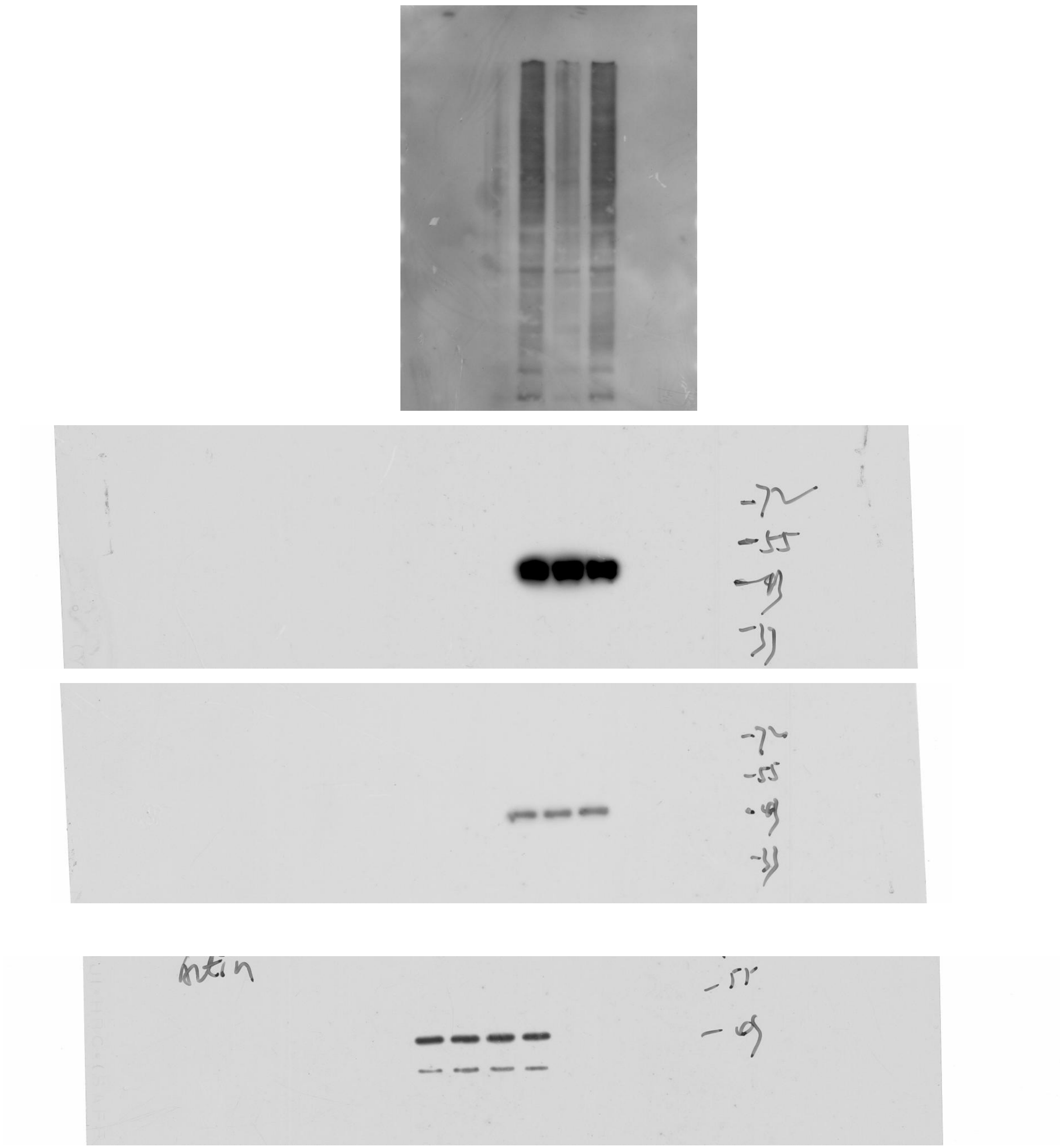

-72
-55
-72
-55

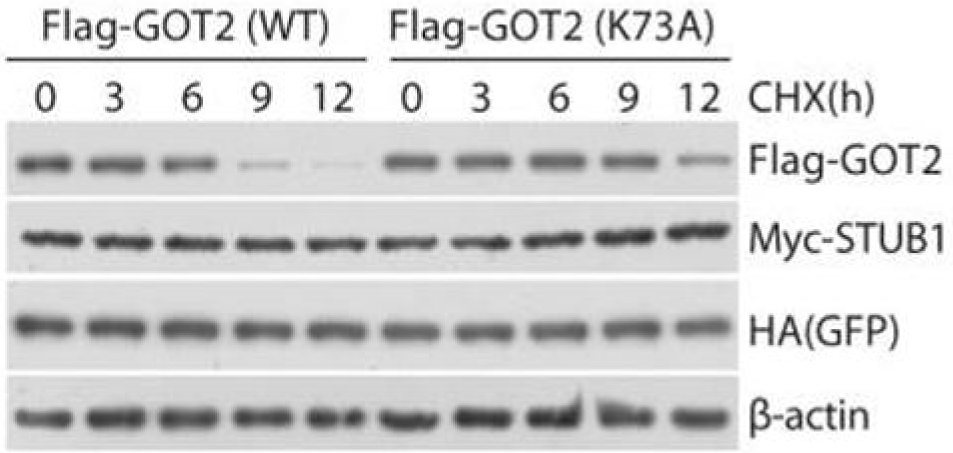
Flag-GOT2 (WT)
Flag-GOT2 (K73A)
0 3 6 9 12 0 3 6 9 12 CHX(h)
Flag-GOT2
Myc-STUB1
HA(GFP)
β-actin

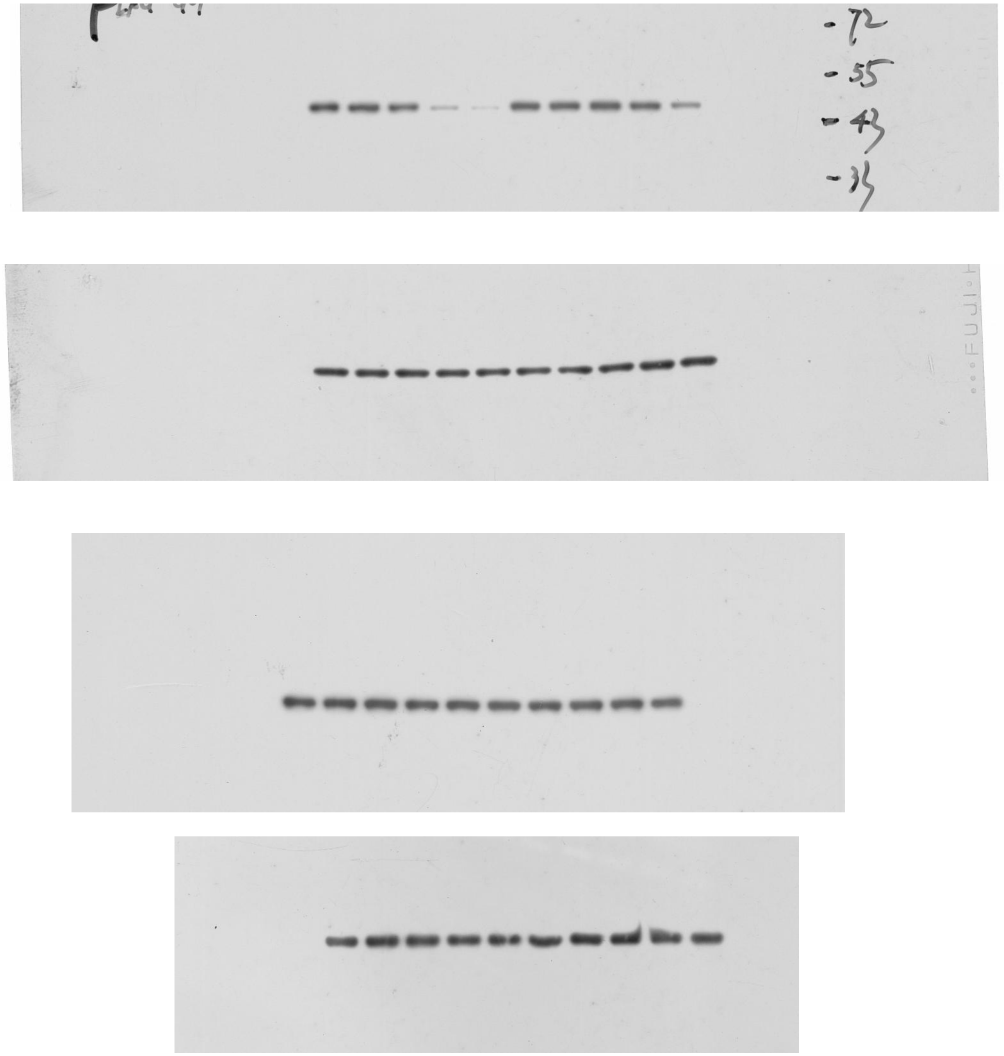

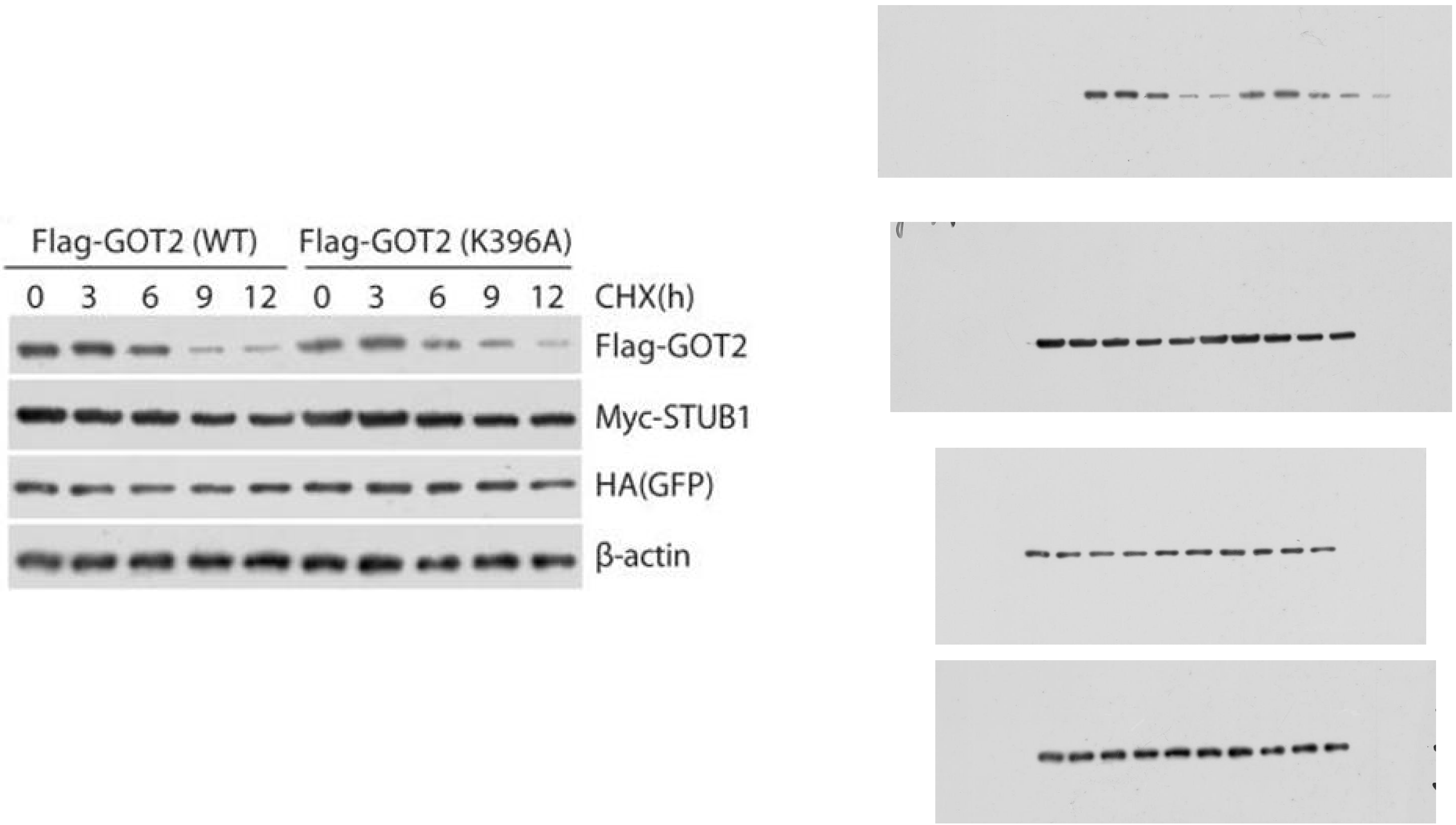

Flag-GOT2 (WT)
Flag-GOT2 (K396A)
0 3 6 9 12 0 3 6 9 12
CHX(h)
Flag-GOT2
Myc-STUB1
HA(GFP)
β-actin

Figure6

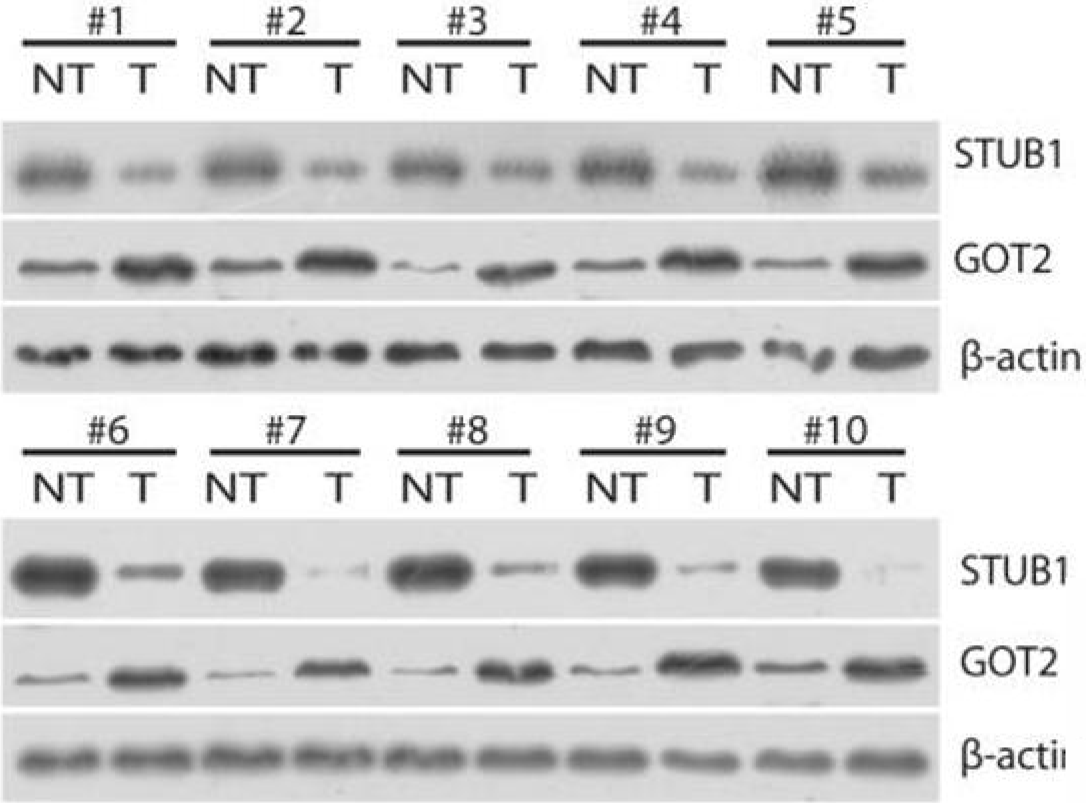
#1 #2 #3 #4 #5
NT T NT T NT T NT T NT T
STUB1
GOT2
β-actin
#6 #7 #8 #9 #10
NT T NT T NT T NT T NT T
STUB1
GOT2
β-acti

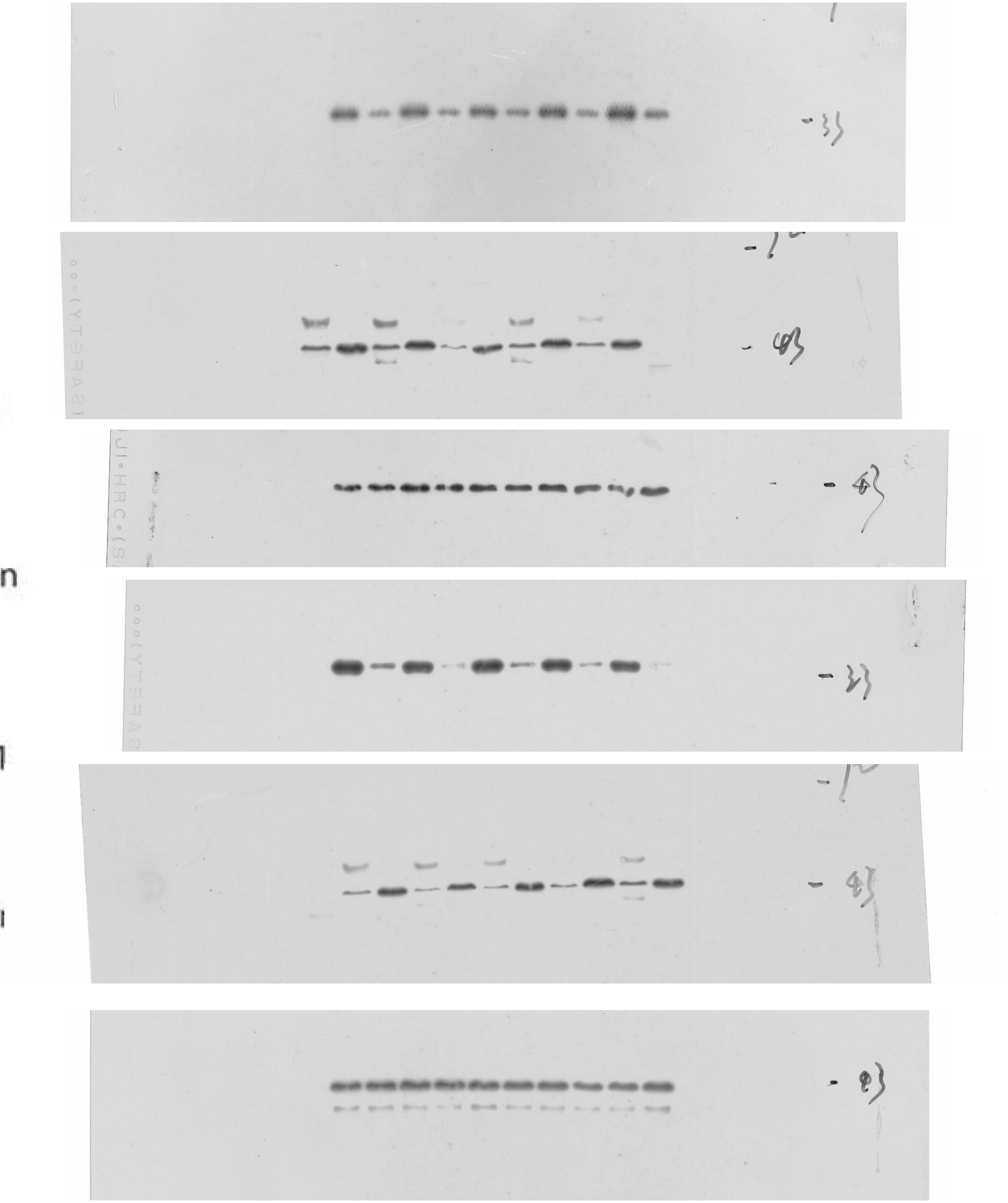

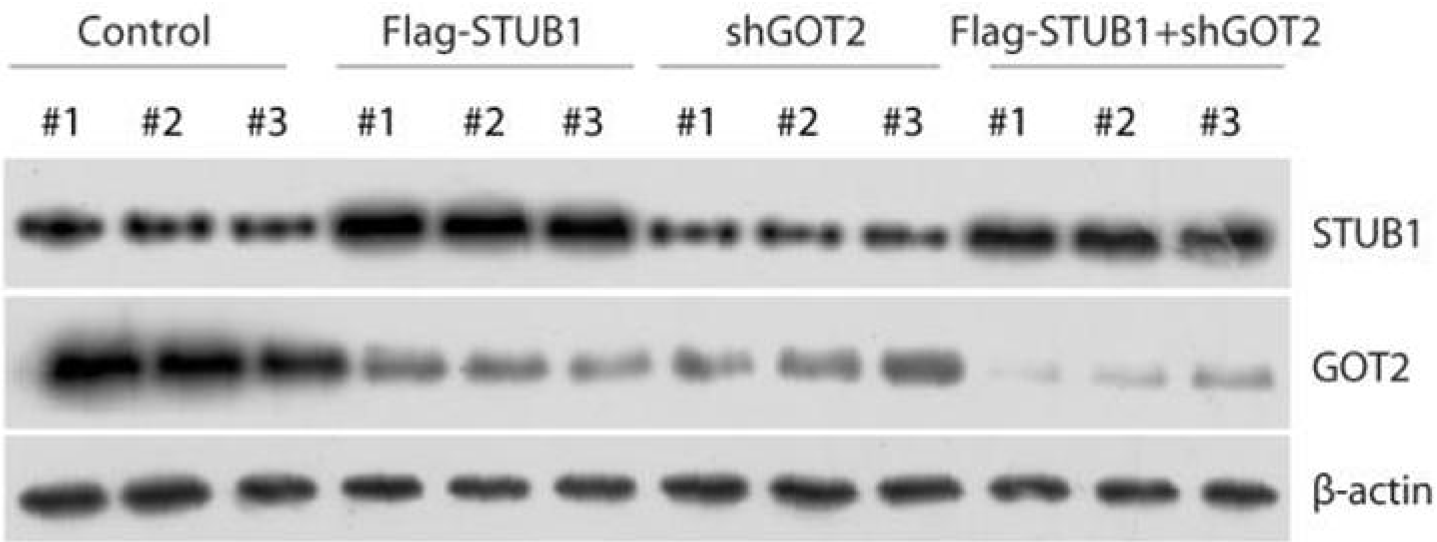
Control
Flag-STUB1
shGOT2
Flag-STUB1+shGOT2
#1
#2
#3
#1
#2
#3
#1
#2
#3
#1
#2
#3
STUB1
GOT2
β-actin

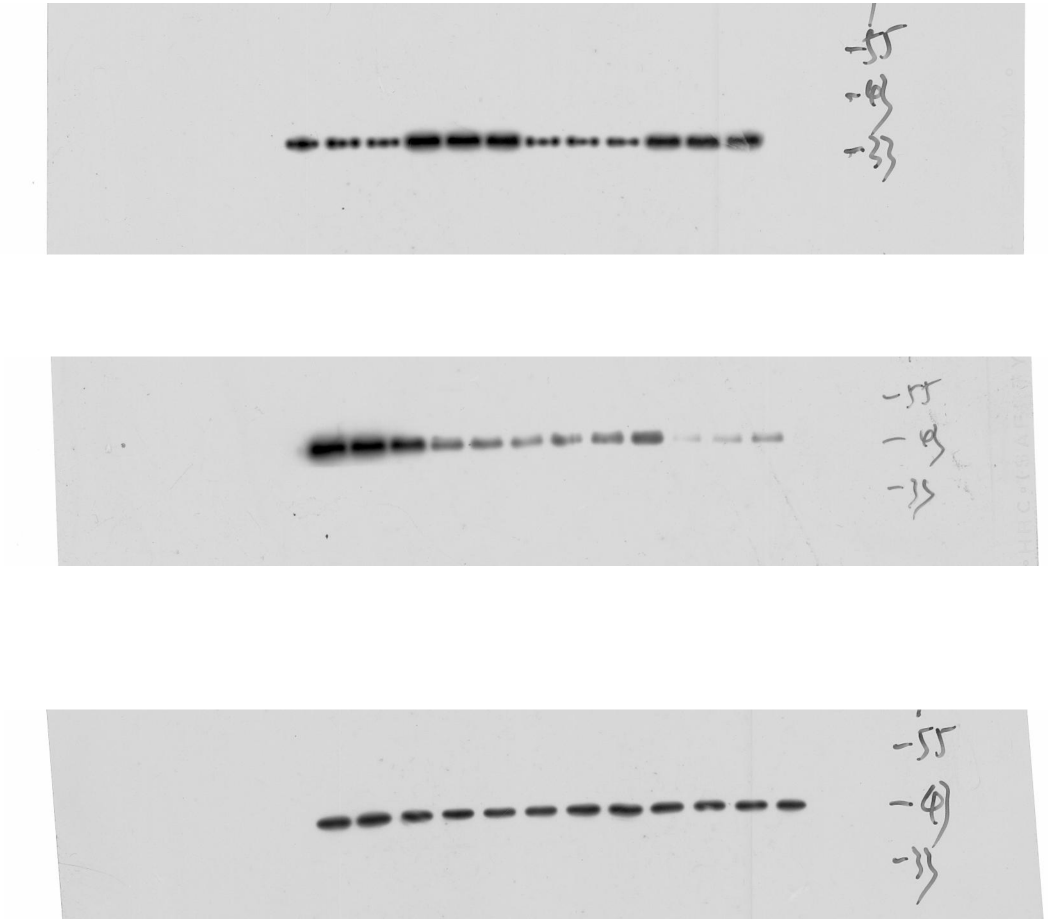

Figure7

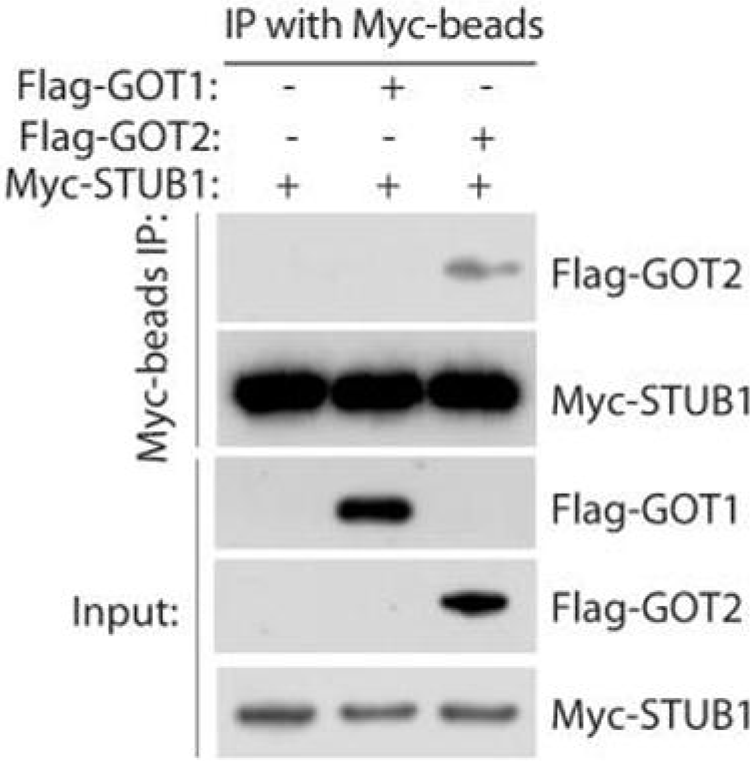
IP with Myc-beads
Flag-GOT1: - + -
Flag-GOT2: - - +
Myc-STUB1: + + +
Myc-beads IP:
Flag-GOT2
Myc-STUB1
Flag-GOT1
Input:
Flag-GOT2
Myc-STUB1

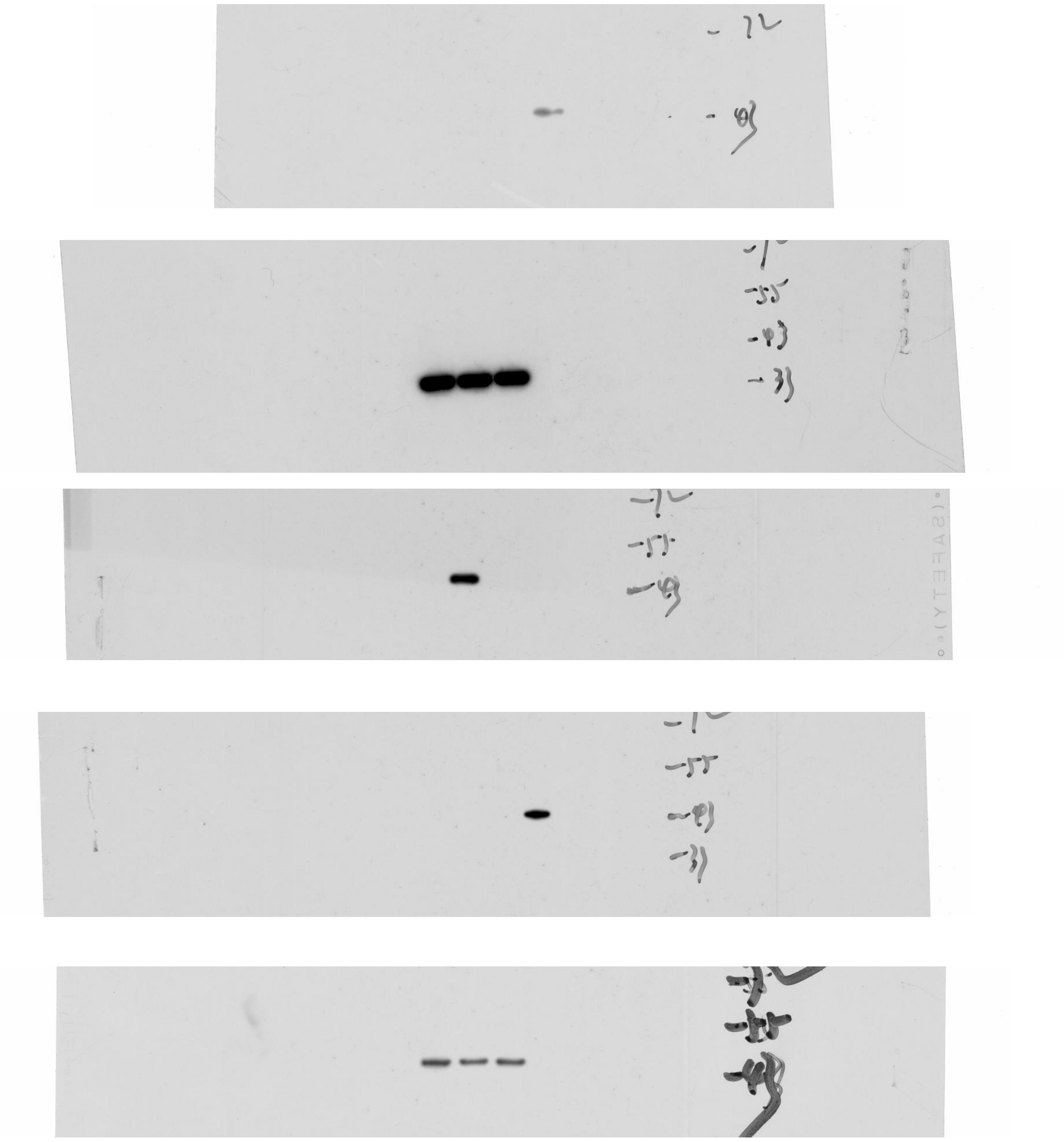

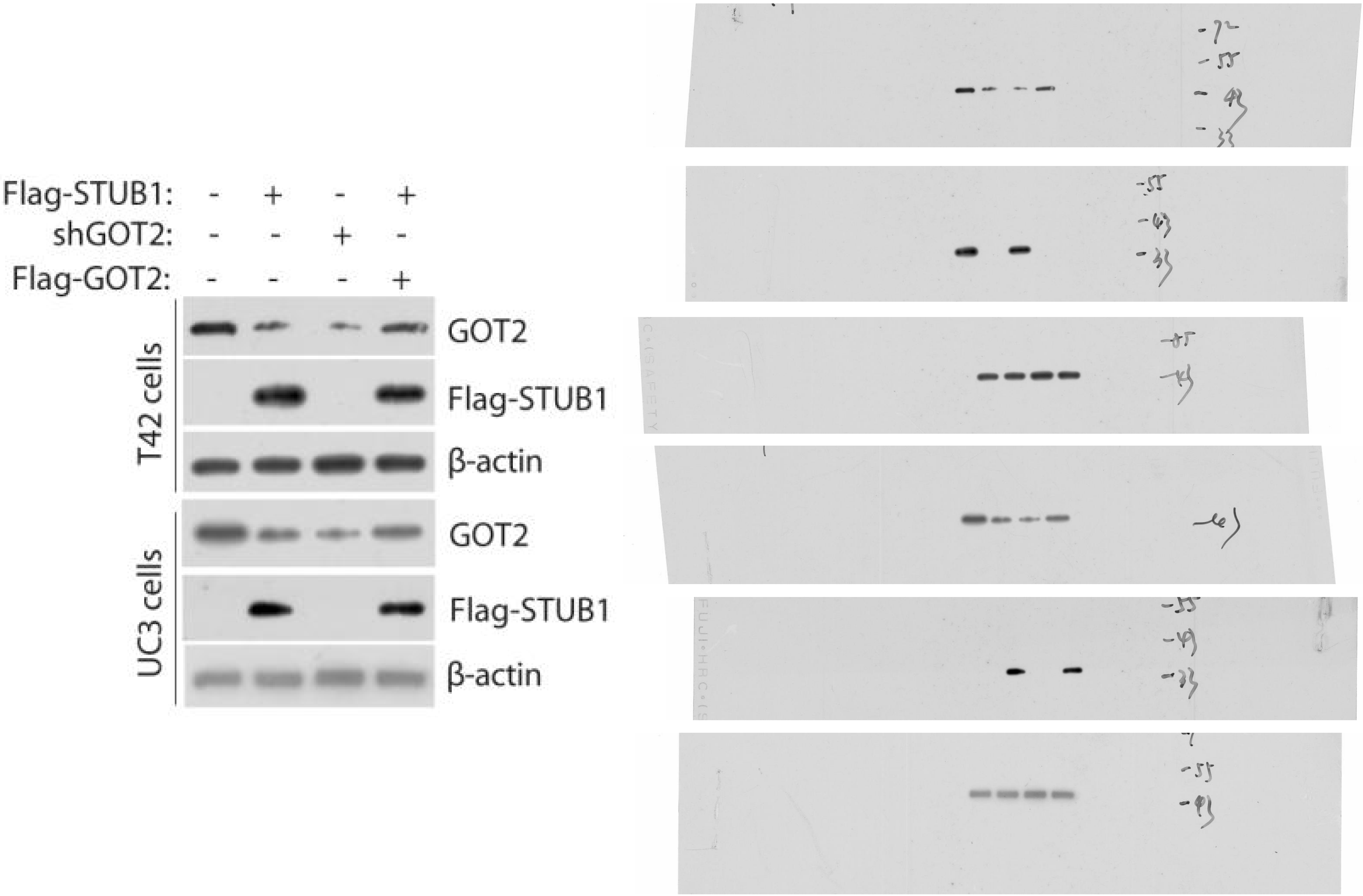
Flag-STUB1: - + - +
shGOT2: - - + -
Flag-GOT2: - - - +
T42 cells
GOT2
Flag-STUB1
β-actin
UC3 cells
GOT2
Flag-STUB1
β-actin

FIgure8

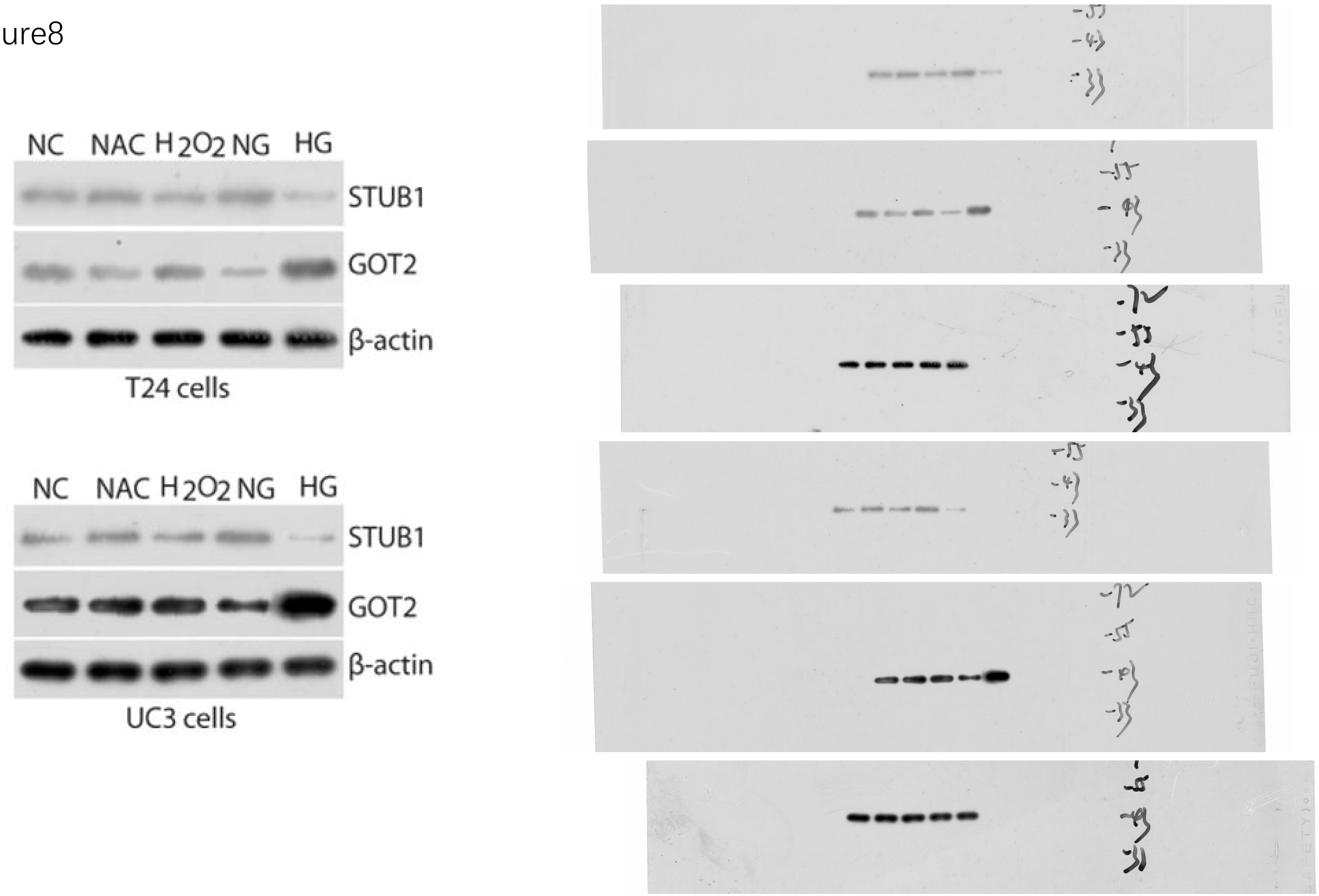

NC NAC H2O2 NG HG
STUB1
GOT2
β-actin
T24 cells
NC NAC H2O2 NG HG
STUB1
GOT2
β-actin
UC3 cells

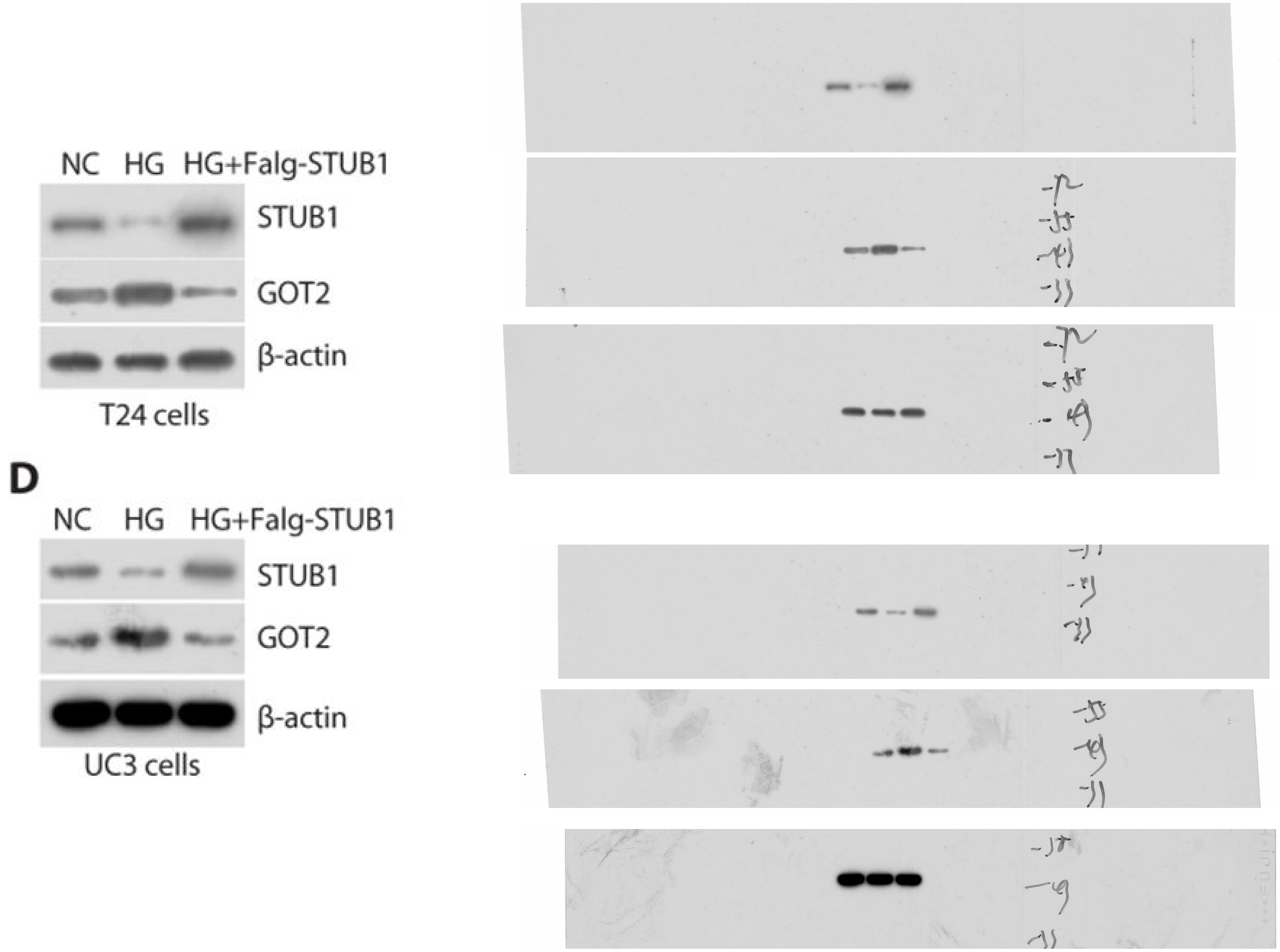
NC
HG
HG+Falg-STUB1
STUB1
GOT2
β-actin
T24 cells
D
NC
HG
HG+Falg-STUB1
STUB1
GOT2
β-actin
UC3 cells

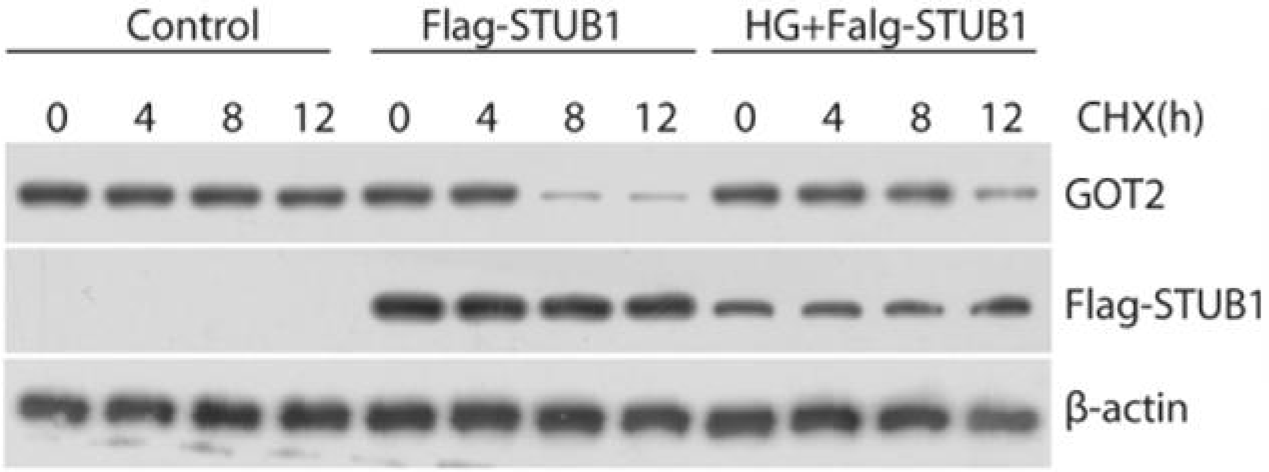
Control
Flag-STUB1
HG+Falg-STUB1
0 4 8 12 0 4 8 12 0 4 8 12
CHX(h)
GOT2
Flag-STUB1
β-actin

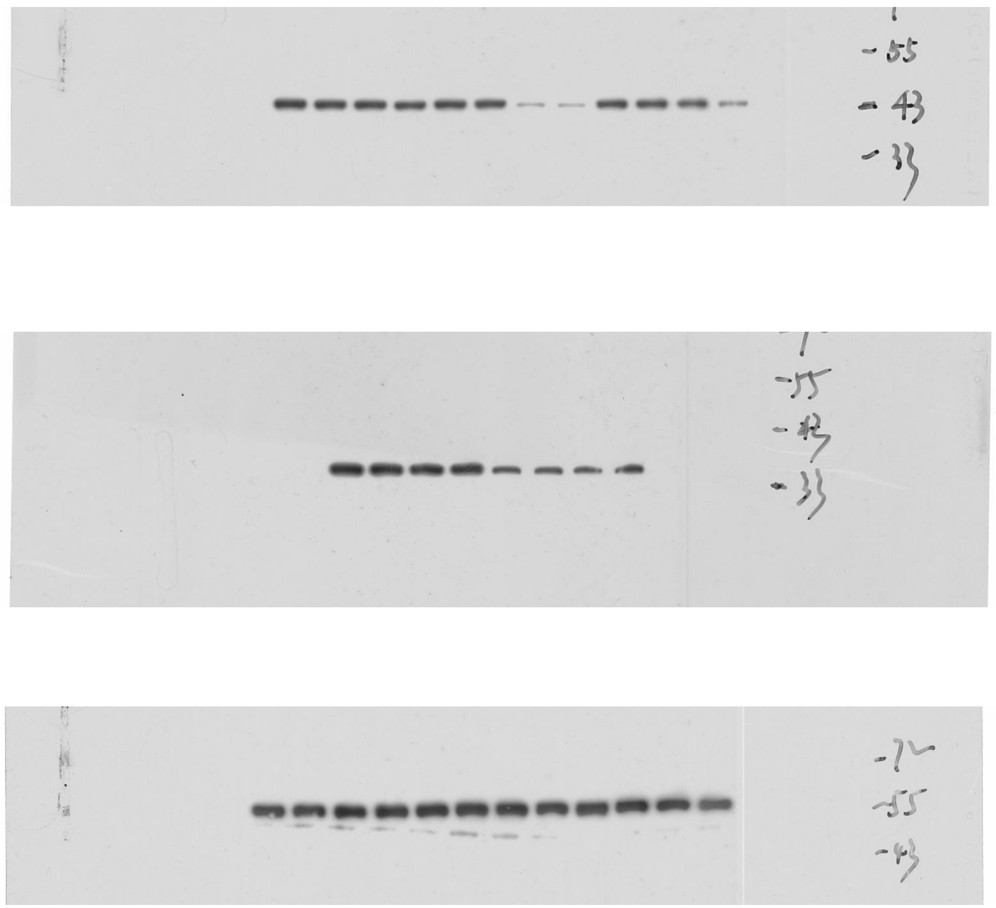

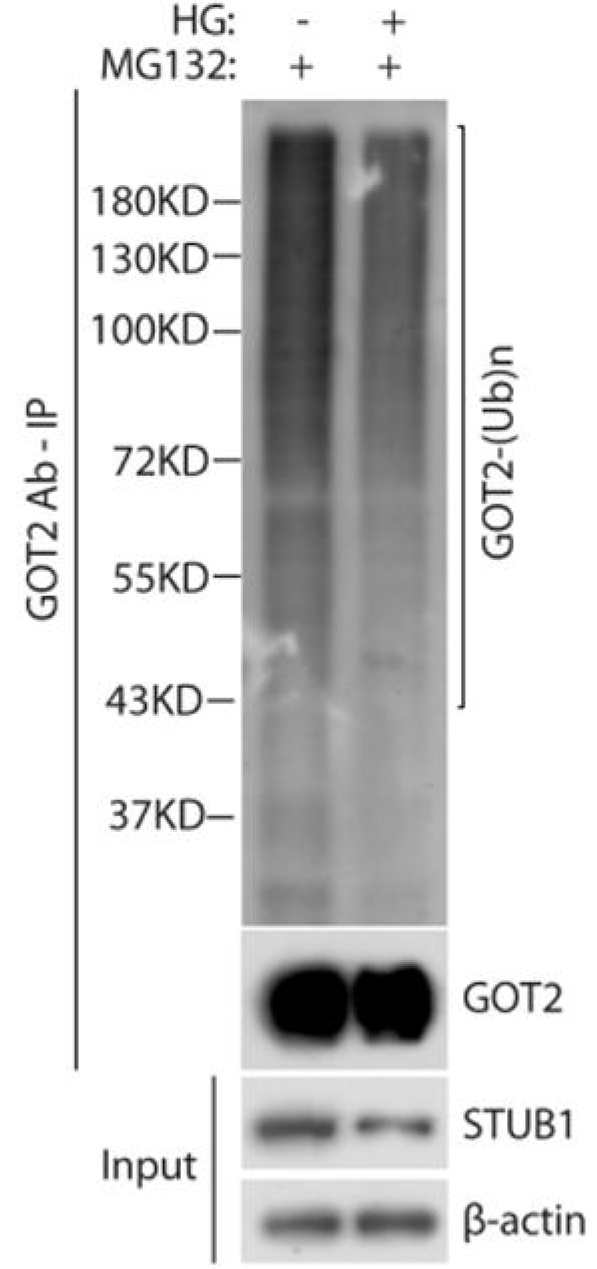
HG: - +
MG132: + +
GOT2 Ab - IP
180KD
130KD
100KD
72KD
55KD
43KD
37KD
GOT2-(Ub)n
GOT2
Input
STUB1
β-actin

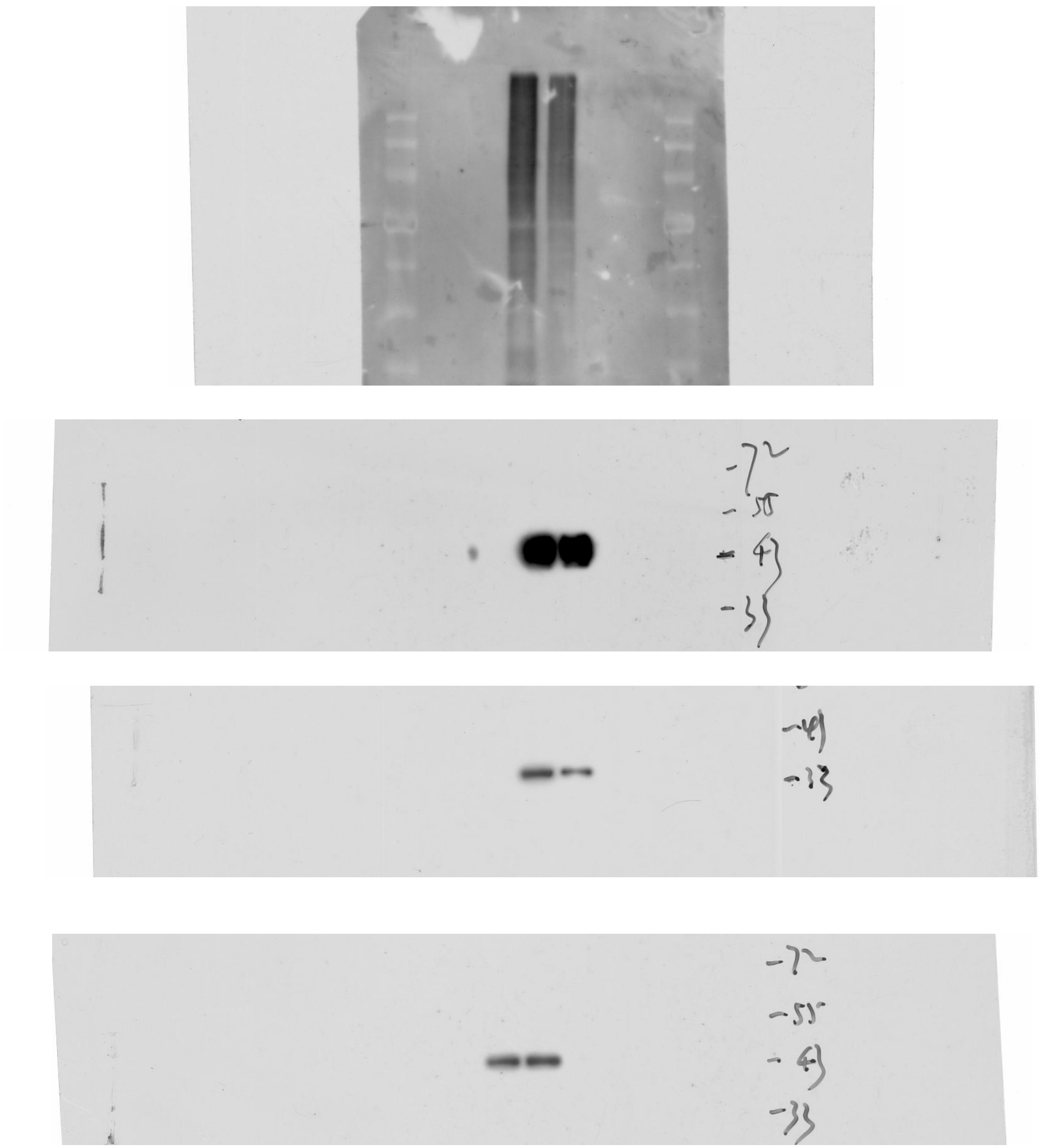

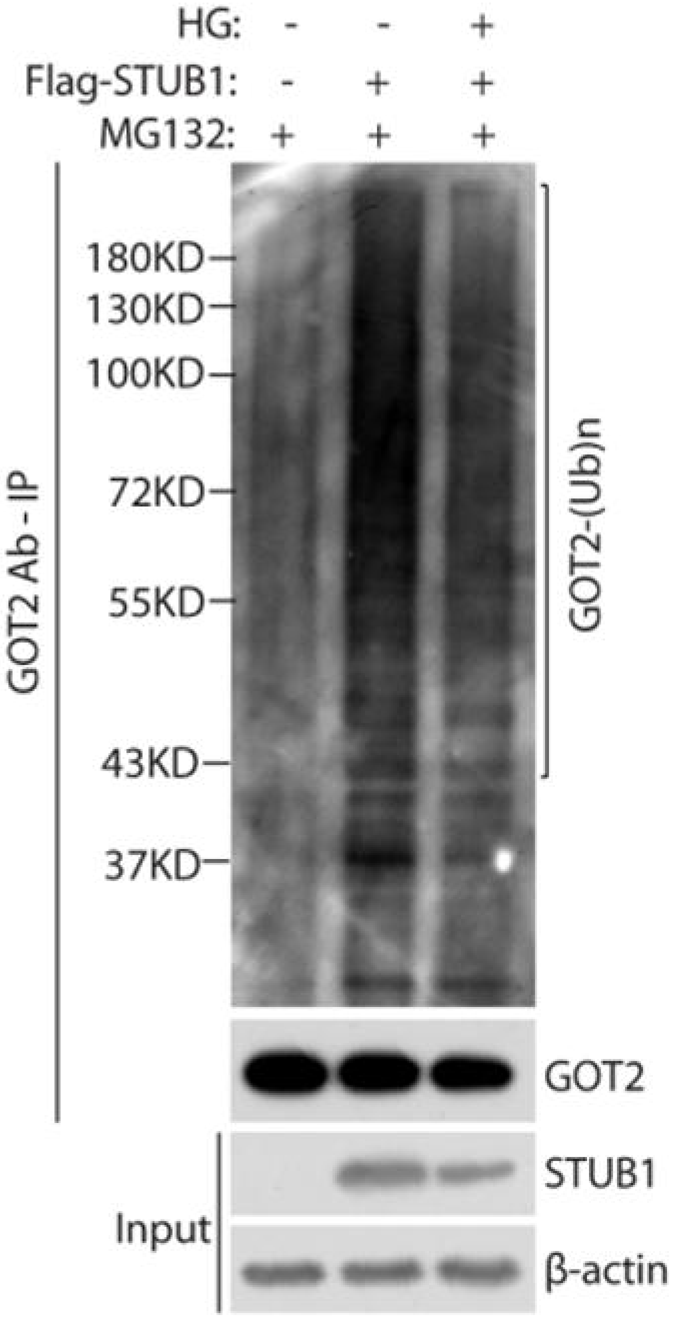
HG: - - +
Flag-STUB1: - + +
MG132: + + +
GOT2 Ab - IP
180KD
130KD
100KD
72KD
55KD
43KD
37KD
GOT2-(Ub)n
GOT2
Input
STUB1
β-actin

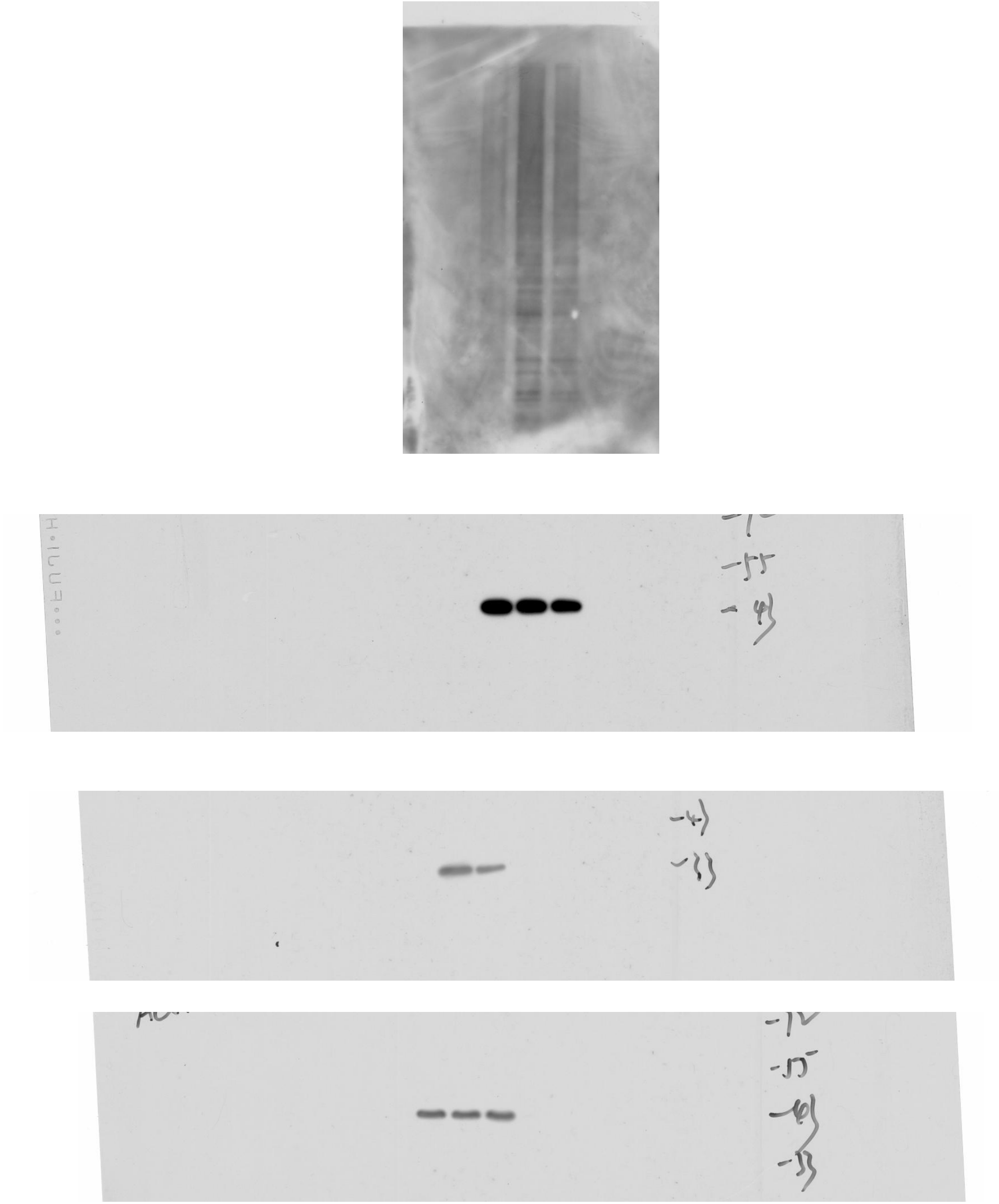

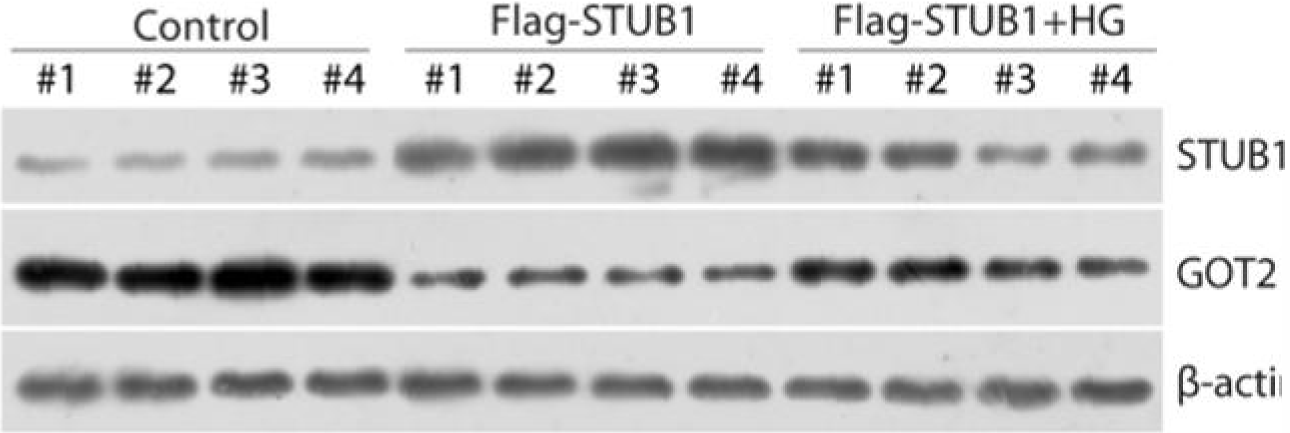
Control
Flag-STUB1
Flag-STUB1+HG
#1 #2 #3 #4 #1 #2 #3 #4 #1 #2 #3 #4
STUB1
GOT2
β-acti

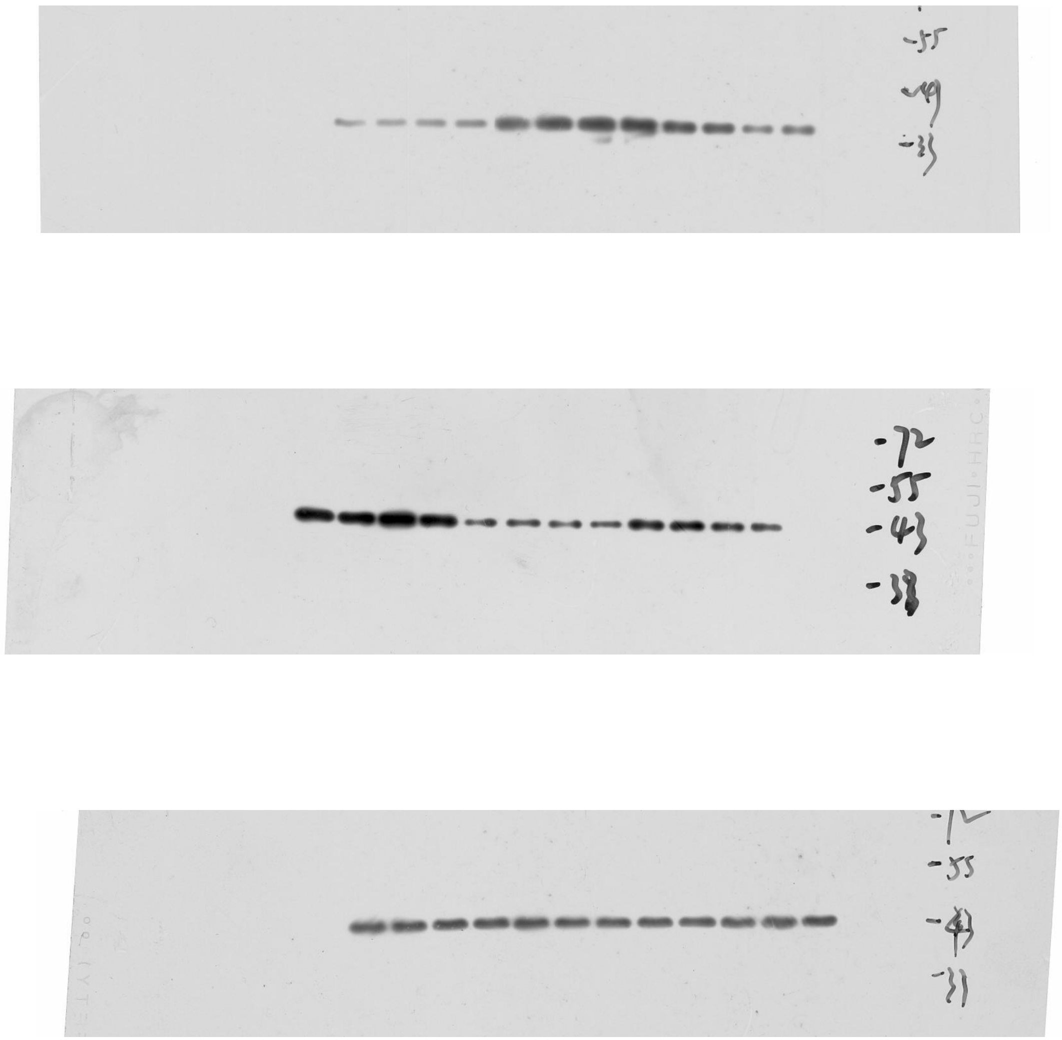

Figure.S2

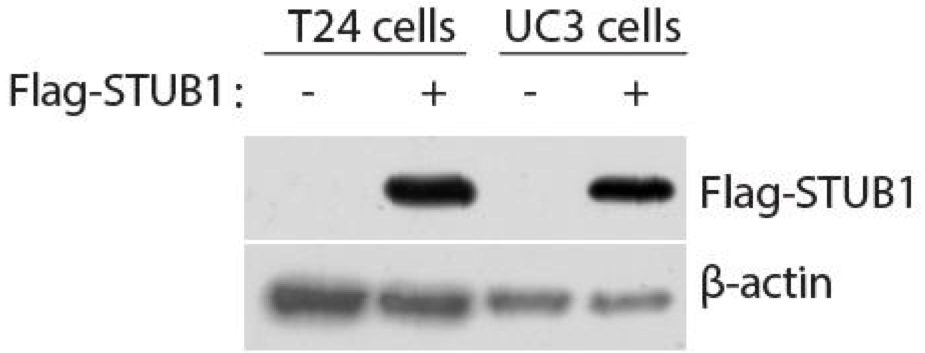
T24 cells
UC3 cells
Flag-STUB1 : - + - +
Flag-STUB1
β-actin

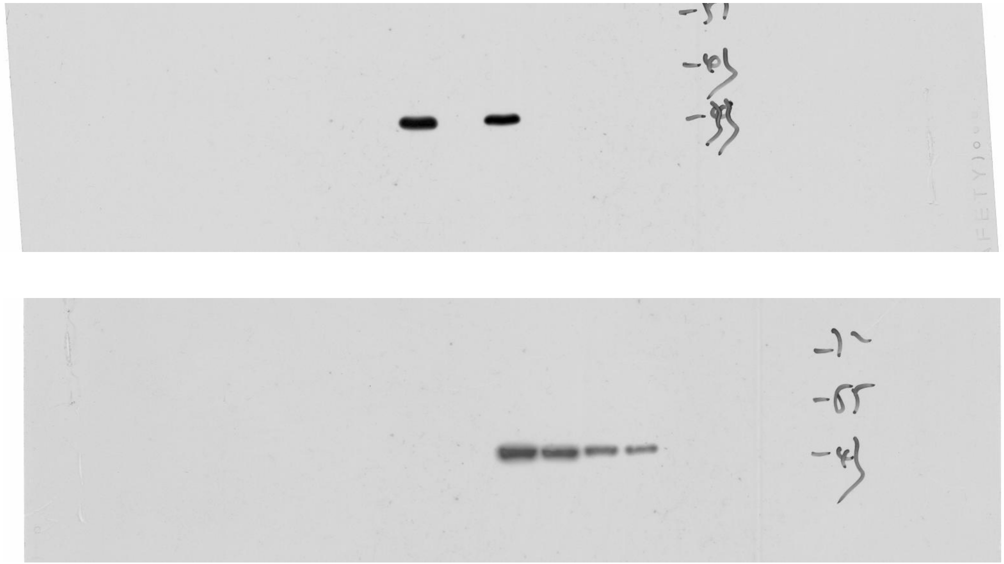

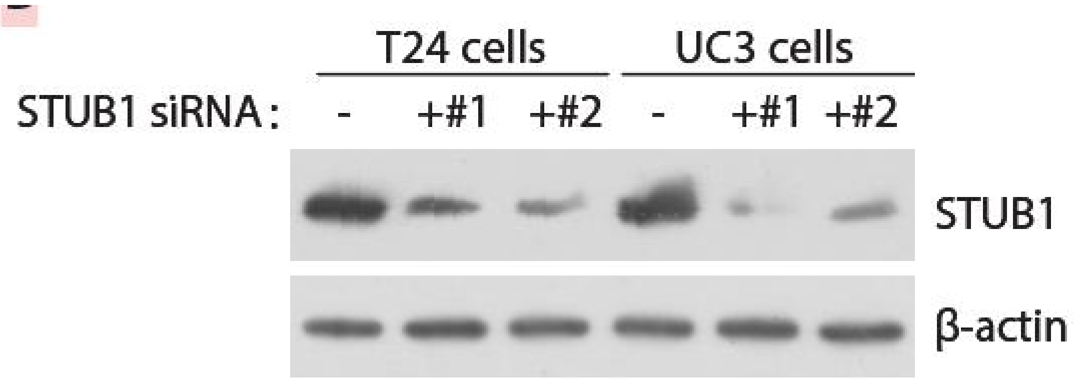
T24 cells
UC3 cells
STUB1 siRNA : - +#1 +#2 - +#1 +#2
STUB1
β-actin

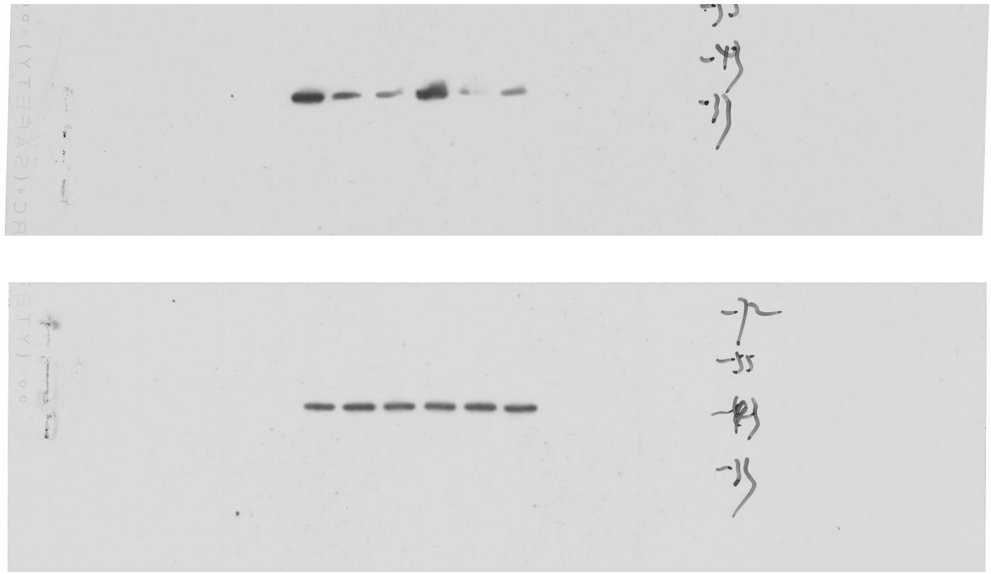

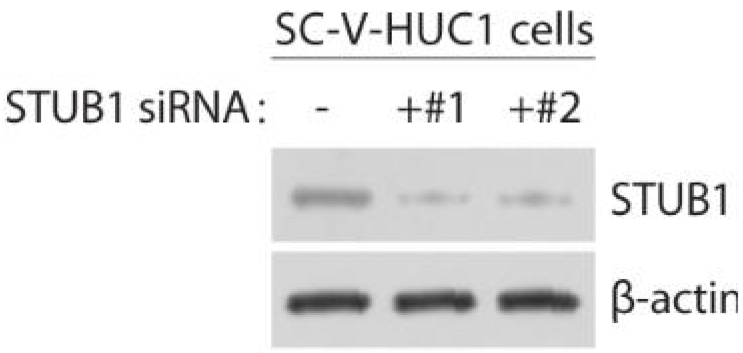
SC-V-HUC1 cells
STUB1 siRNA : - +#1 +#2
STUB1
β-actin

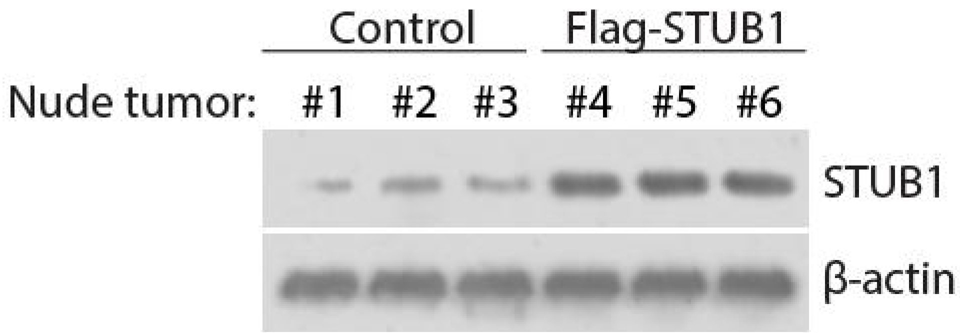
Control
Flag-STUB1
Nude tumor: #1 #2 #3 #4 #5 #6
STUB1
β-actin

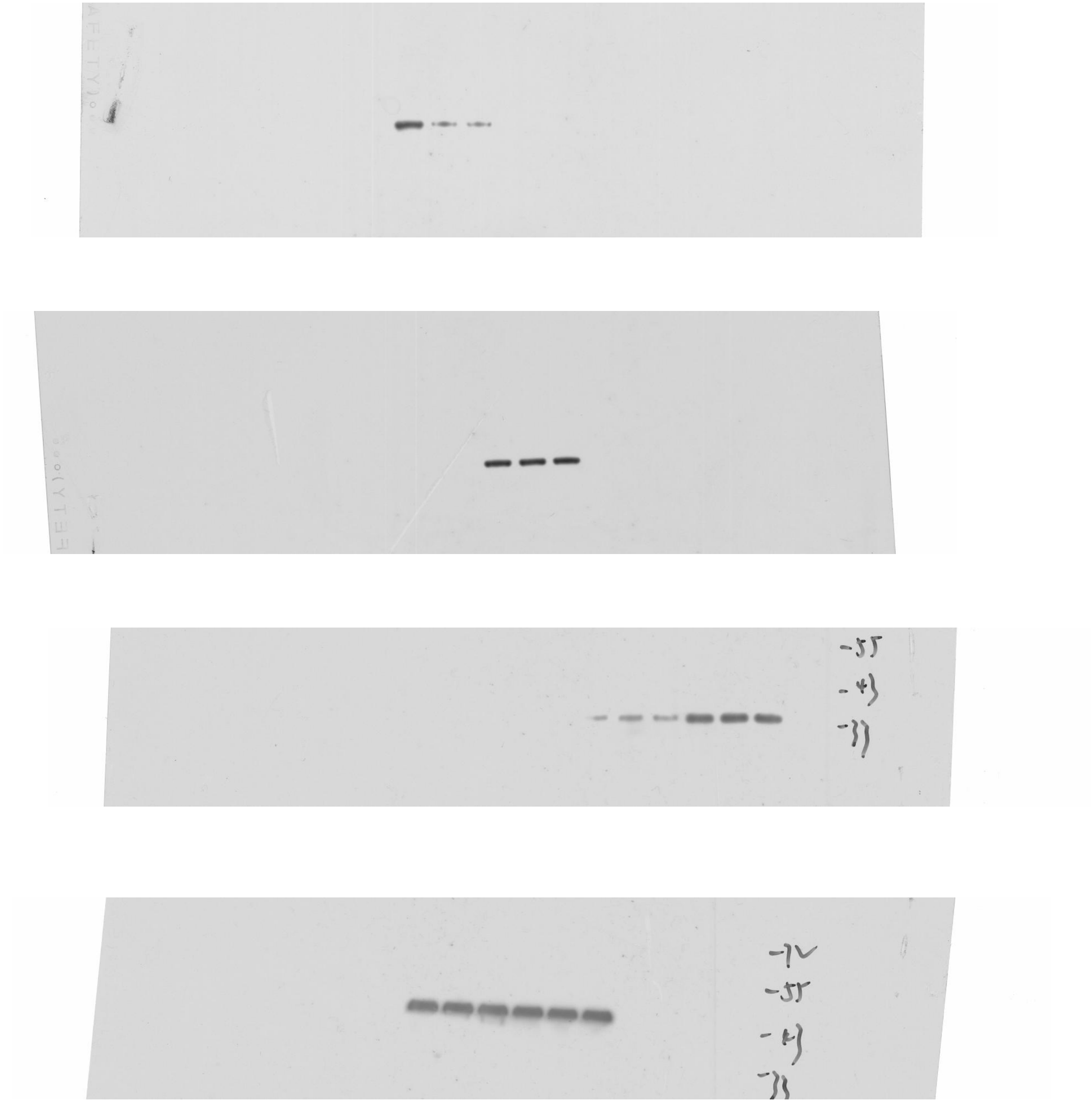

Figure.S4

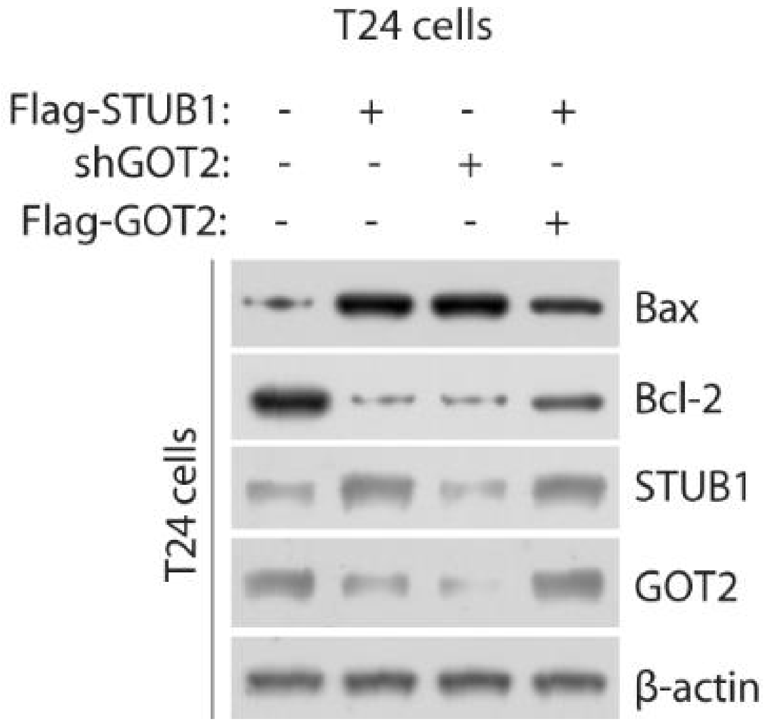
T24 cells
Flag-STUB1: - + - +
shGOT2: - - + -
Flag-GOT2: - - - +
T24 cells
Bax
Bcl-2
STUB1
GOT2
β-actin

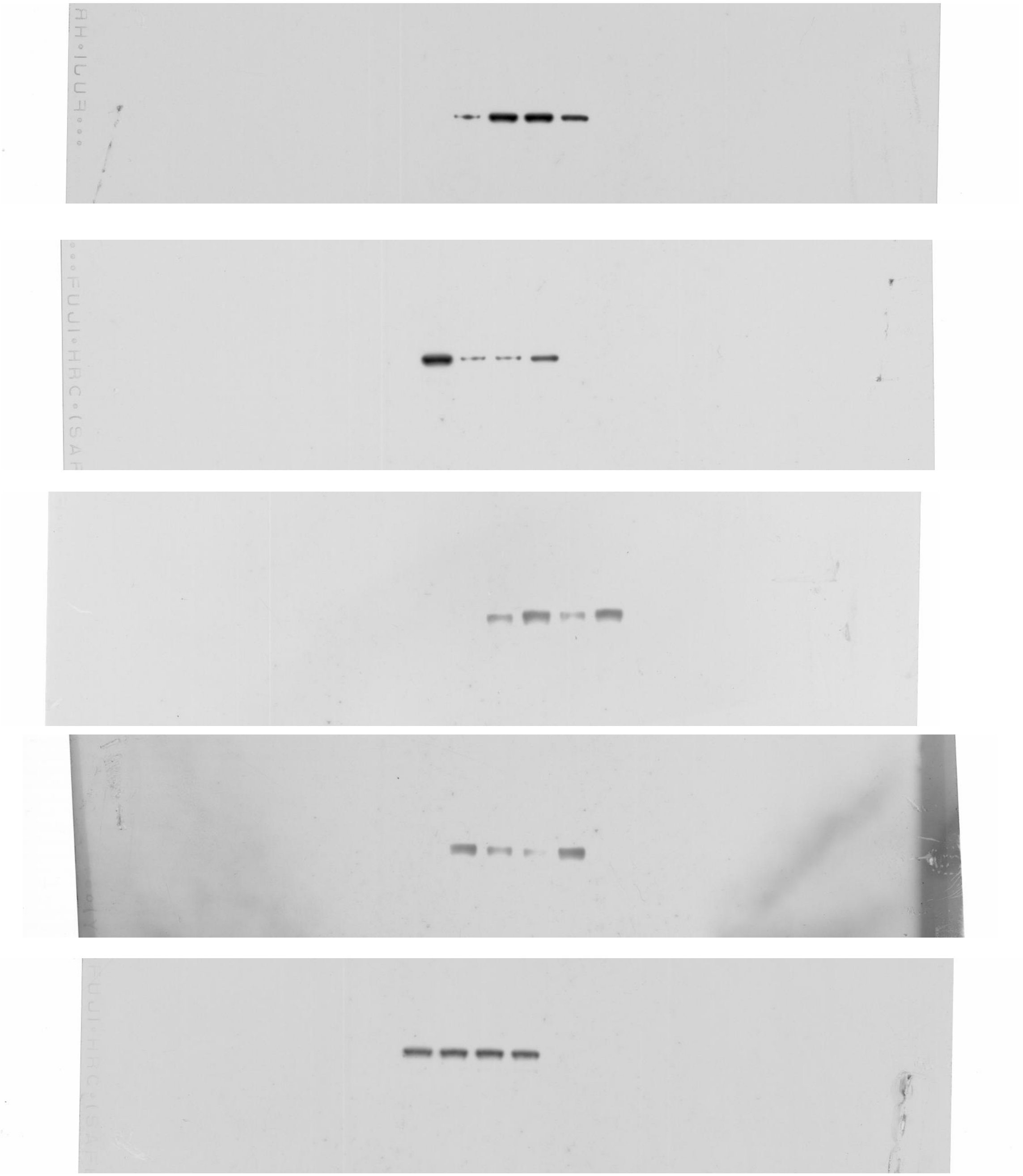

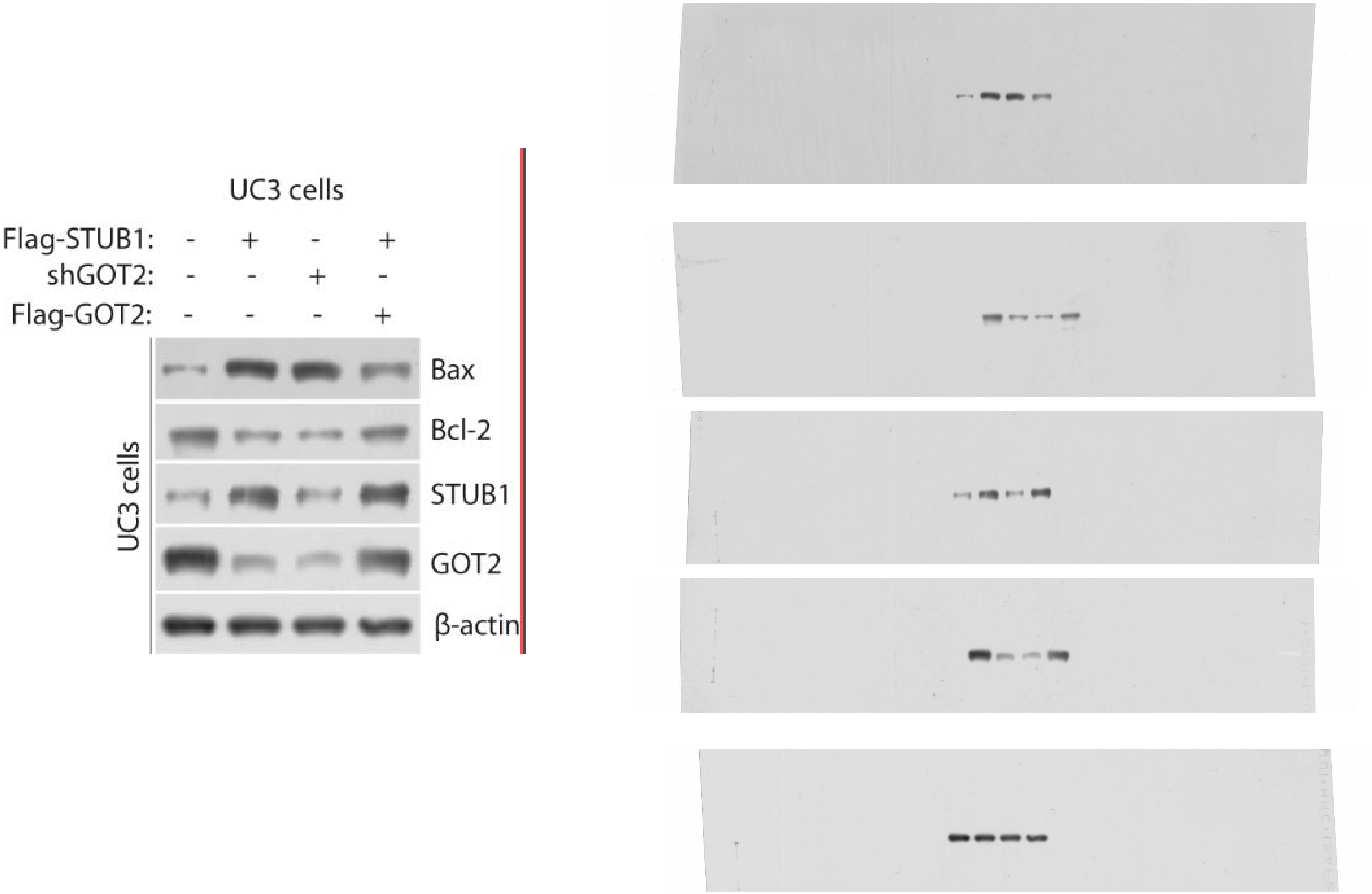
UC3 cells
Flag-STUB1: - + - +
shGOT2: - - + -
Flag-GOT2: - - - +
Bax
Bcl-2
STUB1
GOT2
β-actin
UC3 cells

Figure.S6

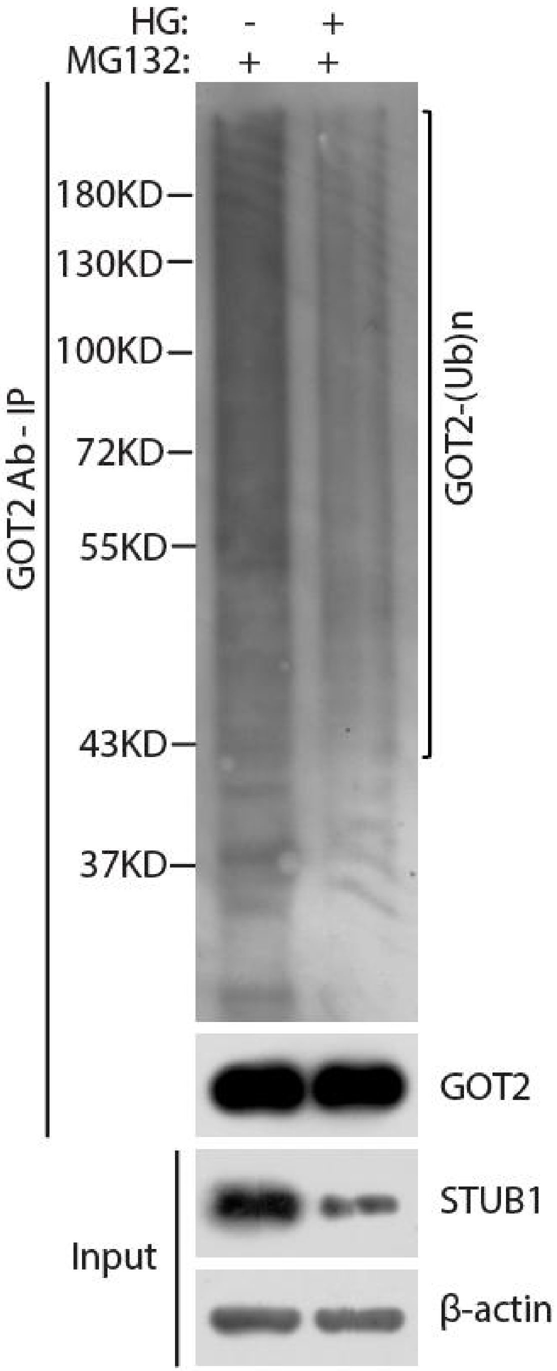
HG: - +
MG132: + +
180KD
130KD
100KD
72KD
55KD
43KD
37KD
GOT2 Ab - IP
GOT2-(Ub)n
GOT2
STUB1
β-actin
Input

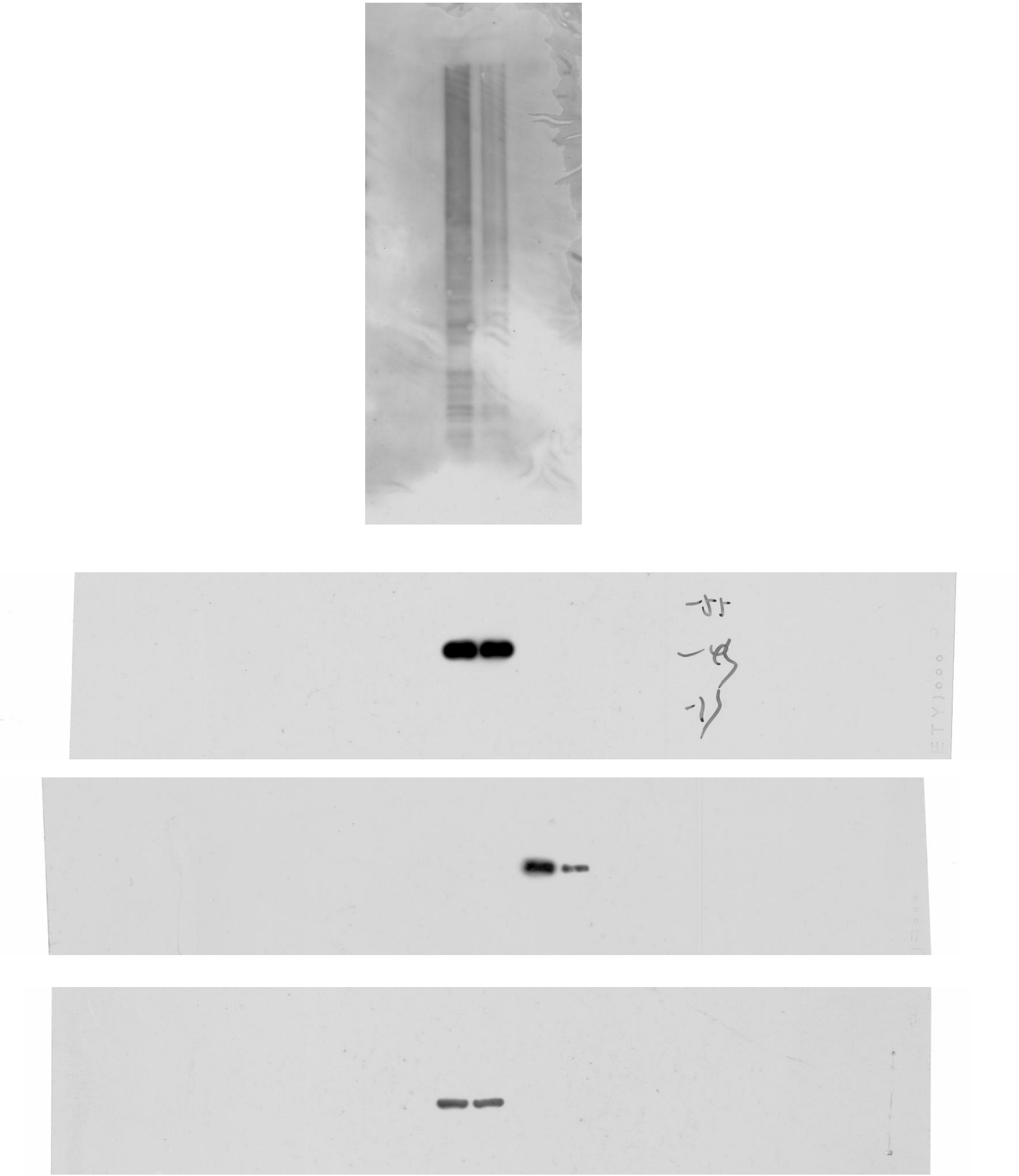

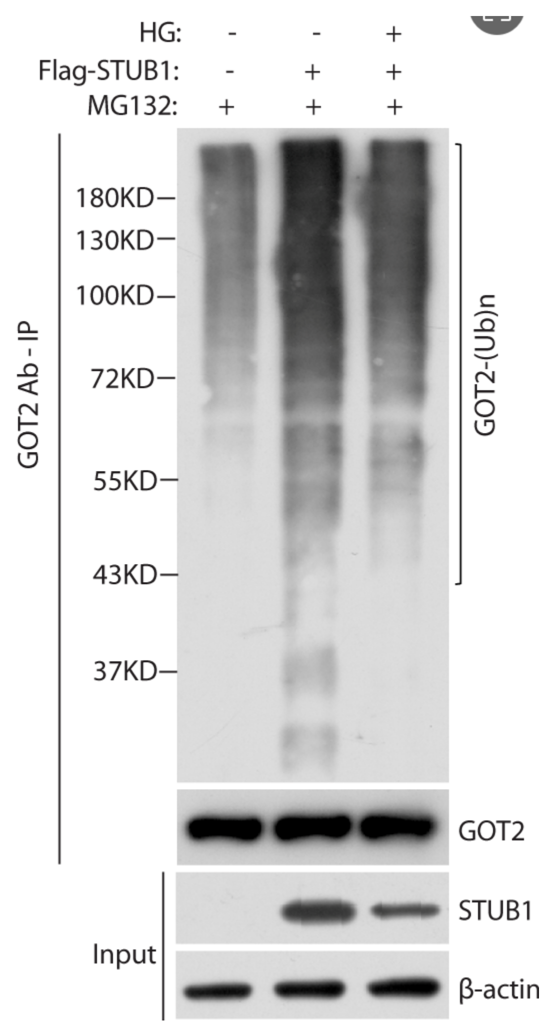
HG: - - +
Flag-STUB1: - + +
MG132: + + +
GOT2 Ab - IP
180KD
130KD
100KD
72KD
55KD
43KD
37KD
GOT2-(Ub)n
GOT2
STUB1
Input
β-actin

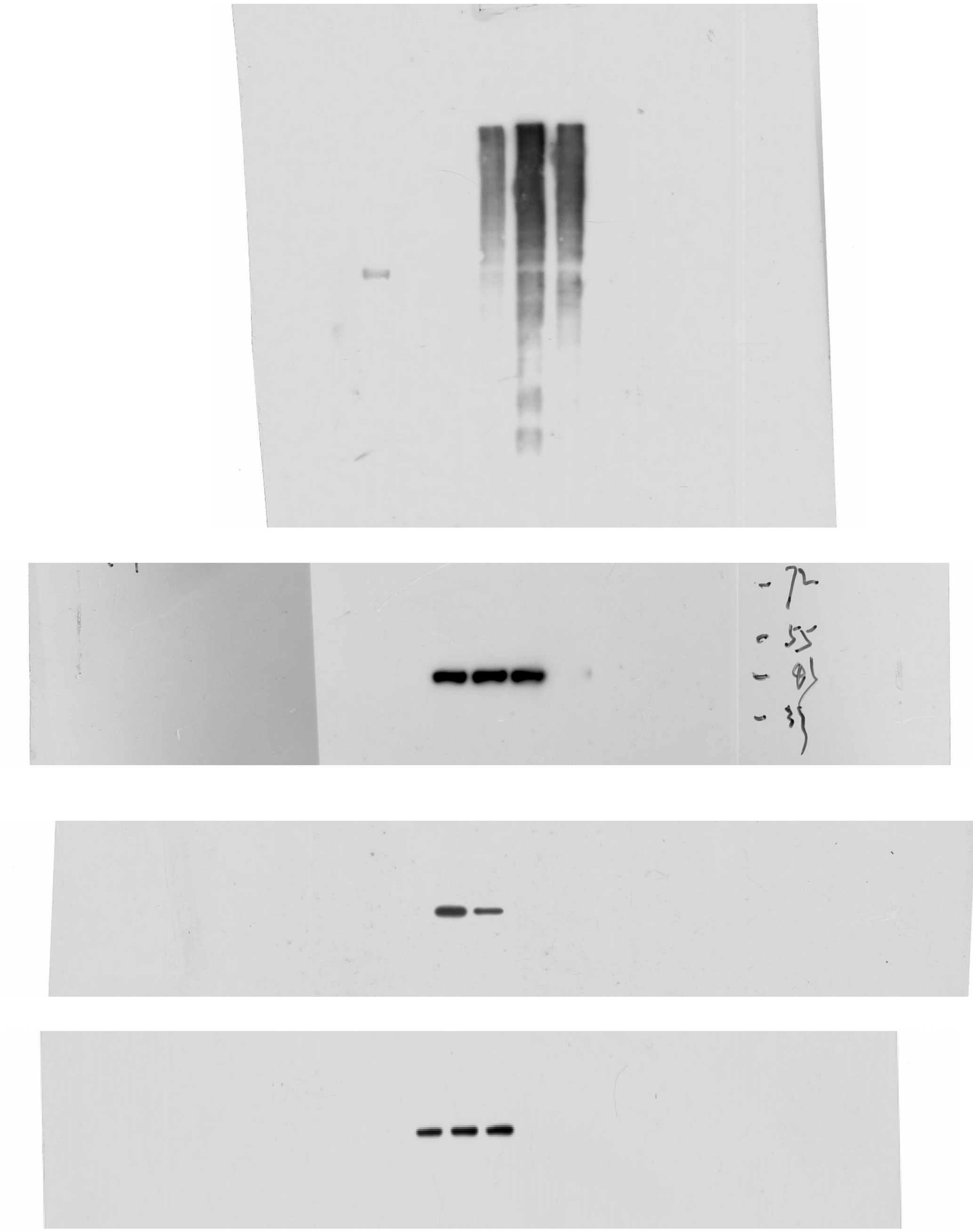

Supplement: Supplementary file 2 — original western blots [file 41419_2025_7840_MOESM2_ESM.pdf]
